# Identification of Transcription Factor Genes and Their Correlation with the High Diversity of Stramenopiles

**DOI:** 10.1371/journal.pone.0111841

**Published:** 2014-11-06

**Authors:** Francisco Javier Buitrago-Flórez, Silvia Restrepo, Diego Mauricio Riaño-Pachón

**Affiliations:** 1 Group of Computational and Evolutionary Biology, Universidad de los Andes, Bogotá, Colombia; 2 Micology and Plant Pathology Laboratory, Universidad de los Andes, Bogotá, Colombia; 3 Laboratório Nacional de Ciência e Tecnología do Bioetanol (CTBE), Centro Nacional de Pesquisa em Energia e Materiais (CNPEM), Campinas, Brasil; St. Georges University of London, United Kingdom

## Abstract

The biological diversity among Stramenopiles is striking; they range from large multicellular seaweeds to tiny unicellular species, they embrace many ecologically important autothrophic (e.g., diatoms, brown algae), and heterotrophic (e.g., oomycetes) groups. Transcription factors (TFs) and other transcription regulators (TRs) regulate spatial and temporal gene expression. A plethora of transcriptional regulatory proteins have been identified and classified into families on the basis of sequence similarity. The purpose of this work is to identify the TF and TR complement in diverse species belonging to Stramenopiles in order to understand how these regulators may contribute to their observed diversity. We identified and classified 63 TF and TR families in 11 species of Stramenopiles. In some species we found gene families with high relative importance. Taking into account the 63 TF and TR families identified, 28 TF and TR families were established to be positively correlated with specific traits like number of predicted proteins, number of flagella and number of cell types during the life cycle. Additionally, we found gains and losses in TF and TR families specific to some species and clades, as well as, two families with high abundance specific to the autotrophic species and three families with high abundance specific to the heterotropic species. For the first time, there is a systematic search of TF and TR families in Stramenopiles. The attempts to uncover relationships between these families and the complexity of this group may be of great impact, considering that there are several important pathogens of plants and animals, as well as, important species involved in carbon cycling. Specific TF and TR families identified in this work appear to be correlated with particular traits in the Stramenopiles group and may be correlated with the high complexity and diversity in Stramenopiles.

## Introduction

Stramenopiles are a very diverse group that includes algae, diatoms, as well as the non-photosynthetic oomycetes, and a range of chlorophyll c-containing unicellular and multi-cellular brown algae [1]. On one hand, there are Bacillariophyceae and Chrysophyceae, secondarily photosynthetic organisms having engulfed red algae and adopted it as a plastid approximately 1,300 million years ago [2]. On the other hand, non-photosynthetic Stramenopiles such as the oomycetes, do not even have the vestigial plastids found in apicomplexan and euglenoid parasites that originated from phototrophs [2].

In all organisms, the regulation and coordination of growth, development, and cell cycle progression, as well as the physiological and metabolic adaptation to a variable environment, depend on the regulation of gene expression. A major step in modulation is controlling when and how much RNA is generated from a DNA template. Sequence specific DNA-binding transcription factors (TFs) each recognizing a family of *cis*-regulatory DNA sequences, regulate spatial and temporal gene expression by binding to DNA and either activating or repressing the action of the RNA polymerase [3]. Additional proteins, other transcriptional regulators (TRs), are involved in protein-protein interactions or chromatin remodelling [4]. TFs and TRs are modular in nature, composed of structural semi-independent units called domains that can appear in various combinations and arrangements [5], i.e., domain architecture.

Domain architectures have been used in order to identify TFs and TRs in different approaches [4]–[6]. Pérez-Rodriguez *et al*. (2010) described a number of TF families according to their domain architecture (rules system), identifying all domains which are known to occur in this type of regulatory proteins and that are generally employed to classify proteins as transcriptional regulators, making a database with putatively complete sets of TFs and TRs from several plant species [4].

In this study we identified and classified TFs and TRs of Stramenopiles which genome sequences are available, based on an extension of the rule system designed by Pérez-Rodriguez *et al*. (2010) [4]. Furthermore, we were interested in evaluating the correlation, in a proper phylogenetic context, between traits of interest and gene family sizes as a way to offer insights into the processes generating the observed diversity in Stramenopiles.

## Methods

### Identification and classification system

In order to identify TF and TR family members in the genome sequences of Stramenopiles, in a first step, we extended the set of rules established by Pérez-Rodriguez *et al*., (2010) [4], in order to recover specific families for groups such as Stramenopiles and Fungi. Briefly, we identified, using current literature, the set of domains that were known to occur in TFs and TRs from Stramenopiles and Fungi (a list of a total of 49 new rules is available in the Table S1). This list was established from the available PFAM profile HMMs (v25.0). Additionally, we generated new, or updated HMMs (*in house* models), based on alignments that were created with outputs of PSI-BLAST searches against the NCBI protein database or found in the literature.

To carry out the identification and classification of TFs and TRs, we retrieved the entire set of predicted proteins of all Stramenopiles species which nuclear genomes are sequenced and annotated, available at the end of 2010 (Table 1). A profile HMM search with hmmsearch (HMMER v3.0) was performed over all protein sequences using all protein domain models (PFAM v25.0 and *in house* models). We considered as significant all hits with a bit-score larger than the domain gathering cut-off value defined in the model. We classified the proteins into the established families of TFs and TRs based on their domain architecture i.e., the set of rules (Figure S1).

**Table 1:** Stramenopile species included in this study.

Organism	Size	Genes	Proteins	Source
***Phytophthora infestans*** ** v 1.0**	237 Mb	18179	18140	BROAD institute
***Phytophthora ramorum*** ** v 1.1**	65 Mb	15743	16066	DOE-JGI institute
***Phytophthora capsici*** ** v 11.0**	64 Mb	19805	15919	DOE-JGI institute
***Phytophthora sojae*** ** v 4.0**	86 Mb	19027	19276	DOE-JGI institute
***Saprolegnia parasitica*** ** v 1.0**	53.09 Mb	20113	20088	BROAD institute
***Hyaloperonospora parasitica*** ** v 8.3**	82.05 Mb	14567	14565	BROAD institute
***Pythium ultimum*** ** v 1.0**	44.91 Mb	15291	12614	BROAD institute
***Thalassiosira pseudonana*** ** v 3.0**	32 Mb	11397	11318	DOE-JGI institute
***Phaeodactylum tricornutum sojae*** ** v 2.0**	28 Mb	10489	10389	DOE-JGI institute
***Fragilariopsis cylindrus*** ** v 1.0**	80,5 Mb	27137	27137	DOE-JGI institute
***Aureococcus anophagefferens*** ** v 1.0**	56.7 Mb	11501	11501	DOE-JGI institute

After the identification and classification of TFs and TRs, we carried out a relative importance analysis. This analysis measures how important a family is in comparison with other families in the same organism, according to its size, i.e., we represented the importance of the family in a given species, as its percentual contribution in that species over the total number of TFs and TRs.

### Updating *in house* models

For all *in house* models we created our own HMM profile with HMMER 3.0 based on multiple sequence alignments. Furthermore, we defined a gathering cut-off value beyond which the hits are considered significant, to this end, we used the same procedure as described in Pérez-Rodriguez *et al*. (2010) [4].

In order to verify our models, we carried out the identification and classification of TFs and TRs in *Arabidopsis thaliana* to find out whether the use of HMM-models now created with HMMER 3.0 shows the same classification as shown in Pérez-Rodriguez *et al*. (2010) [4].We expected to retrieve at least the same members for each of the protein families reported in Pérez-Rodriguez *et al*., (2010) [4]. Seed alignments and models are available as Datasets S1 and S2, respectively.

### Phylogenomic analysis of Stramenopiles

We inferred the phylogenetic relationships between the species of Stramenopiles using orthologous genes identified with OrthoMCL (http://orthomcl.org/) [7]. We carried out two extra OrthoMCL analyses, one of them including selected protist species, the other one including the same selected protist species and some plant species, all the extra species were selected taking into account their phylogenetic relationships and that their genomes were fully sequenced (Table 2). These OrthoMCL analyses were made in order to determine whether the inclusion of distant species had any effect on the tree topology, the number of phylogenetic clusters retrieved in each OrthoMCL analysis is shown in Table 3. To establish the groups of orthologous genes we used a MCL inflation value of 1.5. Two datasets were retrieved: one with all orthoMCL clusters with one and only one orthologous gene for each species, and the second, with all clusters in which at most one species was missing. For each retrieved cluster we performed a multiple sequence alignment with MAFFT (—auto default option) [8], and we determined the proper evolutionary model using ProtTest (default options) [9]. Alignments were concatenated using FasConCat [10]. Phylogenetic inference was performed with FastTree [11] and RAxML [12] for the super-matrix approach with 1000 bootstrap replicates/samples, in a maximun likelihood analysis. All the matrices and trees from RAxML that were used for subsequent analysis were uploaded in TreeBase (http://purl.org/phylo/treebase/phylows/study/TB2:S12312). Furthermore, we used the PANTHER library version 7.0 (http://www.pantherdb.org/) to assign putative funtional annotation to the gene clusters used for the phylogenomic reconstruction.

**Table 2:** Species included in OrthoMCL analysis.

Stramenopiles	Protists
*Phytophthora infestans v 4.0 - BROAD institute*	*Plasmodium berghei – Ensemble Genomes*
*Phytophthora ramorum v 1.1 - DOE-JGI Institute*	*Plasmodium chabaudi – Ensemble Genomes*
*Phytophthora capsici v 11.0 1 - DOE-JGI Institute*	*Plasmodium falciparum – Ensemble Genomes*
*Phytophthora sojae v 4.0 1 - DOE-JGI Institute*	*Plasmodium knowlesi – Ensemble Genomes*
*Saprolegnia parasítica v 1.0 - BROAD institute*	*Plasmodium vivax – Ensemble Genomes*
*Hyaloperonospora parasitica v 8.3 - BROAD institute*	*Dictyostelium discoideum – Ensemble Genomes*
*Pythium ultimum v 1.0 - BROAD institute*	**Plants******
*Thalassiosira pseudonana v 3.0 1 - DOE-JGI Institute*	*Cyanidioschyzon merolae – Ensemble Genomes*
*Phaeodactylum tricornutum v 2.0 1 - DOE-JGI Institute*	*Ostreococcus tauri – Ensemble Genomes*
*Fragilariopsis cylindrus v 1.0 1 - DOE-JGI Institute*	*Chlamydomonas reinhardtii – Ensemble Genomes*
*Aureococcus anophagefferens v 1.0 1 - DOE-JGI Institute*	

**Table 3:** Number of phylogenetic clusters generated with OrthoMCL.

OrthoMCL - Stramenopiles			OrthoMCL - Stramenopiles + Protist		
MCL 1.0	MCL 1.5	MCL 2.0	MCL 1.0	MCL 1.5	MCL 2.0
136 Clusters	168 Clusters	167 Clusters	67 Clusters	73 Clusters	74 Clusters
MCL 1.0 (−1)	MCL 1.5 (−1)	MCL 2.0 (−1)	MCL 1.0 (−1)	MCL 1.5 (−1)	MCL 2.0 (−1)
180 Clusters	223 Clusters	217 Clusters	65 Clusters	88 Clusters	72 Clusters

OrthoMCL - Stramenopiles + Protist + Plants		
MCL 1.0	MCL 1.5	MCL 2.0
45 Clusters	52 Clusters	51 Clusters
MCL 1.0 (−1)	MCL 1.5 (−1)	MCL 2.0 (−1)
39 Clusters	56 Clusters	57 Clusters

The number of clusters is showed for each MCL value (1.0, 1.5, 2.0). For each of the inflation values we also analyzed the dataset with one species (gene) missing. (1.0 (−1), 1.5 (−1), 2.0 (−1).

### Comparative phylogenetics of Stramenopiles

One of our goals was to detect whether there were any traits that co-varied with gene family sizes, as this will suggest a clear relationship between the given trait and gene family, thus prioritizing families for further studies and experiments. In order to assess the correlation between genomic and phenotypic characters and the sizes of different TF and TR families in Stramenopiles we built a trait matrix including 4 traits: genome size, number of predicted proteins, number of flagella and number of cell types in the life cycle (Table S2). The evaluation of the co-variation between the traits and the gene family size, might be done with a simple correlation analysis. However, this assumes that the observations, data points, are independent and identically distribute, e.g., number of flagella in different species; but we know that the species under study are related by common decent, thus violating the assumption required for a simple correlation analysis. We can correct for the common evolutionary history, transforming our data points into an, in principle, independent and identically distributed dataset using Phylogenetic Independent Contrast analysis (PIC) [13]–[15], and then perform the correlation analysis, over these contrasts. In this way, using PIC, we can evaluate the correlation between traits and gene family size, controlling the effect of common evolutionary history. We perfomed a PIC for each of these traits, using the R package APE [16]. Finally, the Pearson's correlation coefficient was computed between the contrasts of interest, i.e., traits and the sizes of TF and TR families, using the basic function *cor()* in R [17]; p-values for the measured correlation coefficients were corrected using the false discovery rate approach in the R package fdrtool [18].

Furthermore, we mapped on the phylogenetic tree the gains, losses and high abundance of TF and TR families. Six additional characters, i.e., cell cover (Cellulose or silica cover), presence/absence of chloroplast, presence/absence of pigments, lifestyle, presence/absence of cell wall degrading enzymes and formation of haustorium were also mapped on the tree (Table S2). Mapping family sizes and traits was perfomed using Mesquite (http://mesquiteproject.org/). In order to identify correlations between the family size and the 6 additional characters, we used a phylogenetic logistic regression with the firth correction using the PlogReg algorithm, this method was necessary since the additional 6 characters are categorical [19]. Moreover, significance of high abundance differences in TFs and TRs families between the autotrophic and the heterotrophic groups was analyzed using standard t-test with subsequent false discovery rate correction.

## Results

### Extension of the rules system for the identification and classification of TF and TR families in Stramenopiles

From a thorough literature search, we designed 135 rules for classifying regulatory proteins. From the entire set of rules, 75% were related with domains occuring in plants from the previous work of Pérez-Rodriguez *et al*. (2010) [4]. This set of rules comprises a wide array of families widely distributed across eukaryotes. The other 25% of the rules were built with domains occurring in TFs and TRs of Stramenopiles and/or fungi (Figure S1).

For the domains Alfin-like, CCAAT-Dr1, DNC, G2-like, HRT, LUFS, NF-YB, NF-YC, NOZZLE, SAP, Topless, Trihelix, ULT, VARL and VOZ we updated the hidden markov models with the latest release of HMMER (v3.0).

### TFs and TRs found in Stramenopiles

**Figure 1:**
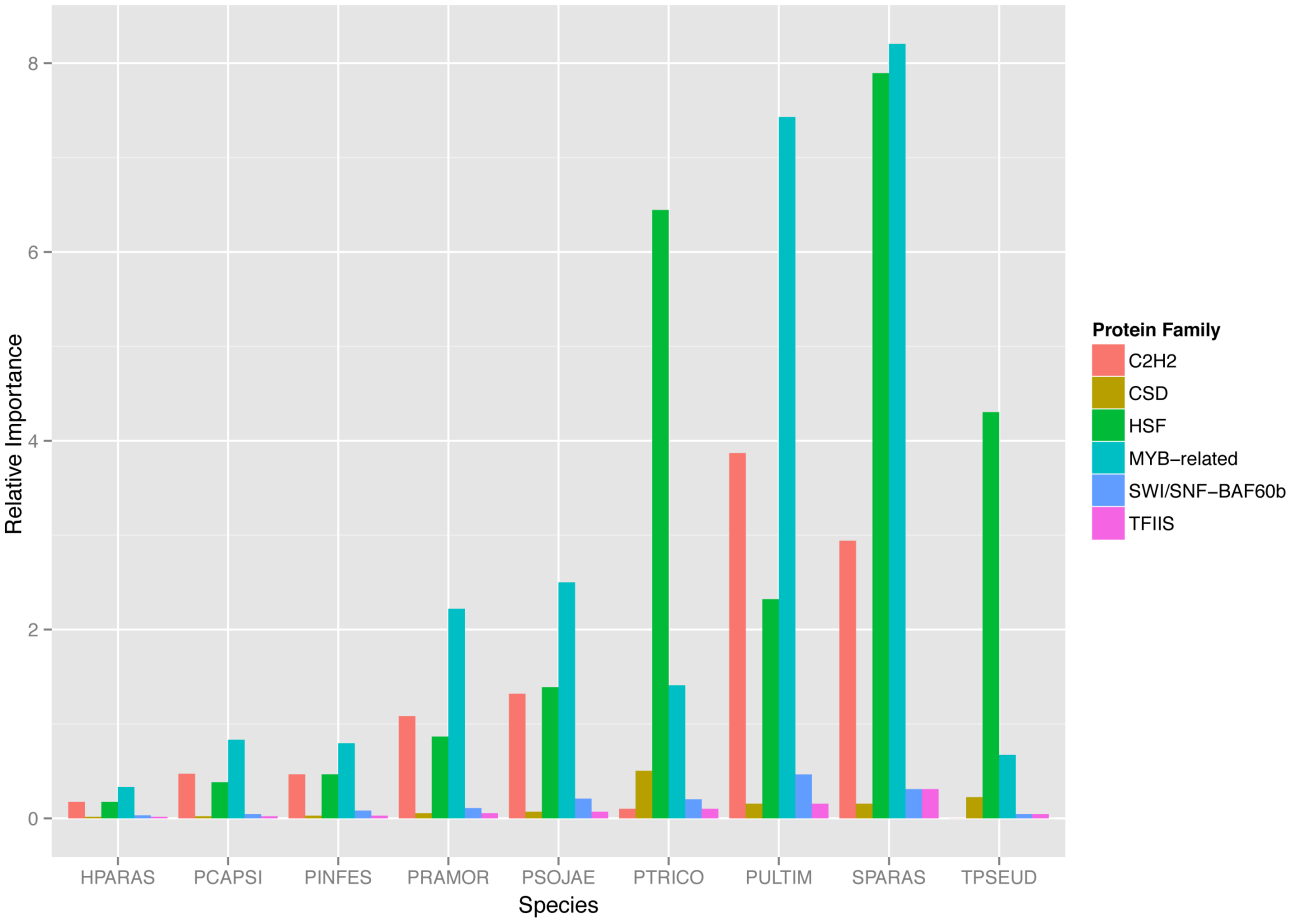
Relative Importance of TF and TR families in Stramenopiles. The Y axis represents the relative importance of each family, the X axis represents the 9 species used in this study. For each species, families with a high relative importance and correlated with the number of predicted proteins, number of cell types and number of flagella are shown (C2H2 in orange, HSF in green in and MYB-related in blue). Moreover, some families with a low relative importance are shown in the figure as a control for comparison (CSD in yellow, SWI/SNF-BAF60b in purple and TFIIS in pink).

We found 38 TF and 25 TR families in the 11 Stramenopiles evaluated, i.e., about 50% of the total of rules included (Table S3). The relative importance analysis (Figure 1), shows a comparison between the families with a high relative importance like GNAT (proteins with acetyltransferase domain), MYB-related (DNA binding domains from Myb proteins), and C2H2 (protein structural zinc finger that bind DNA, RNA or proteins) with contrasting families with low relative importance across the species, such as CSD, SWI/SNF-BAF60b and TFIIS.

### Phylogenomic analysis of Stramenopiles

**Figure 2:**
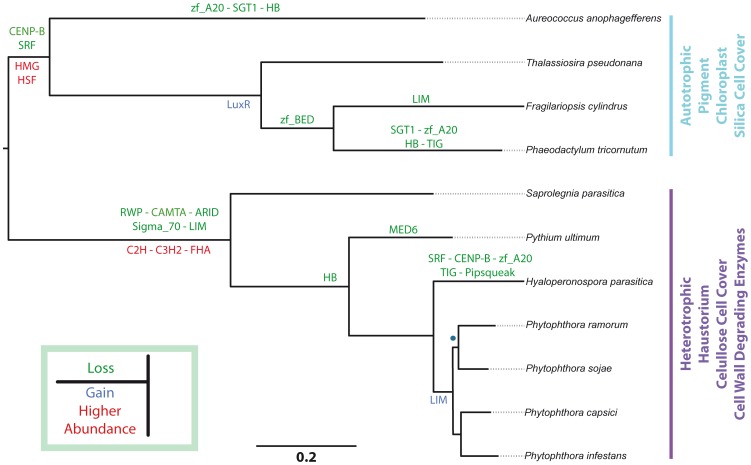
Reconstruction of evolutionary relationships among Stramenopiles and Mapping of TF and TR families on the tree. Orthologous gene sets were identified using OrthoMCL with a MCL inflation value of 1,5. All the clusters with at most one orthologous gene for each species of Stramenopiles were selected for phylogenetic inference, see the main text for details. All clades have a bootstrap support of 1, except the clade with a blue dot, which has a bootstrap value of 0.986. Gene family losses appear in green and gains are in shown in blue. Families that differ in size between the autothrofic and the heterothrophic group appear in red. In cyan and purple, some traits characteristic of autothrophic and heterothrophic organisms, respectively.

We could show that the inference of evolutionary relationships among Stramenopiles is robust to the inclusion of distantly related species as protists and algae plants (Figure S2). Noteworthy, we found that red and green algae are closely related to Stramenopiles, as suggested previously [20]–[22]. For further analyses we used the tree inferred including all the orthologous gene clusters, where at most, one gene representing each species was present (orthologues in a one to one relationship), and using an inflation value of 1.5, as recommended previously [23] (Figure 2; Dataset S3). We were able to assing functions to 45% of the ortologous gene clusters using the PANTHER library, examples of these gene families are Heat shock protein 70, Histone acetyltransferase, Ornithine decarboxylase and TATA-box-binding protein, however, a large number of cluster genes were not identified with this approach (details in Table S4).

### Comparative phylogenetics of Stramenopiles: Trait correlations with TF and TR family sizes

**Figure 3:**
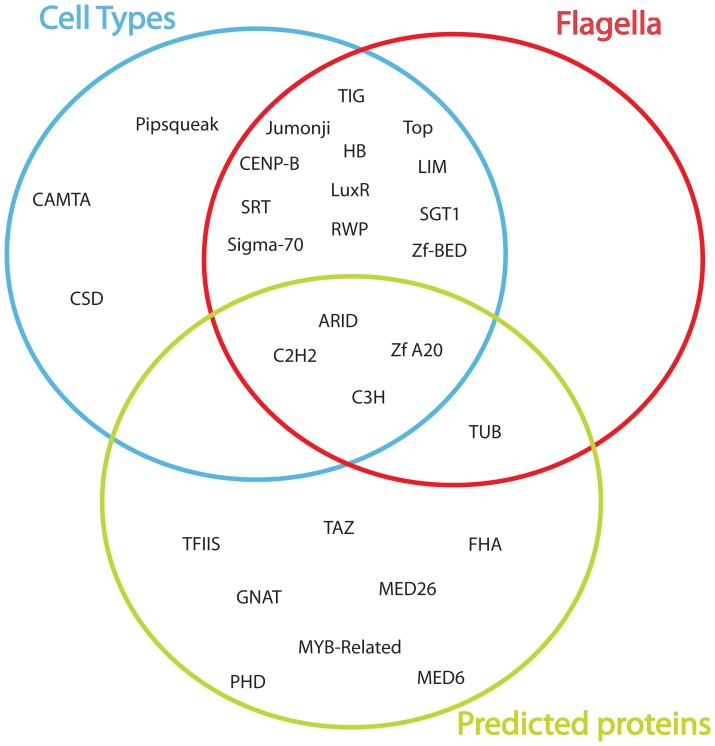
Families correlated with traits after phylogenetic independent contrasts analysis and false discovery rate correction. Colors represent each trait analyzed: in blue, number of cell types during the cell cycle; in red, presence or absence of flagella; and in green, number of predicted proteins encoded by the nuclear genome. Genome size does not show correlation with any trait.

Out of the 63 TF and TR families identified in Stramenopiles, we found a correlation between 28 families with the presence of flagella, number of predicted proteins and number of cell types in the life cycle (Table S5). Moreover, we found no correlation with genome size; this is an expected result due to the high variability of the genome sizes in the group, even more when taking into account the c-value paradox [24]. The PIC analysis identified a common set of families that are correlated with all traits (Figure 3), making them a candidate set responsible for the biological diversity shown by this group of organisms. A different subset of families was correlated with the formation of flagella and number of cell types together. Moreover, there are several families specifically correlated with the number of predicted proteins. Three families specifically correlated with the number of cell types in the life cycle (Figure 3). Taking into account the relative importance analysis described previously (Figure 1) we found that there are families showing a correlation and at the same time a high relative importance, as in the case of C2H2, GNAT and MYB families.

Mapping families on the phylogenetic tree allowed us to identify gains and losses. Losses are recurrent in different species and clades; however, in species like *Hyaloperonospora parasitica* and *Phaeodactylum tricornutum* we found a higher number of them. There are families that were lost more than once, as in the case of zf_A20, HB and TIG families (Figure 2). On the other hand, we found that the transcription factor family LuxR (related with the expression of response regulators in several bacteria species) was gained in 3 autotrophic species (Figure 2). It was reported that a remarkably high number of genes appear to have been transferred between Stramenopile diatoms and bacteria via horizontal gene transfer (HGT) [25]. We carried out a phylogenetic inference (RAxML – 1000 Bootstraps - evolutionary model Gamma+I+G) using the eukaryotic versions of LuxR and the corresponding versions from *Vibrio cholera*, *Vibrio alginolyticus* and *Bacillus cereus*, revealing that the eukaryotic LuxR forms a single, well supported, clade. This clearly indicates that if a horizontal gene transfer event took place, it was a single one predating the split between *Fragilariopsis cylindrus*, *Phaeodactylum Tricornutum* and *Thalassiosira pseudonana* (Figure S3).

We could also establish that the LIM family, found in other eukaryotes [26], was lost in the Oomcycete clade, and later re-gained in the *Phytophthora* group (Figure 2). Furthermore, high abundance in HMG and HSF families was significant for the autotrophic group, as high abundance in C2H2, C3H and FHA families was found significant for the heterotrophic group (Figure 2). Finally, no correlation was found between the TF and TR family size and the binary traits cell cover, presence/absence of chloroplast, presence/absence of pigments, lifestyle, presence/absence of cell wall degrading enzymes and formation of haustoria (Table S6).

## Discussion

The updated rule system designed by Pérez-Rodriguez *et al*. (2010) [4] allowed us to rigorously classify proteins into several and well-described families of TFs and TRs. This was a key phase in order to avoid the inclusion of false negatives. Furthermore, a phylogenomic analysis of the Stramenopiles species enabled us to reach a reliable approximation of their phylogenetics relationships. This was a key step to establish a correlation between different traits and TF and TR families, eliminating as much as possible the bias generated by common ancestry in order to obtain values that are statistically independent [13].

The number of predicted proteins, number of cell types during the life cycle and the number of flagella, showed a strong correlation with the size changes of several TFs and TRs families. Most of these families, however, were positively correlated with more that one specific trait, making it clear that these families are implied in the complexity of the organisms, but leaving for further analyses the elucidation of their specific roles in the regulation of these traits. On the other hand, there were several families correlating specifically with the number of cell types during the life cycle and the number of predicted proteins, families like MYB, MYB_related and CAMTA are examples. It is very remarkable that we found a high relative importance of these families in some organisms, making them of great interest for further investigation.

Gain and loss of TF and TR families have been reported to be determinant in the complexity and diversity of many organisms [27]. Stramenopiles species evaluated in this study show no exception to these findings. For example, we could suggest a gain of the LuxR family for some autotrophic species, most likely resulting from horizontal gene transfer between bacteria and Stramenopiles [25]. Also, we found an interesting behavior in the LIM family (domains that mediate protein-protein interactions), as it was apparently lost in the oomycete group, but later, it appeared as a gain in the *Phytophthora* clade. Moreover, we found several families lost in specific species like the heterotrophic organism *Hyaloperonospora parasitica*, and the autotrophic organisms *Aureococcus anophagefferens* and *Phaeodactylum tricornutum*. These families need to be investigated in depth, in order to understand whether these losses and gains have and impact in the diversity of specific groups. However, the lack of genome sequences of heterotrophic but non-parasitic organisms could mask more relations between TF and TR families and specific features of Stramenopiles.

On the other hand, high abundance in TF and TR families have been thoroughly analyzed in many groups across the tree of life and seem to correlate with the complexity and diversity in several organisms [27]. We also could establish this relation in the Stramenopiles, taking into account the comparison between the two largest groups (autotrophic and heterotrophic organisms). In protists organisms, the significant high abundance in HMG (TR family) could suggest a possible secondary adaptation as a transcription factor [28], besides, the high abundance in the HSF family is involved in the regulation of heat shock proteins, in order to respond to different type of stresses [28]. The C2H2 and C3H families have been reported in several eukaryotic organisms across the tree of life; the expansions of these families have been described as independent lineage specific expansions in parasitic organisms that may be involved in regulation of their specific characteristics [28]. The high abundance of these families reported in this study for heterotrophic group (oomycetes) could be implicated in the regulation of specific features of this group, i.e., parasitic features.

## Conclusions

The understanding of how Stramenopiles species regulate some of their distinct characteristics may be helpful in dealing with some of the species in this group that cause economic losses around the world, e.g., *Phytophthora* species, or play an important role in the carbon cycling, e.g., Diatoms. We showed for the first time that TF and TR families are correlated with specific traits that may be involved in the complexity and diversity of Stramenopiles. Besides, there are several losses established in the Stramenopile group, as well as, lineage specific high abundance in families related to autotrophic and heterotrophic organisms. Currently, there is a bias in the genome sequences that are available towards the pathogenic organisms. The inclusion of more autotrophic organisms, as also, the inclusion of heterotrophic but non-pathogenic species, i.e., *Salisapilia* spp., will make possible a more complete identification across the Stramenopiles group in order to clarify the gains, losses and high abundance of TF and TR families.

## Supporting Information

Dataset S1 Seed alignments for each of the *in house* created models. Alignments in FastA format, for each one of the models created or updated in this study. All the seed alignments are in a single compressed (zip) MS Word document.
(ZIP)

Dataset S2 *In house* created models. All the models created or updated in this study are compressed (zip) into this single HMM file. Models were created using HMMER version 3.0.
(ZIP)

Dataset S3 Orthologous gene clusters for phylogenetic reconstruction. All the gene clusters in a one to one relationship using an inflation value of 1,5 used for phylogenetic analyses are compressed into this zip file. This is a compressed (zip) FastA file, the header for each sequence follows the format: “ClusterID|Species|SequenceID”.
(ZIP)

Figure S1 Bipartite graph of rules for identification and classification of TFs and TRs. Blue squares represent TF families and stripped squares represent TR families. Domains are represented as circles in which yellow circles represent PFAM domains and orange circles represent *in house* domains. Moreover, continuous lines indicate required domains and dotted lines indicate forbidden domains. Updated from Riaño-Pachón *et al*., 2007.
(PDF)

Figure S2 Tanglegram to assess the effect of inclusion of distantly related species. Right: Phylogenomic reconstruction of Stramenopiles. Left: Phylogenomic reconstruction of Stramenopiles plus another protists and algae. The inclusion of another protists such as *Plasmodium* spp. and red and green algae in the phylogenomic reconstruction, does not affect the evolutionary relationships between Stramenopiles. Acronyms used: *Phytophthora infestans* – PINFES, *Phytophthora capsici* – PCAPSI, *Phytophthora sojae* – PSOJAE, *Phytophthora ramorum* – PRAMOR, *Hyaloperonospora parasítica* – HPARAS, *Pythium ultimum* – PULTIM, *Saprolegnia parasítica* – SPARAS, *Phaeodactylum tricornutum* – PTRICO, *Fragilariopsis cylindrus* – FCYLIN, *Thalassiosira pseudonana* – TPSEUD, *Aureococcus anophagefferens* – AANOPH, *Plasmodium bergheu* – PBERGH, *Plasmodium chabaudi* – PCHABA, *Plasmoduim falciparum* – PFALCI, *Plasmodium knowlesi* – PKNOW, *Plasmodium vivax* – PVIVAX, *Dictyostelium discoideum* – DDISCO, *Cyanidioschyzon merolae* – CMEROL, *Ostreocuccus tauri* – OTAURI, *Clamydomonas reinhardtii* – CREINH.
(PDF)

Figure S3 LuxR phylogeny. A maximum likelihood analysis via FastTree with 1000 bootstrap replicates was performed with proteins classified into the LuxR family from Stramenopiles (*Phaeodactylum tricornutum, Thalassiosira pseudonana* and *Fragilariopsis cylindrus*) and 3 selected bacteria (*Vibrio cholera, Vibrio alginolyticus* and *Bacillus cereus*). Highlighted in blue all the proteins identified in Stramenopiles that belong to the LuxR family.
(PDF)

Table S1 New rules for identification of TFs and TRs in Stramenopiles. 49 rules specific for Stramenopiles were identified using current literature. Models were used using available PFAM profile HMMs (v25.0) or created based on alignments (*in house* models). Additionally, for each family the domains that must appear in the family (type  =  should) and domains that must not appear (type  =  should not) are shown.
(XLSX)

Table S2 Traits used for phylogenetic independent contrast analysis (PIC) and traits mapped for each species. Continuous traits were used for the PIC analysis (Genome size, predicted proteins, number of flagella and cell types); on the other hand, binary traits were used for mapping (cell cover, chloroplast, pigments, lifestyle, cell wall degrading enzymes and haustorium).
(XLSX)

Table S3 Number of proteins assigned to a TF or TR family. Columns represent each species and rows represent each of the TF and TR families. For each family the number of proteins classified into it is displayed.
(XLSX)

Table S4 Classification of orthologous genes using the PANTHER. 45% of the orthologous genes were assigned to a specific family in the PANTHER library, however, 65% of the genes were not assigned to a specific family.
(XLSX)

Table S5 TF and TR families correlated after PIC + FDR. Families with q-value <0.05 – the ones positive or negatively correlated - are shown for each of the continuous traits used in this study.
(XLSX)

Table S6 P-values for phylogenetic logistic regression with Firth correction. P-values for each family after Firth correction are shown for each binary trait used in this study. Moreover, there are not families correlated since none of the p-values is <to 0.05.
(XLSX)

## References

[1] Andersen RA (2004) Biology and systematics of heterokont and haptophyte algae. Am J Bot 91: 1508–1522. 21652306 10.3732/ajb.91.10.1508

[2] Tyler BM, Tripathy S, Zhang X, Dehal P, Jiang RH, et al (2006) Phytophthora genome sequences uncover evolutionary origins and mechanisms of pathogenesis. Science 313: 1261–1266. 16946064 10.1126/science.1128796

[3] Latchman DS (2001) Transcription factors: bound to activate or repress. Trends Biochem Sci 26: 211–213. 11295539 10.1016/s0968-0004(01)01812-6

[4] Perez-Rodriguez P, Riano-Pachon DM, Correa LG, Rensing SA, Kersten B, et al (2010) PlnTFDB: updated content and new features of the plant transcription factor database. Nucleic Acids Res 38: D822–827. 19858103 10.1093/nar/gkp805 PMC2808933

[5] Zhang H, Jin J, Tang L, Zhao Y, Gu XC, et al (2011) PlantTFDB 2.0: update and improvement of the comprehensive plant transcription factor database. Nucleic Acids Res 39: D1114–D1117. 21097470 10.1093/nar/gkq1141 PMC3013715

[6] Riano-Pachon DM, Ruzicic S, Dreyer I, Mueller-Roeber B (2007) PlnTFDB: an integrative plant transcription factor database. BMC Bioinformatics 8: 42. 17286856 10.1186/1471-2105-8-42 PMC1802092

[7] Li L, Stoeckert CJ Jr, Roos DS (2003) OrthoMCL: identification of ortholog groups for eukaryotic genomes. Genome Res 13: 2178–2189. 12952885 10.1101/gr.1224503 PMC403725

[8] Katoh K, Misawa K, Kuma K, Miyata T (2002) MAFFT: a novel method for rapid multiple sequence alignment based on fast Fourier transform. Nucleic Acids Res 30: 3059–3066. 12136088 10.1093/nar/gkf436 PMC135756

[9] Abascal F, Zardoya R, Posada D (2005) ProtTest: selection of best-fit models of protein evolution. Bioinformatics 21: 2104–2105. 15647292 10.1093/bioinformatics/bti263

[10] Kuck P, Meusemann K (2010) FASconCAT: Convenient handling of data matrices. Mol Phylogenet Evol 56: 1115–1118. 20416383 10.1016/j.ympev.2010.04.024

[11] Price MN, Dehal PS, Arkin AP (2010) FastTree 2—approximately maximum-likelihood trees for large alignments. PLoS ONE 5: e9490. 20224823 10.1371/journal.pone.0009490 PMC2835736

[12] Stamatakis A (2006) RAxML-VI-HPC: maximum likelihood-based phylogenetic analyses with thousands of taxa and mixed models. Bioinformatics 22: 2688–2690. 16928733 10.1093/bioinformatics/btl446

[13] Felsenstein J (1985) Phylogenies and the comparative method. American Naturalist 125: 15. 10.1086/703055 31094602

[14] Garland T Jr, Ives AR (2000) Using the Past to Predict the Present: Confidence Intervals for Regression Equations in Phylogenetic Comparative Methods. Am Nat 155: 346–364. 10718731 10.1086/303327

[15] Garland T Jr, Bennett AF, Rezende EL (2005) Phylogenetic approaches in comparative physiology. J Exp Biol 208: 3015–3035. 16081601 10.1242/jeb.01745

[16] Paradis E, Claude J, Strimmer K (2004) APE: Analyses of Phylogenetics and Evolution in R language. Bioinformatics 20: 289–290. 14734327 10.1093/bioinformatics/btg412

[17] Team Rdc (2008) R: A language and environment for statistical computing.

[18] Strimmer K (2008) fdrtool: a versatile R package for estimating local and tail area-based false discovery rates. Bioinformatics 24: 1461–1462. 18441000 10.1093/bioinformatics/btn209

[19] Ives AR, Garland T (2010) Phylogenetic logistic regression for binary dependent variables. Syst Biol 59: 9–26. 20525617 10.1093/sysbio/syp074

[20] Bhattacharya D, Yoon HS, Hackett JD (2004) Photosynthetic eukaryotes unite: endosymbiosis connects the dots. Bioessays 26: 50–60. 14696040 10.1002/bies.10376

[21] Cavalier-Smith T (2003) Protist phylogeny and the high-level classification of Protozoa. European Journal of Protistology 39: 338–348.

[22] Cavalier-Smith T (2010) Deep phylogeny, ancestral groups and the four ages of life. Philos Trans R Soc Lond B Biol Sci 365: 111–132. 20008390 10.1098/rstb.2009.0161 PMC2842702

[23] Chen FM, Vermunt J, Roos D (2007) Assesing performance of orthology detection strategies applied to eukaryiotic genomes. PLoS ONE 2: 383. 10.1371/journal.pone.0000383 PMC1849888 17440619

[24] Gregory TR (2001) Coincidence, coevolution, or causation? DNA content, cell size, and the C-value enigma. Biol Rev Camb Philos Soc 76: 65–101. 11325054 10.1017/s1464793100005595

[25] Bowler C, Allen AE, Badger JH, Grimwood J, Jabbari K, et al (2008) The Phaeodactylum genome reveals the evolutionary history of diatom genomes. Nature 456: 239–244. 18923393 10.1038/nature07410

[26] Riano-Pachon DM, Correa LG, Trejos-Espinosa R, Mueller-Roeber B (2008) Green transcription factors: a chlamydomonas overview. Genetics 179: 31–39. 18493038 10.1534/genetics.107.086090 PMC2390610

[27] Lang D, Weiche B, Timmerhaus G, Richardt S, Riano-Pachon DM, et al (2010) Genome-wide phylogenetic comparative analysis of plant transcriptional regulation: a timeline of loss, gain, expansion, and correlation with complexity. Genome Biol Evol 2: 488–503. 20644220 10.1093/gbe/evq032 PMC2997552

[28] Iyer LM, Anantharaman V, Wolf MY, Aravind L (2008) Comparative genomics of transcription factors and chromatin proteins in parasitic protists and other eukaryotes. Int J Parasitol 38: 1–31. 17949725 10.1016/j.ijpara.2007.07.018

