# Supplementary material for: Identification of Transcription Factor Genes and Their Correlation with the High Diversity of Stramenopiles
Source: PLoS One. 2014 Nov 6;9(11):e111841. doi: 10.1371/journal.pone.0111841 (PMC4222949; doi:10.1371/journal.pone.0111841)
Supplement: Dataset S1 — Seed alignments for each of the in house created models. Alignments in FastA format, for each one of the models created or updated in this study. All the seed alignments are in a single compressed (zip) MS Word document. (ZIP) [file pone.0111841.s001.zip › Dataset S1.docx]

Family: Alfin-Like

>Alfin-Like_gi|15239624|ref|NP_197993_1| gi|15239624|ref|NP_197993.1| AL4 (ALFIN-LIKE 4); DNA binding / methylated histone residue binding [Arabidopsis thaliana] 267 bp

MEAGGA-YNPRTVEEVFRDFKGRRAGMIKALTTDVQEFFRLCDPEKENLCLYGHPNEHWE

VNLPAEEVPPELPEPVLGINFARDGMAEKDWLSLVAVHSDAWLLAVAFFFGARFGFDKAD

RKRLFNMVNDLPTIFEVVAGTAKKQGKDKSSVSNNSSNRSKSSSK-------RGSESRAK

---FSKPEPKDDEEEEEEGVEEEDEDEQGETQCGACGESYAADEFWICCDLCEMWFHGKC

VKITPARAEHIKQYKCPSCSNKRA-RS

>Alfin-Like_gi|3319341|gb|AAC26230_1| gi|3319341|gb|AAC26230.1| similar to Medicago sativa nucleic acid binding protein Alfin-1 (GB:L07291) [Arabidopsis thaliana] 267 bp

MEAGGA-YNPRTVEEVFRDFKGRRAGMIKALTT-----------EKENLCLYGHPNEHWE

VNLPAEEVPPELPEPVLGINFARDGMAEKDWLSLVAVHSDAWLLAVAFFFGARFGFDKAD

RKRLFNMVNDLPTIFEVVAGTAKKQGKDKSSVSNNSSNRSKSSSKVETLFSTRGSESRAK

---FSKPEPKDDEEEEEEGVEEEDEDEQGETQCGACGESYAADEFWICCDLCEMWFHGKC

VKITPARAEHIKQYKCPSCSNKRA-RS

>Alfin-Like_gi|297738599|emb|CBI27844_3| gi|297738599|emb|CBI27844.3| unnamed protein product [Vitis vinifera] 267 bp

MDGGPQ-YNPRTVEEVFRDFKGRRAGMIKALTTDVEEFYQQCDPEKENLCLYGFPNELWE

VNLPAEEVPPELPEPALGINFARDGMQEKDWLSLVAVHSDAWLLAVAFYFGARFGFDKAD

RKRLFNMINDLPTIFEVVTGAAKKQVKEKSSVSNHSSNKSKSNSKV------RGSESAKY

---LKGGQPKD----EEEGLDEVDEEEHGDTLCGACGENYASDEFWICCDICEKWFHGKC

VKITPARAEHIKQYKCPSCSNKRA-RP

>Alfin-Like_gi|15229157|ref|NP_189865_1| gi|15229157|ref|NP_189865.1| AL3 (ALFIN-LIKE 3); DNA binding / methylated histone residue binding [Arabidopsis thaliana] 267 bp

MEGGAALYNPRTVEEVFKDFKGRRTAIVKALTTDVQEFYQQCDPEKENLCLYGLPNEEWE

VNLPAEEVPPELPEPALGINFARDGLSEKEWLSLVAIHSDAWLLSVSFYFGSRFSFHKEE

RKRLFNMINDVPTIFEVVTGMAK--AKDKSSAANQNGNKSKSNSKV------RTSEGKSS

----KTKQPKE----EDEEIDEDDEDDHGETLCGACGDSDGADEFWICCDLCEKWFHGKC

VKITPARAEHIKQYKCPSCSNKRA-RA

>Alfin-Like_gi|15226967|ref|NP_178351_1| gi|15226967|ref|NP_178351.1| AL6 (ALFIN-LIKE 6); DNA binding / methylated histone residue binding [Arabidopsis thaliana] 267 bp

MEGITH-PIPRTVEEVFSDFRGRRAGLIKALTNDMVKFYQTCDPEKENLCLYGLPNETWE

VNLPVEEVPPELPEPALGINFARDGMQEKDWVSLVAVHSDSWLLSVAFYFGARFGFGKNE

RKRLFQMINELPTIFEVVSGNAK-QSKDLSV-NNNNSKSKPSGVKS------RQSESLSK

VAKMSSPPPKEEEEEEDESEDESEDDEQG-AVCGACGDNYGTDEFWICCDACEKWFHGKC

VKITPAKAEHIKHYKCPTCSNKRA-RP

>Alfin-Like_gi|15223786|ref|NP_172903_1| gi|15223786|ref|NP_172903.1| AL7 (ALFIN-LIKE 7); DNA binding / methylated histone residue binding [Arabidopsis thaliana] 267 bp

MEGIQH-PIPRTVEEVFSDFRGRRAGLIKALSTDVQKFYHQCDPEKENLCLYGLPNETWE

VNLPVEEVPPELPEPALGINFARDGMQEKDWISLVAVHSDSWLISVAFYFGARFGFGKNE

RKRLFQMINDLPTIFEVVTGNAK-QSKDQSA-NHNSSRSKSSGGKP------RHSESHTK

ASKMSPPPRK-----EDESGDEDEDDEQG-AVCGACGDNYGGDEFWICCDACEKWFHGKC

VKITPAKAEHIKHYKCPSCTTSKKMKA

>Alfin-Like_gi|15239180|ref|NP_196180_1| gi|15239180|ref|NP_196180.1| AL1 (ALFIN-LIKE 1); DNA binding / methylated histone residue binding [Arabidopsis thaliana] 267 bp

M--AAESSNPRTVEEIFKDFSGRRSGFLRALSVDVDKFYSLCDPEMENLCLYGHPNGTWE

VNLPAEEVPPELPEPALGINFARDGMQRKDWLSLVAVHSDCWLLSVSSYFGAR--LNRNE

RKRLFSLINDLPTLFEVVTGRK--PIKDGKP-SMDLGSKSRNGVK-------RSIEGQTK

----STPKLME----ESY---EDEDDEHGDTLCGSCGGNYTNDEFWICCDVCERWYHGKC

VKITPAKAESIKQYKCPSCCTKKG-RQ

>Alfin-Like_gi|15229696|ref|NP_187729_1| gi|15229696|ref|NP_187729.1| AL2 (ALFIN-LIKE 2); DNA binding / methylated histone residue binding [Arabidopsis thaliana] 267 bp

MAAAAVSSNPRTVEEIFKDYSARRAALLRALTKDVDDFYSQCDPEKENLCLYGHPNESWE

VNLPAEEVPPELPEPALGINFARDGMQRKDWLSLVAVHSDCWLLSVSFYFGAR--LNRNE

RKRLFSLINDLPTLFDVVTGRK--AMKDNKP-SSDSGSKSRNGTK-------RSIDGQTK

---SSTPKLME----ESYE-EEEEEDEHGDTLCGSCGGHYTNEEFWICCDVCERWYHGKC

VKITPAKAESIKQYKCPPCCAKKG-RQ

Family: CCAAT-Dr1

>CCAAT-Dr1_At5g08190 163 bp

MDPMDIVGKSKEDASLPKATMTKIIKEMLPADVRVARDAQDLLIECCVEFINLISSESNE

VCNKEDKRTIAPEHVLKALQVLGFGEYVEEVYAAYEQHKYETMQDSQRSVKMNSGAEMTE

EEAAAEQQRMFAEARARMNGGVTVPQPEQLEEPQQQQQTSLQS

>CCAAT-Dr1_At5g23090 163 bp

MDPMDIVGKSKEDASLPKATMTKIIKEMLPPDVRVARDAQDLLIECCVEFINLVSSESND

VCNKEDKRTIAPEHVLKALQVLGFGEYIEEVYAAYEQHKYETMQDTQRSVKWNPGAQMTE

EEAAAEQQRMFAEARARMNGGVSVPQPEHPETDQRSPQS----

Family: DNC

>DNC_Sc_BE493868/1-60 14 bp

FLPNVRSGGWSDIG

>DNC_Ta1_AL822602/1-60 14 bp

FLPNVRSGGWSDIG

>DNC_Hv_CA019985/1-60 14 bp

FLPSVRSGGWSDIG

>DNC_Hv_BF258447/1-60 14 bp

FVPTIRSGDWSDIG

>DNC_Mt_BG452220/1-93 14 bp

FVPTLRSGXWSDIG

>DNC_Ca_BM064889/1-69 14 bp

FLPTLRSGDWSDIG

>DNC_Nt_AF520810/1-94 14 bp

FLPTLRSGEWSDIG

>DNC_Le_BG133234/1-94 14 bp

FLPVLRSGDWSDIG

>DNC_St_BG351439/1-94 14 bp

FLPVLRSGDWSDIG

>DNC_Ta2_BQ608630/1-60 14 bp

FVPAIRSGDWSDIG

>DNC_Os_CB654058/1-62 14 bp

FVPVIRSGDWSDIG

>DNC_Sp_BG560423/1-62 14 bp

FVPVIRSGDWSDIG

>DNC_In_BJ554393/1-89 14 bp

FLPALRSGEWSDIG

>DNC_Vv_CF211377/1-65 14 bp

FVPALRSGEWSDIG

>DNC_Mc1_CA838286/1-110 14 bp

FLPILRSGEWSDIG

>DNC_Sb_CF071658/1-61 14 bp

FVPFIRSGDWSDIG

>DNC_Os_AP003019/1-73 14 bp

FVPNIRSGDWSDIG

>DNC_Ls_BU003887/1-70 14 bp

FTPMLRSGEWSDIG

Family: G2-Like

>G2-Like_AT-GPRI1 A new protein sequence entered manually 868 bp

M-----------------------------------------------------------

--------------LALSPATRDGCD--------GASEFLDTSCGFTIINPEEEEEFPDF

A----DHGDLLDIID-FDD--I------F---GVAGDVL---------------------

-------------------------------------------------------PDLEI

DP-EILSGDFSNHMNASSTITT--------------------------------------

----------TSDKTDSQGETTKGSSGKGEE--VV---------------SKRDDVAAET

VTYDGDSDRKRKYSSSASSKNNRISNNEGKRKVKVDWTPELHRRFVEAVEQLGVDKAVPS

RILELMGVHCLTRHNVASHLQKYRSHRKHL------------------------------

LAREAEAANW-TRKRHIYGVDTGANL----NGRTKN---------GWLAPAPTLGFPPPP

PVAVAPPPVHHHHF-R-P------------LH-VWGHPT--VDQS----IMPHVW-PKHL

P-PPSTAMPNPPFW---------VSDSP-YWHPMHNG-----------------------

------------------------------------TTP--YLPTVATRFRAPPVAGIPH

ALPPHHTMYK-----PNLGF----GGARPPVDLHPSKESVDAAIGDVLTRPWLPLPLGL-

------------------NPPAV------------------------------------D

GVMTELHRH-GVSEVPPTA---SCA---

>G2-Like_Vv_Predicted A new protein sequence entered manually 868 bp

M-----------------------------------------------------------

--------------LAVSPLRSTNDE--------REGEMESFSIG--------ADDFPDF

Y-----EGNLLECID-FDD--L------FIGIHDG-EIL---------------------

-------------------------------------------------------PDLEM

DP-EI-LAEFSVSAGEESDVNT-------------------SVTEKKVEDNPKKEE----

-------EDKVSDTGSGSGLGYGSSSSRGEE--IV---------------SKRSESVATN

AS-PKEVEKGRK--SSAQSK-----NPQGKRKVKVDWTPELHRRFVQAVEQLGVDKAVPS

RILEIMGIDCLTRHNIASHLQKYRSHRKHL------------------------------

LAREAEAASW-SQKRQMYGVAAGGGG----HKREMG---------GWI--PPTMGFPH--

-----ITPMHHHPF-R-P------------LH-VWGHPT--VDQS----AM-HMW-PKHL

PHSPSPPPPPPPTWPPSATPPS-PSPDP-YWHPHHQR-----------------------

-------------------------VPNGLTQ----GTPCFPQPLAAARFATPPVTGI--

-PP--HPMYKVDHNHPGIGVPARHSGPFPLFDFHPSKETIDAAIGDALSKPWQPLPLGL-

------------------KPPSL------------------------------------D

GVMVELQRQ-GVPKIPPTC---A-----

>G2-Like_Vv_predicted2 A new protein sequence entered manually 868 bp

M-----------------------------------------------------------

--------------TSRTSMKGTCSN----------------------------------

---------------------V--------------------------------------

------------------------------------------------------------

---SI-STIFSSAYTTERYCRT------------------WRWTQKFSLNFPLAPE----

---------------------KNPTSSRGEE--IV---------------SKRSESVATN

AS-PKEVEKGRK--SSAQSK-----NPQGKRKVKVDWTPELHRRFVQAVEQLGVDKAVPS

RILEIMGIDCLTRHNIASHLQKYRSHRKHL------------------------------

LAREAEAASW-SQKRQMYGVAAGGGG----HKREMG---------GWI--PPTMGFPH--

-----ITPMHHHPF-R-P------------LH-VWGHPT--VDQS----AM-HMW-PKHL

PHSPSPPPPPPPTWPPSATPPS-PSPDP-YWHPHHQR-----------------------

-------------------------VPNGLTQ----GTPCFPQPLAAARFATPPVTGI--

-PP--HPMYKVDHNHPGIGVPARHSGPFPLFDFHPSKETIDAAIGDALSKPWQPLPLGL-

------------------KPPSL------------------------------------D

GVMVELQRQ-GVPKIPPTC---A-----

>G2-Like_OS_golden2-like A new protein sequence entered manually 868 bp

M-----------------------------------------------------------

--------------LAVSPA--MCPDIEDRAAVAGDAGME--VVG---MSSDDMDQF-DF

S---------VDDID-FGD--F------FLRLEDG-DVL---------------------

-------------------------------------------------------PDLEV

DPAEI-FTDFEAIATSGGE-GV------------------QDQEVPTVELLAPADDVGVL

DPCGDVVVGEENAAFAGAGEEKGGCNQDDD---AGEANVDDGAAAVE---AKSSSPSSTT

SS-SQEAESRHK----SSSK-----SSHGKKKAKVDWTPELHRRFVQAVEQLGIDKAVPS

RILEIMGIDSLTRHNIASHLQKYRSHRKHM------------------------------

IAREAEAASW-TQRRQIYAAGGGAVA----KRPESN---------AWT--VPTIGFPPPP

PPPPSPAPIQH--FAR-P------------LH-VWGHPT--MDPS----RV-PVWPPRHL

----VPRGPAPPWV-----PPPPPSDPA-FWHHPYMR-----------------------

-------------------------GPA-HVPTQ--GTPCMAMPMPAARFPAPPVPGV--

-VP--CPMYR-PLTPPALASKNQQDA-QLQLQVQPSSESIDAAIGDVLSKPWLPLPLGL-

------------------KPPSV------------------------------------D

SVMGELQRQ-GVANVPPAC---G-----

>G2-Like_OsJ_21268 A new protein sequence entered manually 868 bp

------------------------------------------------------------

----------------------MCPDIEDRAAVAGDAGME--VVG---MSSDDMDQF-DF

S---------VDDID-FGD--F------FLRLEDG-DVL---------------------

-------------------------------------------------------PDLEV

DPAEI-FTDFEAIATSGGE-GV------------------QDQEVPTVELLAPADDVGVL

DPCGDVVVGEENAAFAGAGEEKGGCNQDDD---AGEANVDDGAAAVE---AKSSSPSSTT

SS-SQEAESRHK----SSSK-----SSHGKKKAKVDWTPELHRRFVQAVEQLGIDKAVPS

RILEIMGIDSLTRHNIASHLQKYRSHRKHM------------------------------

IAREAEAASW-TQRRQIYAAGGGAVA----KRPESN---------AWT--VPTIGFPPPP

PPPPSPAPIQH--FAR-P------------LH-VWGHPT--MDPS----RV-PVWPPRHL

----VPRGPAPPWV-----PPPPPSDPA-FWHHPYMR-----------------------

-------------------------GPA-HVPTQ--GTPCMAMPMPAARFPAPPVPGV--

-VP--CPMYR-PLTPPALASKNQQDA-QLQLQVQPSSESIDAAIGDVLSKPWLPLPLGL-

------------------KPPSV------------------------------------D

SVMGELQRQ-GVANVPPAC---G-----

>G2-Like_ZmGLK1 A new protein sequence entered manually 868 bp

M-----------------------------------------------------------

--------------LAVSPSPVRCADAEECGGGGASKEMEETAVG---PVSDSDLDF-DF

T---------VDDID-FGD--F------FLRLDDGDDAL---------------------

-------------------------------------------------------PGLEV

DPAEIVFADFEAIATAGGDGGV------------------TDQEVPSVLPFADAAHIGAV

DPCCGVLGEDNDAACADVEEGKGECDHADEVAAAGNNNSDSGEAGCGGAFAGEKSPSSTA

SS-SQEAESRRK----VSKK-----HSQGKKKAKVDWTPELHRRFVQAVEELGIDKAVPS

RILEIMGIDSLTRHNIASHLQKYRSHRKHM------------------------------

LAREVEAATWTTHRRPMYAAPSGAV-----KRPDSN---------AWT--VPTIGFPPPA

GTPPR--PVQH--FGR-P------------LH-VWGHPS--PTPAVESPRV-PMW-PRHL

----APRAPPPPPW-----APPPPADPASFWHHAYMR-----------------------

-------------------------GPAAHMPDQVAVTPCVAVPMAAARFPAPHVRGS--

-LPWPPPMYR-PLVPPALAGKSQQDA-LFQLQIQPSSESIDAAIGDVLTKPWLPLPLGL-

------------------KPPSV------------------------------------D

SVMGELQRQ-GVANVPQAC---G-----

>G2-Like_OSJNBa0086P08_18 OSJNBa0086P08.18 A new protein sequence entered manually 868 bp

M-----------------------------------------------------------

--------------LEVSTLRSPKADQRAGVGGHHVVGFVPAPPS----PADVADEVDAF

I---VDDSCLLEYID------FSCCDVPFFHADDG-DIL---------------------

-------------------------------------------------------PDLEV

DPTEL-LAEFASSPDDEPPPTTSAPGPGEPAAAAGAKEDVKEDGAAAAAAAAAADYDGSP

PPPRGKKKKDDEERSSSLPEEKDAKNGGGDE--VLSAVTTEDSSAGA---AKSCSPSAEG

HS-KRKPSSSSS--SAAAGK-----NSHGKRKVKVDWTPELHRRFVQAVEQLGIDKAVPS

RILELMGIECLTRHNIASHLQKYRSHRKHL------------------------------

MAREAEAASW-TQKRQMYTAAAAAAAVAAGGGPRKDAAAATAAVAPWV--MPTIGFPPPH

AAAMVPPPPHPPPFCRPP------------LH-VWGHPTAGVEPT----TA-AAP-P---

PPSPHAQPPLLPVWPRHLAPPP-PPLPA-AWAHGHQP-----------------------

-------------------------APVD-------PAAYWQQQYNLQRFPVPPVPGM--

-VP--HPMYR-PIPPPSPPQGNKLAALQLQLDAHPSKESIDAAIGDVLVKPWLPLPLGL-

------------------KPPSL------------------------------------D

SVMSELHKQ-GIPKVPPAA---SGAAG-

>G2-Like_AT_AT5g44190 A new protein sequence entered manually 868 bp

M-----------------------------------------------------------

--------------LTVSPAPVL-------IGNNSKDTYMAADFA-----DFTTEDLPDF

TTVGDFSDDLLDGIDYYDD--L------FIGFD-GDDVL---------------------

-------------------------------------------------------PDLEI

DS-EI-LGEYSGSGRDEEQEME--------------------------------------

--------GNTSTASETSERDVGVCKQEGG---------------------------GGG

DGGFRDKTVRRGKRKGKKSKDCLSDENDIKKKPKVDWTPELHRKFVQAVEQLGVDKAVPS

RILEIMNVKSLTRHNVASHLQKYRSHRKHL------------------------------

LAREAEAASW-NLRRHATVAVPGVG-----GGGKK----------PWT--APALGYPP--

----HVAPMHHGHF-R-P------------LH-VWGHPT---------------W-PKHK

PNTPASAHRTYPMPAIAAAPASWPGHPP-YWHQQ--------------------------

------------------------------------------------------------

------PLY----------------------------------------------PQGY-

------------------------------------------------------------

----------GMAS--------------

>G2-Like_Vv-predicted3 A new protein sequence entered manually 868 bp

MVCTANDLQE------------------WKDFPKGLRVLLLDDDTTSAAEIRSKLEEMDY

IVSTFCNENEALSAISSKPESFHVAIVEVSTGNNGSFKFLET-----------AKDLPTI

MISSIHCLSTMM-------KCIALGAVEFLRKPLSEDKLRNIWQHVVHKAFNAGGSVLPE

SLKPVKESVASMLQLQMENEEPRNESSAETLNVSNVHENDHMQSAGTDKYPAPSTPQLKQ

GGRSLDDGDCLDQTNCSTEKES-------------GEQDGESKSVETTCGTSVAEVTAQV

SPPQGLGE-------SVIKEEDDSADGCKSESNMSPHP------------QNKDSLSEFG

GD-ARNPRKASG--VHSPCG-----TRANRKKMKVDWTPELHKKFVQAVEQLGVDQAIPS

RILELMKVEGLTRHNVASHLQKYRMHRRHI------------------------------

LPKE-DDRRW-PHQRDPMQR-------------------------NYYPQKPVMAFPPYH

SSHTLPAA---------Q------------LYPVWGQPS--SHPA----QMWST--PGYH

TWQPAESWIWKPYP---------GMNAD-AWGCPVM------------------------

-------------------------TPT--------HTPCSSFPQNPSGFDHNNGSGI--

--------YN-------------TGIPQSPIDLYPAEELIDRVVKEAISKPWMPLPLGL-

------------------KPPAT------------------------------------E

SVLAELSRQ-GISTIPPHI---NTPRPY

>G2-Like_Vv-predicted4 A new protein sequence entered manually 868 bp

MVCTANDLQE------------------WKDFPKGLRVLLLDDDTTSAAEIRSKLEEMDY

IVSTFCNENEALSAISSKPESFHVAIVEVSTGNNGSFKFLET-----------AKDLPTI

R-AGMHFLLTISLMDVFDNQMFQLGAVEFLRKPLSEDKLRNIWQHVVHKAFNAGGSVLPE

SLKPVKESVASMLQLQMENEEPRNESSAETLNVSNVHENDHMQSAGTDKYPAPSTPQLKQ

GGRSLDDGDCLDQTNCSTEKES-------------GEQDGESKSVETTCGTSVAEVTAQV

SPPQGLGE-------SVIKEEDDSADGCKSESNMSPHP------------QNKDSLSEFG

GD-ARNPRKASG--VHSPCG-----TRANRKKMKVDWXPELHKKFVQAVEQLGVDQAIPS

RILELMKVEGLTRHNVASHLQKYRMHRRHI------------------------------

LPKE-DDRRW-PHQRDPMQR-------------------------NYYPQKPVMAFPPYH

SSHTLPAA---------Q------------LYPVWGQPS--SHPA----QMWST--PGYH

TWQPAESWIWKPYP---------GMNAD-AWGCPVM------------------------

-------------------------TPT--------HTPCSSFPQNPSGFDHNNGSGI--

--------YN-------------TGIPQSPIDLYPAEEVIDRVVKEAISKPWMPLPLGL-

------------------KPPAT------------------------------------E

SVLAELSRQ-GISTIPPHI---NTPRPY

>G2-Like_C_r-regulator A new protein sequence entered manually 868 bp

MNLGGGQLVKSMAVPSSSASWKSGGDGVSDQFPAGLRVLVVDDDPTCLRILEKMLKNCLY

EVTKCNRAEIALKLLRDNRNGYDIVISDVHMPDMDGFKLLEQVGL--------EMDLPVI

MMSADDSKNVVM-------KGVTHGACDYLIKPVRIEALKNIWQHVVRKGKHEWKE----

-------------------------------------------------------KDYEQ

SG-SVEDGERQQKPNEDVDYSS--------------------------------------

---------------------------------------------------------SAN

EGNWKNSKKRKDDEEDQEEKD----DSSTLKKPRVVWSVELHQQFVAAVNQLGIDKAVPK

KILELMNVPGLTRENVASHLQKYRLYLRRVSGVSQHQNGLNNSFMGTPDATFGSMSSING

LDFQALAATG-QIPAQSLASLQAAAI----GRPTS---------------KPTISMPVVD

QRNLFSFEPHKLRFGEGQQQLNGSTKQISLLH---GIPT-------------NME-PKQL

ASLNQSAQTFGGINMQVSSQASQGNTLL-MQMTQPMSRAPMLNENNASQISRLPSSVGQA

ILSNGIPSGVLGRNGIVDSVRGPVYTPVSQTSSLVDFSVSNSAELPGSTFPHASNSGISS

LTP--KGMMQEEVNSEGKGSRG-----------FPSSYDIFSELQQHKTQDWGLQNVGST

FDGTQHSSIQGSLEGGRNNPPNFGQQFSSYMVDNSLRIKSERHPDANCQTNLFPQLFGQD

DLMSALLKQEGIGSVESDFGFDGYALDL

Family: HRT

>HRT_HRTdb1 30 bp

VCGVMLEDGSSCLEDPMEGRKRCELHKGRR

>HRT_X97909db2 30 bp

ICGIVLEDGTTCTTTPVKGRKRCTEHKGKR

>HRT_Y10013db1 30 bp

ACGVLLEDGTTCTTTPVKXRKRCTEHKGKR

>HRT_Y10013db4 30 bp

ICGFKLYNGSVCEKSPVKGRKRCEEHKGMR

>HRT_X97909db1 30 bp

ICGVILDDGSICSKMPVGKRVRCNEHKGMR

>HRT_Y10013db2 30 bp

ICGVILPDMIRCRSKPVSRRKRCEDHKGMR

>HRT_HRTdb3 30 bp

VCGARASDGSPCKNQPIARRKRCALHKGQR

>HRT_HRTdb2 30 bp

LCGVVTDNG-YCKLEPVIGRERCEEHRGIE

>HRT_Y10013db3 30 bp

LCEATTKNGLPCTRSAPEGSKRCWQHKDKT

Family: LUFS

>LUFS_gi|18418034|ref|NP_567896_1| gi|18418034|ref|NP_567896.1| LUG (LEUNIG); protein binding / protein heterodimerization/ transcription repressor [Arabidopsis thaliana] 1124 bp

MS----------------------------------------------------------

-------QTNWEADKMLDVYIHDYLVKRDLKATAQAFQAEGKVSSD----PV--------

---------AIDAPGGFLFEWWSVFWDIFIA----RTNEKHSEVAASYIETQMIKAREQQ

LQQSQHPQVSQQQQQQQQQQIQMQQLLLQRAQQQQQQQQQQHHHHQQQQQQQQQQQQQQQ

QQQQQHQNQ----PPSQQQQQQSTPQHQQQPTPQQQPQRRDGSHLANGSANGLVGNNS-E

PVMRQNPGSGSSLASKAYEERVKMPTQRESLDEAAMKRFGDNVGQLLDPSHASILKSAAA

SGQPAGQVLHST-SGGMSP---QVQTRNQQLPGSAVDIKSEINPVLTPR-------TAVP

EGSL-----IGIPGSNQGSNNLTLKGWPLTGFDQLRSGL--LQQQKPFMQSQSFHQLNML

TPQHQQQLMLAQQNLNSQSVSEENRRLKMLLNNRSMTLGKDGLGSSVGDVLPNV----GS

SLQPGG-SL----LPRGDTDMLLKLKMALLQQQ-----QQNQQQGGGNPPQPQPQPQPLN

QLALTNPQPQSSNHSIHQQEKLGGGGSITMDGSISNSFRGNEQVLKNQSGRKRKQPVSSS

GPANSSGT--------ANTAGP-SPSSAPSTPSTHT-PGDVISMP--NLPHS-----GG-

-SSKSMMMFGTEG-TGTLTSPSNQLA--DMDRFVEDGSLDDNVESFLS--------QEDG

D---------QRDAVTRCMDVSKG---FTFTEVN------SVRASTTKVTCCHFSSDGKM

L-ASAGHDKKAVLWYTDTMKPKTTLEEHTAMITDIRFSPSQLRLATSSFDKTVRVWDADN

KGYSLRTFMGHSSMV-----------TSLDFHPIKDDLICSC---DNDNEIRYWSINNGS

CTRVYKGGSTQIRFQPRVGKYLAASSANLVNVLDVETQAIRH--SL-QGHANPINSVCWD

PSGDFLASVSEDMVKVWTLGTGSEGECVHELSCNGNKFQSCVFHPAYPSLLVIGCYQSLE

LWNMSENKTMTLPAHEGLITSLAVSTATGLVASASHDKLVKLWK

>LUFS_gi|2914703|gb|AAC04493_1| gi|2914703|gb|AAC04493.1| expressed protein [Arabidopsis thaliana] 1124 bp

MA----------------------------------------------------------

-------QSNWEADKMLDVYIYDYLVKKKLHNTAKSFMTEGKVSPD----PV--------

---------AIDAPGGFLFEWWSVFWDIFIA----RTNEKHSEAAAAYIEAQQGKAKEQQ

M--------------------QIQQLQMMR------------------------------

---------------------------------QAQMQRRDPNHPSLGGPMNAIG--S-E

GMIGQS--NASALAAKMYEERMKQPNPMNS-----------ETSQPHLDARMALLKSAT-

--NHHGQIVQGNHQGGVSAALQQIQSRTQQ----PTEIKTEVNLGTSPRQLPVDPSTVYG

QGILQSKPGMGSAGLNPGVSGLPLKGWPLTGIEQMRPGLGGPQVQKSFLQNQSQFQ---L

SPQQQQHQMLAQVQAQGNMTNS-----------------------------PMY----GG

DMDPRRFTG----LPRGNLN---------PKDG-----QQNANDGSIGSPMQSSSSKHIS

MPPVQQSSSQQQDHLLSQQSQ--------------------------QNNRKRKGP-SSS

GPANSTGT--------GNTVGP-S-NSQPSTPSTHT-PVDGVAIAG-NMHHV-----NS-

-MPKGPMMYGSDG-IGGLASSANQLLQDDMDQFGDVGALEDNVESFLS--------QDDG

D-GGSLFGTLKRNSSVH-TETSKP---FSFNEVS------CIRKSASKVICCSFSYDGKL

L-ASAGHDKKVFIWNMETLQVESTPEEHAHIITDVRFRPNSTQLATSSFDKTIKIWDASD

PGYFLRTISGHAAPV-----------MSIDFHPKKTELLCSC---DSNNDIRFWDI-NAS

CVRAVKGASTQVRFQPRTGQFLAAASENTVSIFDIENNNKRV--NIFKGHSSNVHSVCWS

PNGELVASVSEDAVKLWSL---SSGDCIHELSNSGNKFHSVVFHPSYPDLLVIGGYQAIE

LWNTMENKCMTVAGHECVISALAQSPSTGVVASASHDKSVKIWK

>LUFS_gi|2506904|sp|P40068_2|FLO8_YEAST gi|2506904|sp|P40068.2|FLO8_YEAST RecName: Full=Transcriptional activator FLO8; AltName: Full=Protein PDH5 1124 bp

MSYKVNSSYPDSIPPTEQPYMASQYKQDLQSNIAMATNSEQQRQQQQQQQQQQQQWINQP

TAENSDLKEKMNCKNTLNEYIFDFLTKSSLKNTAAAFAQDAHLDRDKGQNPVDGPKSKEN

NGNQNTFSKVVDTPQGFLYEWWQIFWDIFNT----SSSRGGSEFAQQYY-----------

------------------------QLVLQEQRQEQIYRSLAVHAARLQHDAERRGEYSNE

DIDPMHLAAMMLGNPMAPAVQMRNVNMNPIPIPMV--------------GNPIVNNFSIP

PYNNANPTTGAT--------------------------------------------AVAP

TAPPSGDFTN------VGP----TQNRSQNVTGWPVY------------NYPMQPTTENP

---------VGNPCNNNTTNNTT-------------------NNKSPVNQPKSLKTMHST

DKPNNVPTSKSTRSRSATSKAKGKVKAGLVAKRRRKNNTATVSAGSTNACSPNITTPGST

TSEPAMVGSRVNKTPRSDIATNFRNQAIIFGEEDIYSNSKSSPSLDGASPSALASKQP-T

KVRKNTKKASTSAFPVESTNKLGGNSVVT----------GKKRSPPNTRVSRRKSTPSVI

LNADATKDENNMLRTFSNTIAP-NIHSAPPTKTANSLPFPGINLGSFNKPAV-----SSP

LSSVTESCFDPES--GKIAGKN-----------GPKRAVNSKVSASSPLSIATPRSGDAQ

K---------QRSSKVPGNVVIKPPHGFSTTNLNITLKNSKIITSQNNTVSQELPNGGNI

LEAQVGNDSRSSKGNRNTL---STPEEKKP--------------------------SSNN

QGYDFDALKNSSSLLFPNQAYASNNRTPNENSNVADETSASTNSGDNDNTLIQPSSNVGT

TLGPQQTSTNE-------NQNVHSQNLKFGNIGMVEDQGPDYDLNLLDTNENDFNFINWE

------------------------------------------------------------

-------------------------------------------G

>LUFS_gi|4689144|gb|AAD27781_1|AF077048_1 gi|4689144|gb|AAD27781.1|AF077048_1 single strand DNA-binding protein [Homo sapiens] 1124 bp

MYGK-------------------------------------------------------G

KSNSSAVPSDSQAREKLALYVYEYLLHVGAQKSAQTFLSEIRWEKN-----I--------

---------TLGEPPGFLHSWWCVFWDLYCAAPERRETCEHSSEAKAFHDYSAAAA----

------------------------------------------------------------

-------------------------------------------------PSPVLGNIP--

------PGDG--------------------------------------------------

--MPVGPVPPGFFQPFMSP-------RYPGGPRPPLRIPNQA-LGGVPGSQPLLPSGMDP

------------------------------TRQQGHPNMGGPMQR---------------

NDSSKRNGCLRTTVLTPGLSLQ------------------------------NY----GG

AMRPPLNALGGPGMPGMNMG------------------------PGGGRPWPNPTN----

---------------------------------------------------------ANS

IPYSSASP--------GNYVGPPGGGGPPGTPIMPS-PADSTNSGD-NMYTLMNAVPPGP

NRPNFPMGPGSDGPMGGLGGMESHHMNGSLGS-GDMDSISKNSPNNMSLSNQPGTPRDDG

EMGGNFLNPFQSESYSPSMTMS--------------------------------------

------------------------------------------------------------

------------------------------------------------------------

------------------------------------------------------------

------------------------------------------------------------

-------------------------------------------V

Family: NF-YB

>NF-YB_DPOE3_MOUSE#4#72 | 69 | Q9JKP7; | PD002982 171 bp

--------RPEDLNLPN--AV---------ITRIIK-------------------EAL--

--------------------PD----------------------------G--VNISKEA

RSAIS--------RAASVFVLYATSCANNFAMK--GK--RKTLNASDVLSA

>NF-YB_Q3SZN5_BOVIN#1#72 | 72 | Q3SZN5; | PD002982 171 bp

-----MAERPEDLNLPN--AV---------ITRIIK-------------------EAL--

--------------------PD----------------------------G--VNISKEA

RSAIS--------RAASVFVLYATSCANNFAMK--GK--RKTLNASDVLSA

>NF-YB_DPOE3_PONPY#1#72 | 72 | Q5R4W3; | PD002982 171 bp

-----MAERPEDLNLPN--AV---------ITRIIK-------------------EAL--

--------------------PD----------------------------G--VNISKEA

RSAIS--------RAASVFVLYATSCANNFAMK--GK--RKTLNASDVLSA

>NF-YB_DPOE3_HUMAN#1#72 | 72 | Q9NRF9; | PD002982 171 bp

-----MAERPEDLNLPN--AV---------ITRIIK-------------------EAL--

--------------------PD----------------------------G--VNISKEA

RSAIS--------RAASVFVLYATSCANNFAMK--GK--RKTLNASDVLSA

>NF-YB_DPOE3_RAT#1#72 | 72 | Q642A5; | PD002982 171 bp

-----MAERPEDLNLPN--AV---------ITRIIK-------------------EAL--

--------------------PD----------------------------G--VNISKEA

RSAIS--------RAASVFVLYATSCANNFAMK--GK--RKTLNASDVLSA

>NF-YB_Q6WSQ3_XENLA#1#72 | 72 | Q6WSQ3; | PD002982 171 bp

-----MAERPEDLNLPN--AV---------VTRIIK-------------------EAL--

--------------------PE----------------------------G--VNISKEA

RSAIS--------RAASVFVLYATSCANNFAMK--GK--RKTLNASDVLAA

>NF-YB_Q4T8I6_TETNG#1#72 | 72 | Q4T8I6; | PD002982 171 bp

-----MAERPEDLNLPN--AV---------ITRIIK-------------------EAL--

--------------------PD----------------------------G--VNVSKEA

RRAIS--------QAASVFVLYATSCANNFAMK--AK--RKTLNAGDVLAA

>NF-YB_Q6PBL4_BRARE#1#72 | 72 | Q6PBL4; | PD002982 171 bp

-----MAERPEDLNLPN--AV---------ITRIIK-------------------EAL--

--------------------PE----------------------------G--VNVSKEA

RRAIS--------QAASVFVLYATSCANSFAMK--AK--RKTLNAGDVMSA

>NF-YB_Q5DFV9_SCHJA#1#72 | 72 | Q5DFV9; | PD002982 171 bp

-----MAEKAEDLYLPN--AV---------LLRIIR-------------------ESL--

--------------------PE----------------------------R--TLVSREA

RSAIS--------KSASSFILYVTSLASVHSEK--AK--RKTLTGNDILAA

>NF-YB_Q86F18_SCHJA#1#72 | 72 | Q86F18; | PD002982 171 bp

-----MAEKAEDLYLPN--AV---------LLRIIR-------------------ESL--

--------------------PE----------------------------R--TLVSREA

RSAIS--------KSASSFILYVTSLASVHSEK--AK--RKTLTGNDILAA

>NF-YB_Q9V444_DROME#1#70 | 70 | Q9V444; | PD002982 171 bp

-----MVERIEDLNLPN--AV---------IGRLIK-------------------EAL--

--------------------PE----------------------------S--ASVSKEA

RAAIA--------RAASVFAIFVTSSSTALAHK--QN--HKTITAKDIL--

>NF-YB_Q22798_CAEEL#19#76 | 58 | Q22798; | PD002982 171 bp

---------------PA--AI---------VTRLMK-------------------E----

---------------------D----------------------------N--ISASKDA

RDVIA--------RAAAVFLINLSDVSAQSARD--QK--HKTISGDDVVK-

>NF-YB_Q60V65_CAEBR#18#76 | 59 | Q60V65; | PD002982 171 bp

--------------LPA--AI---------VTRLMK-------------------E----

---------------------E----------------------------N--ISGSKEA

RDLIT--------RAAAVFLINLSDVAVQAARE--VK--QKTLSAEHVLK-

>NF-YB_Q5BE05_EMENI#37#101 | 65 | Q5BE05; | PD002982 171 bp

---------IEDYLLPR--SI---------TLRLAK-------------------SVL--

--------------------PP----------------------------N--TSVQKDA

VLAIQ--------KAATVFVSYLSSHANEATL-------KRTVSPADVLNA

>NF-YB_Q4WT49_ASPFU#53#116 | 64 | Q4WT49; | PD002982 171 bp

----------QDYLLPR--SL---------TLRLAK-------------------AVL--

--------------------PP----------------------------N--TAIQKDA

VLAIQ--------KAATVFVSYLASHANEATL-------KRTVAPADVFSA

>NF-YB_Q7SDT7_NEUCR#129#195 | 67 | Q7SDT7; | PD002982 171 bp

----------EDLTLPK--SI---------ITRLAK-------------------GVL--

--------------------PS----------------------------N--TQIQANA

ILAMT--------KSATVFISHLANAANEHTVS--SG--KKTIMPADVFKA

>NF-YB_Q4INM6_GIBZE#89#155 | 67 | Q4INM6; | PD002982 171 bp

----------EDLTLPK--SI---------ITRLSK-------------------GVL--

--------------------PP----------------------------N--TQIQANA

IMALS--------QSTTVFINYLASHANENTVN--AG--KKTIAPADVFKA

>NF-YB_DPB4_SCHPO#13#79 | 67 | P87174; | PD002982 171 bp

----------DDLALPR--SI---------IMRLVK-------------------GVL--

--------------------PE----------------------------K--SLVQKEA

LKAMI--------NSATLFVSFLTSASGEIATN--NN--RKILMPQDVLNA

>NF-YB_DPB4_YARLI#33#99 | 67 | Q6CHS6; | PD002982 171 bp

----------DDLLYPK--ST---------IKNLAK-------------------ETL--

--------------------PD----------------------------D--AIISKDA

LTAIQ--------RAATLFVSYMASHGNASAEA--GG--RKKITPQDVFVA

>NF-YB_Q4P9C5_USTMA#81#143 | 63 | Q4P9C5; | PD002982 171 bp

--------------LPR--GP---------LIKLAK-------------------SSL--

--------------------PD----------------------------N--VQLRRDV

QFALV--------RSASVFISYLTATAHDTARR--KK--KKNILPEHVMEA

>NF-YB_Q5K7U8_CRYNE#6#67 | 62 | Q5K7U8; | PD002982 171 bp

--------------LPK--TT---------LTKLAK-------------------GSI--

--------------------PD----------------------------N--VKMQQDV

VLALL--------RGSTLFISYLSP-AHDQAIA--RS--GRTVTAADVIKA

>NF-YB_Q55IB2_CRYNE#6#67 | 62 | Q55IB2; | PD002982 171 bp

--------------LPK--TT---------LTKLAK-------------------GSI--

--------------------PD----------------------------N--VKMQQDV

VLALL--------RGSTLFISYLSP-AHDQAIA--RS--GRTVTAADVIKA

>NF-YB_DPB4_DEBHA#32#100 | 69 | Q6BIP4; | PD002982 171 bp

---------------PR--AT---------VQKLAK-------------------NIMNA

SSDE---------------GAS----------------------------N--MILAKDS

MIALQ--------RSSTVFVSHLMFQARQISKD--EG--RKTINAQDILSA

>NF-YB_Q5ACX9_CANAL#27#100 | 74 | Q5ACX9; | PD002982 171 bp

---------IDDILFPR--TT---------IQKLAK-------------------NII--

SDDE--------------NNAG----------------------------Q--MTIAKDS

LLALQ--------RSATVFVSHLLFQAKQISKE--AN--RKTVSAQDMLAA

>NF-YB_DPB4_CANGA#29#117 | 89 | Q6FXD0; | PD002982 171 bp

----------QDLLFPR--SV---------IMALAK-------------------EVPEM

QQQQVQVQAAEKGEPVEKTPAK----------------------------K--LVVTKDA

SMALQ--------HSATVFVNHLLMYARELAKE--QD--RRSCNVDDILNA

>NF-YB_DPB4_YEAST#28#98 | 71 | Q04603; | PD002982 171 bp

---------IQDLLFPK--ST---------IVNLAR-------------------EVP--

-QQS----------------GK----------------------------K--LLINKDA

SLALQ--------RGATVFVNHLLLFAREIAKS--QD--KKSCSVDDVLSA

>NF-YB_DPB4_KLULA#28#104 | 77 | Q6CJD7; | PD002982 171 bp

---------IDDLLFPK--SI---------ITSLAK-------------------ESL--

-QNAFQKGEEE----------R----------------------------R--ITVSKDA

ALAMQ--------RSATVFVNHLLMFARMNAKD--SN--RKSCNDQDIMAA

>NF-YB_DPB4_ASHGO#68#143 | 76 | Q750A4; | PD002982 171 bp

----------DDLLFPR--SI---------ITSLAK-------------------DAV--

-HQAVQTAEQD----------P----------------------------R--VMLSKDA

SLALQ--------RSSTVFVNHLLMHARQIAQS--ND--RKSCSGEDVLKA

>NF-YB_NFYBA_ARATH#28#96 | 69 | Q67XJ2; | PD002982 171 bp

--------REQDRFLPI--AN---------ISRIMK-------------------RGL--

--------------------PL----------------------------N--GKIAKDA

KETMQ--------ECVSEFISFVTSEASDKCQR--EK--RKTINGDDLLWA

>NF-YB_NFYB8_ARATH#29#97 | 69 | Q8VYK4; | PD002982 171 bp

--------REQDRFLPI--AN---------ISRIMK-------------------RGL--

--------------------PA----------------------------N--GKIAKDA

KEIVQ--------ECVSEFISFVTSEASDKCQR--EK--RKTINGDDLLWA

>NF-YB_NFYB3_ORYSA#37#105 | 69 | Q60EQ4; | PD002982 171 bp

--------REQDRFLPI--AN---------ISRIMK-------------------KAI--

--------------------PA----------------------------N--GKIAKDA

KETVQ--------ECVSEFISFITSEASDKCQR--EK--RKTINGDDLLWA

>NF-YB_NFYB_MAIZE#30#98 | 69 | P25209; | PD002982 171 bp

--------REQDRFLPI--AN---------ISRIMK-------------------KAI--

--------------------PA----------------------------N--GKIAKDA

KETVQ--------ECVSEFISFITSEASDKCQR--EK--RKTINGDDLLWA

>NF-YB_Q75IZ7_ORYSA#23#91 | 69 | Q75IZ7; | PD002982 171 bp

--------REQDRFLPI--AN---------VSRIMK-------------------KAL--

--------------------PA----------------------------N--AKISKDA

KETVQ--------ECVSEFISFITGEASDKCQR--EK--RKTINGDDLLWA

>NF-YB_Q6YNK1_ORYSA#23#91 | 69 | Q6YNK1; | PD002982 171 bp

--------REQDRFLPI--AN---------VSRIMK-------------------KAL--

--------------------PA----------------------------N--AKISKDA

KETVQ--------ECVSEFISFITGEASDKCQR--EK--RKTINGDDLLWA

>NF-YB_NFYB3_ARATH#20#88 | 69 | O23310; | PD002982 171 bp

--------REQDRFLPI--AN---------VSRIMK-------------------KAL--

--------------------PA----------------------------N--AKISKDA

KETVQ--------ECVSEFISFITGEASDKCQR--EK--RKTINGDDLLWA

>NF-YB_Q69J40_ORYSA#21#89 | 69 | Q69J40; | PD002982 171 bp

--------REQDRFLPI--AN---------VSRIMK-------------------RAL--

--------------------PA----------------------------N--AKISKDA

KETVQ--------ECVSEFISFITGEASDKCQR--EK--RKTINGDDLLWA

>NF-YB_Q6YNK0_ORYSA#57#125 | 69 | Q6YNK0; | PD002982 171 bp

--------KEQDRFLPI--AN---------VSRIMK-------------------RSL--

--------------------PA----------------------------N--AKISKEA

KETVQ--------ECVSEFISFVTGEASDKCQR--EK--RKTINGDDLLWA

>NF-YB_NFYB2_ARATH#23#94 | 72 | Q9FGJ3; | PD002982 171 bp

-----LSPREQDRFLPI--AN---------VSRIMK-------------------KAL--

--------------------PA----------------------------N--AKISKDA

KETMQ--------ECVSEFISFVTGEASDKCQK--EK--RKTINGDDLLWA

>NF-YB_Q7E0Y8_ORYSA#19#87 | 69 | Q7E0Y8; | PD002982 171 bp

--------REQDRFLPI--AN---------ISRIMK-------------------KAV--

--------------------PA----------------------------N--GKIAKDA

KETLQ--------ECVSEFISFVTSEASDKCQK--EK--RKTINGEDLLFA

>NF-YB_NFYB2_ORYSA#33#101 | 69 | Q5QMG3; | PD002982 171 bp

--------REQDRFLPI--AN---------ISRIMK-------------------KAV--

--------------------PA----------------------------N--GKIAKDA

KETLQ--------ECVSEFISFVTSEASDKCQK--EK--RKTINGEDLLFA

>NF-YB_Q2XTB9_SOLTU#27#95 | 69 | Q2XTB9; | PD002982 171 bp

--------REQDRYLPI--AN---------IGRIMK-------------------KAL--

--------------------PA----------------------------N--GKIAKDS

KDTVQ--------ECVSEFISFITSEASDKCQK--EK--RKTINGDDLLSA

>NF-YB_NFYB1_ARATH#20#88 | 69 | Q9SLG0; | PD002982 171 bp

--------REQDRYLPI--AN---------ISRIMK-------------------KAL--

--------------------PP----------------------------N--GKIGKDA

KDTVQ--------ECVSEFISFITSEASDKCQK--EK--RKTVNGDDLLWA

>NF-YB_Q3EBK1_ARATH#20#88 | 69 | Q3EBK1; | PD002982 171 bp

--------REQDRYLPI--AN---------ISRIMK-------------------KAL--

--------------------PP----------------------------N--GKIGKDA

KDTVQ--------ECVSEFISFITSEASDKCQK--EK--RKTVNGDDLLWA

>NF-YB_NFYB7_ARATH#35#103 | 69 | Q9SIT9; | PD002982 171 bp

--------KEQDRFLPI--AN---------VGRIMK-------------------KVL--

--------------------PG----------------------------N--GKISKDA

KETVQ--------ECVSEFISFVTGEASDKCQR--EK--RKTINGDDIIWA

>NF-YB_NFYB5_ARATH#47#118 | 72 | O82248; | PD002982 171 bp

-----MMVKEQDRLLPI--AN---------VGRIMK-------------------NIL--

--------------------PA----------------------------N--AKVSKEA

KETMQ--------ECVSEFISFVTGEASDKCHK--EK--RKTVNGDDICWA

>NF-YB_Q942Y5_ORYSA#33#101 | 69 | Q942Y5; | PD002982 171 bp

--------KEQDRLLPI--AN---------VGRIMK-------------------QIL--

--------------------PP----------------------------N--AKISKEA

KETMQ--------ECVSEFISFVTGEASDKCHK--EK--RKTVNGDDVCWA

>NF-YB_NFYB4_ORYSA#21#88 | 68 | Q65XK1; | PD002982 171 bp

--------KEQDRFLPI--AN---------IGRIMR-------------------RAV--

--------------------PE----------------------------N--GKIAKDS

KESVQ--------ECVSEFISFITSEASDKCLK--EK--RKTINGDDLIW-

>NF-YB_Q4WK36_ASPFU#40#117 | 78 | Q4WK36; | PD002982 171 bp

--------KEQDRWLPI--ANATCANIYRQVARIMK-------------------LAL--

--------------------PE----------------------------N--AKIAKEA

KECMQ--------ECVSEFISFITSEASEKCQQ--EK--RKTVNGEDILFA

>NF-YB_Q96WP0_TRIRE#45#113 | 69 | Q96WP0; | PD002982 171 bp

--------KEQDRWLPI--AN---------VARIMK-------------------NAL--

--------------------PD----------------------------N--AKIAKEA

KECMQ--------ECVSEFISFITSEASEKCQQ--EK--RKTVNGEDILFA

>NF-YB_Q6MVJ9_NEUCR#44#112 | 69 | Q6MVJ9; | PD002982 171 bp

--------KEQDRWLPI--AN---------VARIMK-------------------NAL--

--------------------PE----------------------------N--AKIAKEA

KECMQ--------ECVSEFISFITSEASEKCQQ--EK--RKTVNGEDILFA

>NF-YB_O59848_ASPOR#42#110 | 69 | O59848; | PD002982 171 bp

--------KEQDRWLPI--AN---------VARIMK-------------------LAL--

--------------------PD----------------------------N--AKIAKEA

KECMQ--------ECVSEFISFITSEASEKCQQ--EK--RKTVNGEDILFA

>NF-YB_Q5B5Z6_EMENI#42#110 | 69 | Q5B5Z6; | PD002982 171 bp

--------KEQDRWLPI--AN---------VARIMK-------------------LAL--

--------------------PE----------------------------N--AKIAKEA

KECMQ--------ECVSEFISFITSEASEKCQQ--EK--RKTVNGEDILFA

>NF-YB_Q86ZV5_ASPNG#43#111 | 69 | Q86ZV5; | PD002982 171 bp

--------KEQDRWLPI--AN---------VARIMK-------------------LAL--

--------------------PE----------------------------N--AKIAKEA

KECMQ--------ECVSEFISFITSEASEKCQQ--EK--RKTVNGEDILFA

>NF-YB_Q00735_EMENI#42#110 | 69 | Q00735; | PD002982 171 bp

--------KEQDRWLPI--AN---------VARIMK-------------------LAL--

--------------------PE----------------------------N--AKIAKEA

KECMQ--------ECVSEFISFITSEASEKCQQ--EK--RKTVNGEDILFA

>NF-YB_Q6C1E2_YARLI#17#85 | 69 | Q6C1E2; | PD002982 171 bp

--------REQDKWLPI--AN---------VARIMK-------------------SAL--

--------------------PE----------------------------N--AKVSKEA

KECMQ--------ECVSEFISFITSEASEKCAA--EK--RKTVNGEDILFA

>NF-YB_Q55S56_CRYNE#41#109 | 69 | Q55S56; | PD002982 171 bp

--------REQDRWLPI--AN---------VARIMK-------------------SSL--

--------------------PT----------------------------S--AKVSKEA

KECVQ--------ECVSEFISFITSEAAEKCLN--EK--RKTLNGEDILTS

>NF-YB_Q5KGK8_CRYNE#41#109 | 69 | Q5KGK8; | PD002982 171 bp

--------REQDRWLPI--AN---------VARIMK-------------------SSL--

--------------------PT----------------------------S--AKVSKEA

KECVQ--------ECVSEFISFITSEAAEKCLN--EK--RKTLNGEDILTS

>NF-YB_Q8C590_MOUSE#53#121 | 69 | Q8C590; | PD002982 171 bp

--------REQDIYLPI--AN---------VARIMK-------------------NAI--

--------------------PQ----------------------------T--GKIAKDA

KECVQ--------ECVSEFISFITSEASERCHQ--EK--RKTINGEDILFA

>NF-YB_Q5FVT0_RAT#53#121 | 69 | Q5FVT0; | PD002982 171 bp

--------REQDIYLPI--AN---------VARIMK-------------------NAI--

--------------------PQ----------------------------T--GKIAKDA

KECVQ--------ECVSEFISFITSEASERCHQ--EK--RKTINGEDILFA

>NF-YB_Q3UK54_MOUSE#53#121 | 69 | Q3UK54; | PD002982 171 bp

--------REQDIYLPI--AN---------VARIMK-------------------NAI--

--------------------PQ----------------------------T--GKIAKDA

KECVQ--------ECVSEFISFITSEASERCHQ--EK--RKTINGEDILFA

>NF-YB_Q32KW0_BOVIN#53#121 | 69 | Q32KW0; | PD002982 171 bp

--------REQDIYLPI--AN---------VARIMK-------------------NAI--

--------------------PQ----------------------------T--GKIAKDA

KECVQ--------ECVSEFISFITSEASERCHQ--EK--RKTINGEDILFA

>NF-YB_NFYB_RAT#53#121 | 69 | P63140; | PD002982 171 bp

--------REQDIYLPI--AN---------VARIMK-------------------NAI--

--------------------PQ----------------------------T--GKIAKDA

KECVQ--------ECVSEFISFITSEASERCHQ--EK--RKTINGEDILFA

>NF-YB_NFYB_MOUSE#53#121 | 69 | P63139; | PD002982 171 bp

--------REQDIYLPI--AN---------VARIMK-------------------NAI--

--------------------PQ----------------------------T--GKIAKDA

KECVQ--------ECVSEFISFITSEASERCHQ--EK--RKTINGEDILFA

>NF-YB_NFYB_HUMAN#53#121 | 69 | P25208; | PD002982 171 bp

--------REQDIYLPI--AN---------VARIMK-------------------NAI--

--------------------PQ----------------------------T--GKIAKDA

KECVQ--------ECVSEFISFITSEASERCHQ--EK--RKTINGEDILFA

>NF-YB_NFYB_HORSE#53#121 | 69 | Q6RG77; | PD002982 171 bp

--------REQDIYLPI--AN---------VARIMK-------------------NAI--

--------------------PQ----------------------------T--GKIAKDA

KECVQ--------ECVSEFISFITSEASERCHQ--EK--RKTINGEDILFA

>NF-YB_Q6T2F0_BUFBG#52#120 | 69 | Q6T2F0; | PD002982 171 bp

--------REQDIYLPI--AN---------VARIMK-------------------NAI--

--------------------PQ----------------------------T--GKIAKDA

KECVQ--------ECVSEFISFITSEASERCHQ--EK--RKTINGEDILFA

>NF-YB_Q5CZV3_BRARE#52#120 | 69 | Q5CZV3; | PD002982 171 bp

--------REQDIYLPI--AN---------VARIMK-------------------NAI--

--------------------PQ----------------------------T--GKIAKDA

KECVQ--------ECVSEFISFITSEASERCHQ--EK--RKTINGEDILFA

>NF-YB_NFYB_CHICK#51#119 | 69 | P25207; | PD002982 171 bp

--------REQDIYLPI--AN---------VARIMK-------------------NAI--

--------------------PQ----------------------------T--GKIAKDA

KECVQ--------ECVSEFISFITSEASERCHQ--EK--RKTINGEDILFA

>NF-YB_Q3U1S7_MOUSE#53#121 | 69 | Q3U1S7; | PD002982 171 bp

--------REQDIYLPI--AN---------VARIMK-------------------NAI--

--------------------PQ----------------------------T--GKIAKDA

KECVQ--------ECVSEFISFITSEASERCHQ--EK--RKTINGEDILFA

>NF-YB_Q9D056_MOUSE#15#83 | 69 | Q9D056; | PD002982 171 bp

--------REQDIYLPI--AN---------VARIMK-------------------NAI--

--------------------PQ----------------------------T--GKIAKDA

KECVQ--------ECVSEFISFITSEASERCHQ--EK--RKTINGEDILFA

>NF-YB_Q63091_RAT#15#83 | 69 | Q63091; | PD002982 171 bp

--------REQDIYLPI--AN---------VARIMK-------------------NAI--

--------------------PQ----------------------------T--GKIAKDA

KECVQ--------ECVSEFISFITSEASERCHQ--EK--RKTINGEDILFA

>NF-YB_Q6DD00_XENLA#52#120 | 69 | Q6DD00; | PD002982 171 bp

--------REQDIYLPI--AN---------VARIMK-------------------NAV--

--------------------PQ----------------------------T--GKIAKDA

KECVQ--------ECVSEFISFITSEASERCHQ--EK--RKTINGEDILFA

>NF-YB_O73744_XENLA#52#120 | 69 | O73744; | PD002982 171 bp

--------REQDIYLPI--AN---------VARIMK-------------------NAV--

--------------------PQ----------------------------T--GKIAKDA

KECVQ--------ECVSEFISFITSEASERCHQ--EK--RKTINGEDILFA

>NF-YB_Q561X4_BRARE#51#119 | 69 | Q561X4; | PD002982 171 bp

--------REQDIYLPI--AN---------VARIMK-------------------NAV--

--------------------PQ----------------------------T--GKIAKDA

KECVQ--------ECVSEFISFITSEASERCHQ--EK--RKTINGEDILFA

>NF-YB_NFYB_PETMA#54#122 | 69 | P25210; | PD002982 171 bp

--------REQDIYLPI--AN---------VARIMK-------------------TSI--

--------------------PS----------------------------S--GKIAKDA

KECVQ--------ECVSEFISFITSEASERCHQ--EK--RKTINGEDILFA

>NF-YB_Q4H329_CIOIN#34#102 | 69 | Q4H329; | PD002982 171 bp

--------REQDIYLPI--AN---------VARIMK-------------------NAV--

--------------------PS----------------------------N--GKIAKDA

KECVQ--------ECVSEFISFITSEASERCAQ--EK--RKTINGEDLLFA

>NF-YB_Q6T2F1_BUFBG#52#148 | 97 | Q6T2F1; | PD002982 171 bp

--------REQDIYLPI--AN---------VARIMK-------------------NAI--

--------------------PHRKSNVYRFHFFLPTVQPHFTPSVNTMLLA--EEIAKDA

KECVQ--------ECVSEFISFITSEASERCHQ--EK--RKTINGEDILFA

>NF-YB_Q8WQQ1_STRPU#54#122 | 69 | Q8WQQ1; | PD002982 171 bp

--------REQDRFLPI--AN---------VARIMK-------------------DGI--

--------------------PK----------------------------S--GKISKEA

KECVQ--------ECVSEFISFITSEASERCHQ--EK--RKTINGEDILYA

>NF-YB_O76256_SCHMA#23#91 | 69 | O76256; | PD002982 171 bp

--------REQDRFLPI--AN---------VAKIMK-------------------RAV--

--------------------PG----------------------------N--GKIAKDA

KECVQ--------ECVSEFISFITSELPDKCQT--EK--RKTINGEDILCA

>NF-YB_Q7PN95_ANOGA#26#94 | 69 | Q7PN95; | PD002982 171 bp

--------REQDRFLPI--AN---------ITKIMK-------------------KSV--

--------------------PN----------------------------N--GKIAKEA

RECIQ--------ECVSEFISFITSEASDRCHM--EK--RKTINGEDILCA

>NF-YB_Q754Q8_ASHGO#19#85 | 67 | Q754Q8; | PD002982 171 bp

--------REQDRWLPI--NN---------VARLMK-------------------NTL--

--------------------PV----------------------------T--TKVSKDA

KECMQ--------ECVSEFISFVTSEASDRCAS--DK--RKTINGEDVL--

>NF-YB_HAP3_YEAST#36#102 | 67 | P13434; | PD002982 171 bp

--------REQDRWLPI--NN---------VARLMK-------------------NTL--

--------------------PP----------------------------S--AKVSKDA

KECMQ--------ECVSELISFVTSEASDRCAA--DK--RKTINGEDIL--

>NF-YB_Q6FPE7_CANGA#16#82 | 67 | Q6FPE7; | PD002982 171 bp

--------REQDRWLPI--NN---------VARLMK-------------------NTL--

--------------------PE----------------------------T--AKVSKSA

KECMQ--------ECVSEFISFVTSEASDRCAQ--DR--RKTINGEDIL--

>NF-YB_Q5A6N7_CANAL#12#78 | 67 | Q5A6N7; | PD002982 171 bp

--------REQDRWLPI--AN---------VARIMK-------------------NTL--

--------------------PP----------------------------T--AKVSKDA

KECMQ--------ECVSEFISFITSEASDKCLK--EK--RKTINGEDIL--

>NF-YB_HAP3_KLULA#22#87 | 66 | P40914; | PD002982 171 bp

---------EQDRWLPI--NN---------VARLMK-------------------NTL--

--------------------PA----------------------------T--TKVSKDA

KECMQ--------ECVSEFISFVTSEACDRCTS--GK--RKTINGEDIL--

>NF-YB_Q54WV0_DICDI#46#115 | 70 | Q54WV0; | PD002982 171 bp

-------DREQDRYLPI--AN---------IIRIMK-------------------KAL--

--------------------PN----------------------------N--AKVAKDA

KETVQ--------DCVSEFISFITSEASDKCQQ--EK--RKTINGEDIIAA

>NF-YB_HAP3_SCHPO#12#74 | 63 | P36611; | PD002982 171 bp

--------------LPI--AN---------VARIMK-------------------SAL--

--------------------PE----------------------------N--AKISKEA

KDCVQ--------DCVSEFISFVTGEASEQCTQ--EK--RKTITGEDVLLA

>NF-YB_Q8LJQ5_PHACN#53#121 | 69 | Q8LJQ5; | PD002982 171 bp

--------REQDRFMPI--AN---------VIRIMR-------------------KIL--

--------------------PP----------------------------H--AKISGDA

KETIQ--------ECVSEYISFITGEANERCQR--EQ--RKTITAEDVLWA

>NF-YB_Q5TIQ8_HELAN#47#115 | 69 | Q5TIQ8; | PD002982 171 bp

--------REQDRFMPI--AN---------VIRIMR-------------------KIL--

--------------------PP----------------------------H--AKISDDA

KETIQ--------ECVSEYISFVTGEANDRCQR--EQ--RKTITAEDVLWA

>NF-YB_Q94EJ7_MAIZE#36#104 | 69 | Q94EJ7; | PD002982 171 bp

--------REQDRLMPI--AN---------VIRIMR-------------------RVL--

--------------------PA----------------------------H--AKISDDA

KETIQ--------ECVSEYISFITGEANERCQR--EQ--RKTITAEDVLWA

>NF-YB_Q84N51_ORYSA#31#99 | 69 | Q84N51; | PD002982 171 bp

--------REQDRLMPI--AN---------VIRIMR-------------------RVL--

--------------------PA----------------------------H--AKISDDA

KETIQ--------ECVSEYISFITGEANERCQR--EQ--RKTITAEDVLWA

>NF-YB_Q6ZFM9_ORYSA#31#99 | 69 | Q6ZFM9; | PD002982 171 bp

--------REQDRLMPI--AN---------VIRIMR-------------------RVL--

--------------------PA----------------------------H--AKISDDA

KETIQ--------ECVSEYISFITGEANERCQR--EQ--RKTITAEDVLWA

>NF-YB_Q6YNJ7_ORYSA#31#99 | 69 | Q6YNJ7; | PD002982 171 bp

--------REQDRLMPI--AN---------VIRIMR-------------------RVL--

--------------------PA----------------------------H--AKISDDA

KETIQ--------ECVSEYISFITGEANERCQR--EQ--RKTITAEDVLWA

>NF-YB_NFYB6_ARATH#57#125 | 69 | Q84W66; | PD002982 171 bp

--------REQDRFMPI--AN---------VIRIMR-------------------RIL--

--------------------PA----------------------------H--AKISDDS

KETIQ--------ECVSEYISFITGEANERCQR--EQ--RKTITAEDVLWA

>NF-YB_NFYB9_ARATH#28#96 | 69 | Q9SFD8; | PD002982 171 bp

--------REQDQYMPI--AN---------VIRIMR-------------------KTL--

--------------------PS----------------------------H--AKISDDA

KETIQ--------ECVSEYISFVTGEANERCQR--EQ--RKTITAEDILWA

>NF-YB_Q6YNJ9_ORYSA#30#98 | 69 | Q6YNJ9; | PD002982 171 bp

--------REQDRLMPI--AN---------VIRIMR-------------------RVL--

--------------------PP----------------------------H--AKISDDA

KEVIQ--------ECVSEFISFVTGEANDRCHR--EH--RKTVTAEDLVWA

>NF-YB_Q5VNF4_ORYSA#30#98 | 69 | Q5VNF4; | PD002982 171 bp

--------REQDRLMPI--AN---------VIRIMR-------------------RVL--

--------------------PP----------------------------H--AKISDDA

KEVIQ--------ECVSEFISFVTGEANDRCHR--EH--RKTVTAEDLVWA

>NF-YB_Q76FX3_DAUCA#47#115 | 69 | Q76FX3; | PD002982 171 bp

--------REQDRFMPI--AN---------VIRLMR-------------------KSI--

--------------------PS----------------------------H--AKISDDA

KELVQ--------ESVSEFISFVTSEANYRCQK--EQ--RKTITAEDVLWA

>NF-YB_Q75XY2_DAUCA#47#115 | 69 | Q75XY2; | PD002982 171 bp

--------REQDRFMPI--AN---------VIRLMR-------------------KSI--

--------------------PS----------------------------H--AKISDDA

KELVQ--------ESVSEFISFVTSEANYRCQK--EQ--RKTITAEDVLWA

>NF-YB_NFYB4_ARATH#4#70 | 67 | O04027; | PD002982 171 bp

----------EDRLLPI--AN---------VGRLMK-------------------QIL--

--------------------PS----------------------------N--AKISKEA

KQTVQ--------ECATEFISFVTCEASEKCHR--EN--RKTVNGDDIWWA

>NF-YB_Q5JMR1_ORYSA#84#147 | 64 | Q5JMR1; | PD002982 171 bp

----------QDNLLPI--AN---------VGRIMK-------------------DGL--

--------------------PP----------------------------Q--AKISKRA

KETIQ--------ECATEFISFVTGEASERCRR--ER--RKTVNGDDV---

>NF-YB_Q4P0X5_USTMA#514#579 | 66 | Q4P0X5; | PD002982 171 bp

-----------DPDLPI--AN---------ISRIMK-------------------RSL--

--------------------PE----------------------------N--AKIAKDA

KECVQ--------ACVSELISFITSEASDKCAA--EK--RKTINGDDILYA

>NF-YB_Q6BNH7_DEBHA#17#83 | 67 | Q6BNH7; | PD002982 171 bp

--------KEQDRFLPI--AN---------VGRVMK-------------------KAL--

--------------------PP----------------------------H--AKLSKES

KECIQ--------ECVSEFISFITSHASDRGRL--EK--RKTLNGEDIL--

>NF-YB_Q5AMJ9_CANAL#1#54 | 54 | Q5AMJ9; | PD002982 171 bp

----------------------------------MK-------------------KAL--

--------------------PE----------------------------H--AKLSKES

KECIQ--------ECVSEFISFITSQAADRCLV--EK--RKTLNGEDILWA

>NF-YB_Q4I6S1_GIBZE#91#157 | 67 | Q4I6S1; | PD002982 171 bp

----------DANIRNF--AP---------VARIMK-------------------NAL--

--------------------PD----------------------------N--AKIAKEA

KECMQ--------ECVSEFISFITSEASEKCQQ--EK--RKTVNGEDILFA

>NF-YB_Q7SF62_NEUCR#98#164 | 67 | Q7SF62; | PD002982 171 bp

----------DANIRNF--AP---------VARIMK-------------------NAL--

--------------------PE----------------------------N--AKIAKEA

KECMQ--------ECVSEFISFITSEASEKCQQ--EK--RKTVNGEDILFA

>NF-YB_Q8ST61_DROME#37#105 | 69 | Q8ST61; | PD002982 171 bp

--------REQDRFLPI--CN---------IIKIMK-------------------VPV--

--------------------PQ----------------------------N--GKIAKDA

RECIQ--------ECVSEFISFISSEAIERSVA--EN--RKTVNGDDLLVA

>NF-YB_Q8IHQ2_PLAF7#1134#1194 | 61 | Q8IHQ2; | PD002982 171 bp

--------------LPI--AN---------ISRIMK-------------------RIL--

--------------------PG----------------------------S--AKVAKES

KDIIR--------ECVTEFIQFLTSEASDRCTR--EK--RKTINGEDIL--

>NF-YB_Q8WSK5_PLAFA#1134#1194 | 61 | Q8WSK5; | PD002982 171 bp

--------------LPI--AN---------ISRIMK-------------------RIL--

--------------------PG----------------------------S--AKVAKES

KDIIR--------ECVTEFIQFLTSEASDRCTR--EK--RKTINGEDIL--

>NF-YB_Q4XWW3_PLACH#223#284 | 62 | Q4XWW3; | PD002982 171 bp

--------------LPI--AN---------ISRIMK-------------------RIL--

--------------------PA----------------------------K--AKVAKES

KDIIR--------EYVTEFIQFLTSEASDRCLN--EK--RKTINGEDILF-

>NF-YB_Q7RE10_PLAYO#738#799 | 62 | Q7RE10; | PD002982 171 bp

--------------LPI--AN---------ISRIMK-------------------RIL--

--------------------PA----------------------------K--AKVAKES

KDIIR--------EYVTEFIQFLTSEASDRCLN--EK--RKTINGEDILF-

>NF-YB_Q4MYJ6_THEPA#247#314 | 68 | Q4MYJ6; | PD002982 171 bp

---------ENDTSLPI--AN---------IGRLMK-------------------SVL--

--------------------PQ----------------------------S--AKIAKQA

KDMIR--------DCVTEFIFFISSEASDLCNT--ER--RKTLNADDIFVA

>NF-YB_Q61QD1_CAEBR#76#142 | 67 | Q61QD1; | PD002982 171 bp

----------QERYLPI--AN---------VTRLMK-------------------GQM--

--------------------DP----------------------------Q--AKLAKDA

KECVQ--------ECVSEFITFVASEAAEICNQ--QK--RKTIMADDLLTA

>NF-YB_O17286_CAEEL#62#128 | 67 | O17286; | PD002982 171 bp

----------QERFLPI--AN---------VVRIMK-------------------TQM--

--------------------DP----------------------------Q--AKLAKDA

KECAQ--------ECVSEFISFIASEAAEICNI--TK--RKTITADDLLTA

>NF-YB_Q8SR74_ENCCU#9#77 | 69 | Q8SR74; | PD002982 171 bp

--------RPTDRSLPI--AN---------ISKIMK-------------------KPI--

--------------------PK----------------------------E--AKVAKDA

KEIMQ--------KSAGEFIAIITCRAKEICES--EA--RKTVTGEDLIRA

>NF-YB_Q7R612_GIALA#4#73 | 70 | Q7R612; | PD002982 171 bp

-------ERTTDPFLPI--AN---------IGSIMK-------------------ECL--

--------------------PA----------------------------N--TKLTRGA

KELVQ--------EAVTELICFVALQAQTYAVS--HR--RKTVNGNDIITA

>NF-YB_NFYB1_ORYSA#34#94 | 61 | Q6Z348; | PD002982 171 bp

--------------LPM--AN---------LVRLIK-------------------KVL--

--------------------PG----------------------------K--AKIGGAA

KGLTH--------DCAVEFVGFVGDEASEKAKA--EH--RRTVAPEDYL--

>NF-YB_Q5CF64_CRYHO#52#117 | 66 | Q5CF64; | PD002982 171 bp

-----------DLSLPI--NN---------IGRMMK-------------------LSI--

--------------------PG----------------------------S--AKISRES

KMLMQ--------QISKDFIGCISSQAGVICTS--NK--RRVLNGEDIINA

>NF-YB_Q55DJ5_DICDI#6#75 | 70 | Q55DJ5; | PD002982 171 bp

-------DKEDNLSLPK--AT---------VSKLIK-------------------EML--

--------------------PQ----------------------------D--VKCSNET

RDLIL--------ECCVEFIHLISSEANDICGR--EQ--KRTIAAEHVIKA

>NF-YB_Q4P9Q4_USTMA#16#82 | 67 | Q4P9Q4; | PD002982 171 bp

----------DELSLPK--AT---------VQKLIS-------------------EML--

--------------------PK----------------------------D--VSCSKDT

RDLLI--------ECCVEFIHLLSSESNEVCEK--DS--KKTIAPEHVLKA

>NF-YB_O14348_SCHPO#8#74 | 67 | O14348; | PD002982 171 bp

----------DELSLPK--AT---------VQKMVS-------------------DIL--

--------------------PV----------------------------D--LTFTKEA

RDLLI--------ECCVEFIHLVSSEANEICEK--EA--KKTIAAEHIIKA

>NF-YB_Q6C5P7_YARLI#19#78 | 60 | Q6C5P7; | PD002982 171 bp

-------------------AT---------VQKIVS-------------------EII--

--------------------PS----------------------------D--LAFAKDT

RDVLI--------ECCIEFIMMLSTESNEIAEK--ES--KKTIAPEHVIKA

>NF-YB_Q8W0W5_SOYBN#12#78 | 67 | Q8W0W5; | PD002982 171 bp

----------EDASLPK--AT---------MTKIIK-------------------EML--

--------------------PP----------------------------D--VRVARDA

QDLLI--------ECCVEFINLVSSESNEVCNK--EE--RRTIAPEHVLKA

>NF-YB_DR1_ARATH#12#78 | 67 | P49592; | PD002982 171 bp

----------EDASLPK--AT---------MTKIIK-------------------EML--

--------------------PP----------------------------D--VRVARDA

QDLLI--------ECCVEFINLVSSESNDVCNK--ED--KRTIAPEHVLKA

>NF-YB_Q944I5_ARATH#12#78 | 67 | Q944I5; | PD002982 171 bp

----------EDASLPK--AT---------MTKIIK-------------------EML--

--------------------PA----------------------------D--VRVARDA

QDLLI--------ECCVEFINLISSESNEVCNK--ED--KRTIAPEHVLKA

>NF-YB_Q9LEY8_ARATH#12#78 | 67 | Q9LEY8; | PD002982 171 bp

----------EDASLPK--AT---------MTKIIK-------------------EML--

--------------------PA----------------------------D--VRVARDA

QDLLI--------ECCVEFINLISSESNEVCNK--ED--KRTIAPEHVLKA

>NF-YB_Q8W0W9_ORYSA#12#78 | 67 | Q8W0W9; | PD002982 171 bp

----------EDVSLPK--ST---------MFKIIK-------------------EML--

--------------------PP----------------------------D--VRVARDA

QDLLV--------ECCVEFINLLSSESNEVCSR--ED--KKTIAPEHVLRA

>NF-YB_Q8W0W8_WHEAT#12#78 | 67 | Q8W0W8; | PD002982 171 bp

----------EDVSLPK--ST---------MTKIIK-------------------EML--

--------------------PP----------------------------D--VRVARDT

QDLLV--------ECCVEFINLLSSESNDVCSR--DD--KKTIAPEHVIRA

>NF-YB_Q3E977_ARATH#6#65 | 60 | Q3E977; | PD002982 171 bp

----IVGKSKEDASLPK-------------------------------------------

--------------------------------------------------D--VRVARDA

QDLLI--------ECCVEFINLVSSESNDVCNK--ED--KRTIAPEHVLKA

>NF-YB_Q4I0P1_GIBZE#1#78 | 78 | Q4I0P1; | PD002982 171 bp

-MSDHEFGGNEDLSLPK--AT---------VQKIVS-------------------EIL--

--------------------PP----------------------------QAGVAFAKEA

RDLLI--------ECCVEFITLISSEANEISEK--EA--KKTIACDHITKA

>NF-YB_Q6MVU4_NEUCR#30#98 | 69 | Q6MVU4; | PD002982 171 bp

----------DDLSLPK--AT---------VQKIVS-------------------EIL--

--------------------PS----------------------------STGLSFAKEA

RDLLI--------DLCVEFISLVSSEANEISEK--ES--KKTIACDHITQA

>NF-YB_Q7SFL5_NEUCR#93#154 | 62 | Q7SFL5; | PD002982 171 bp

-------------------AT---------VQKIVS-------------------EIL--

--------------------PS----------------------------STGLSFAKEA

RDLLI--------DLCVEFISLVSSEANEISEK--ES--KKTIACDHITQA

>NF-YB_Q5AQS2_EMENI#1#77 | 77 | Q5AQS2; | PD002982 171 bp

-MSDREFSSNDDLSLPKVTAT---------VQKIIT-------------------EIL--

--------------------PP----------------------------SSGQSFSKDA

RDLLM--------ECCVEFITLISSEANDISEK--EA--KKTIACEHV---

>NF-YB_Q4WFF8_ASPFU#10#71 | 62 | Q4WFF8; | PD002982 171 bp

---------------PKV-ST---------VQKIIT-------------------EIL--

--------------------PP----------------------------SSGQTFSKDA

RDLLM--------ECCVEFITLISSEANDISEK--EA--KKTIACEHV---

>NF-YB_Q5A0I6_CANAL#10#76 | 67 | Q5A0I6; | PD002982 171 bp

----------EDLSLPK--AT---------VQKIIN-------------------EIL--

--------------------PK----------------------------D--IGIAKEA

REAIT--------ECSIEFIMMLSTQSNDIAEK--EA--KKTIASDHVVKA

>NF-YB_Q6BJ98_DEBHA#10#76 | 67 | Q6BJ98; | PD002982 171 bp

----------EDLSLPK--AT---------AQKIIG-------------------EIL--

--------------------PK----------------------------D--IAISKEA

REAIT--------ECSIEFIMILSTQLNDIAEK--EA--KKTIASDHVVKA

>NF-YB_Q6CU97_KLULA#1#71 | 71 | Q6CU97; | PD002982 171 bp

-----MVGEVDDVTLPK--AT---------VQKIIS-------------------EVL--

--------------------DS----------------------------D--LTFSKEA

REIII--------ECGVEFIMMLAGSASEIADK--EL--KKTIAPDHVIK-

>NF-YB_Q753R3_ASHGO#1#71 | 71 | Q753R3; | PD002982 171 bp

------MGEADDITLPK--AT---------VQKIIS-------------------EVL--

--------------------DS----------------------------E--LTFSKEA

REIII--------DAGIEFIMILSGMASEMAES--EA--KKTIAPDHVIKA

>NF-YB_Q6FK35_CANGA#6#72 | 67 | Q6FK35; | PD002982 171 bp

----------DDVTLPK--AT---------VQKIIS-------------------EVL--

--------------------DS----------------------------D--LMFNRDA

REIII--------KSGIEFIMILSSMASEMAES--DA--KKTIAPEHVIKA

>NF-YB_Q92317_YEAST#1#72 | 72 | Q92317; | PD002982 171 bp

-----MAGDSDNVSLPK--AT---------VQKMIS-------------------EIL--

--------------------DQ----------------------------D--LMFTKDA

REIII--------NSGIEFIMILSSMASEMADN--EA--KKTIAPEHVIKA

>NF-YB_Q04176_YEAST#10#69 | 60 | Q04176; | PD002982 171 bp

-------------------AT---------VQKMIS-------------------EIL--

--------------------DQ----------------------------D--LMFTKDA

REIII--------NSGIEFIMILSSMASEMADN--EA--KKTIAPEHVIKA

>NF-YB_TBAP_RAT#9#74 | 66 | Q5XI68; | PD002982 171 bp

----------DDLTIPR--AA---------INKMIK-------------------ETL--

--------------------PN-------------------------------VRVANDA

RELVV--------NCCTEFIHLISSEANEICNK--SE--KKTISPEHVIQA

>NF-YB_TBAP_MOUSE#9#74 | 66 | Q91WV0; | PD002982 171 bp

----------DDLTIPR--AA---------INKMIK-------------------ETL--

--------------------PN-------------------------------VRVANDA

RELVV--------NCCTEFIHLISSEANEICNK--SE--KKTISPEHVIQA

>NF-YB_TBAP_HUMAN#9#74 | 66 | Q01658; | PD002982 171 bp

----------DDLTIPR--AA---------INKMIK-------------------ETL--

--------------------PN-------------------------------VRVANDA

RELVV--------NCCTEFIHLISSEANEICNK--SE--KKTISPEHVIQA

>NF-YB_TBAP_CHICK#9#74 | 66 | Q5ZMV3; | PD002982 171 bp

----------DDLTIPR--AA---------INKMIK-------------------ETL--

--------------------PN-------------------------------VRVANDA

RELVV--------NCCTEFIHLISSEANEICNK--SE--KKTISPEHVIQA

>NF-YB_Q658N3_HUMAN#9#74 | 66 | Q658N3; | PD002982 171 bp

----------DDLTIPR--AA---------INKMIK-------------------ETL--

--------------------PN-------------------------------VRVANDA

RELVV--------NCCTEFIHLISSEANEICNK--SE--KKTISPEHVIQA

>NF-YB_Q3UT14_MOUSE#9#74 | 66 | Q3UT14; | PD002982 171 bp

----------DDLTIPR--AA---------INKMIK-------------------ETL--

--------------------PN-------------------------------VRVANDA

RELVV--------NCCTEFIHLISSEANEICNK--SE--KKTISPEHVIQA

>NF-YB_Q3UMQ2_MOUSE#9#74 | 66 | Q3UMQ2; | PD002982 171 bp

----------DDLTIPR--AA---------INKMIK-------------------ETL--

--------------------PN-------------------------------VRVANDA

RELVV--------NCCTEFIHLISSEANEICNK--SE--KKTISPEHVIQA

>NF-YB_O13068_9PIPI#9#74 | 66 | O13068; | PD002982 171 bp

----------DDLTIPR--AA---------INKMIK-------------------ETL--

--------------------PS-------------------------------VRVANDA

RELVV--------NCCTEFIHLISSEANEICNK--SE--KKTISPEHVIQA

>NF-YB_Q7Q2H7_ANOGA#14#81 | 68 | Q7Q2H7; | PD002982 171 bp

--------EDDELTLPR--AS---------INKIIK-------------------ELV--

--------------------PS-------------------------------IRVANES

RELIL--------NCCTEFIHLISSEANEVCNQ--RN--KKTINAEHVLEA

>NF-YB_NC2B_DROME#14#81 | 68 | Q9VJQ5; | PD002982 171 bp

--------EDDELTLPR--AS---------INKIIK-------------------ELV--

--------------------PT-------------------------------VRVANES

RELIL--------NCCSEFIHLISSEANEVCNM--RN--KKTINAEHVLEA

>NF-YB_Q5KIE5_CRYNE#8#75 | 68 | Q5KIE5; | PD002982 171 bp

---------EDDVSLPK--AT---------VLKIIQ-------------------EML--

--------------------PE----------------------------D--ISASKEA

KDIIF--------DCCTEWIKLISTQSNMVCEA--SS--KKTISPEHVVEA

>NF-YB_Q55TZ4_CRYNE#8#75 | 68 | Q55TZ4; | PD002982 171 bp

---------EDDVSLPK--AT---------VLKIIQ-------------------EML--

--------------------PE----------------------------D--ISASKEA

KDIIF--------DCCTEWIKLISTQSNMVCEA--SS--KKTISPEHVVEA

>NF-YB_O45550_CAEEL#27#83 | 57 | O45550; | PD002982 171 bp

------------------------------INQIIK-------------------EVV--

--------------------PE-------------------------------MRIANES

RDMIN--------ACCVEFVKHIAREAQRIASQ--DQ--RKTIYHEHVQKA

>NF-YB_Q60K06_CAEBR#14#70 | 57 | Q60K06; | PD002982 171 bp

------------------------------INQIVK-------------------EII--

--------------------PD-------------------------------VRIANES

RDMIN--------ACCVEFVKHVAREAQKIASQ--DQ--RKTIYHEHVQKA

>NF-YB_Q516E9_ENTHI#7#69 | 63 | Q516E9; | PD002982 171 bp

--------------LPK--TS---------INKLIK-------------------ENL--

--------------------SN----------------------------S--IRVSADF

RDVVA--------DCGVEFIHIIAAQAKDVAAN--TN--RKTLSTDHVLTA

>NF-YB_Q8SQX5_ENCCU#4#74 | 71 | Q8SQX5; | PD002982 171 bp

------EKNDDENTLPK--AT---------VDKMVS-------------------SML--

--------------------PK----------------------------N--SVVPKES

KEIFQ--------NACIYFLNMLTLEANKACEE--EK--KKTISYEHVYKA

>NF-YB_Q9BI15_ENTDI#25#97 | 73 | Q9BI15; | PD002982 171 bp

----------DSLSLPV--AN---------TIRVMR-------------------KSV--

------------------SMPN----------------------------GSAVRISKDA

QEYMT--------ELATEFLSFIASEAADVPKGSVKS--KHTLTGADVIDA

>NF-YB_Q50VB7_ENTHI#27#97 | 71 | Q50VB7; | PD002982 171 bp

------------LSLPV--AN---------TTRVMK-------------------NSV--

------------------SMPN----------------------------GSAVRISKDA

QEYMT--------EVATEFLSFIASEAADVPKGSVKP--KHTLTGTDIIDA

>NF-YB_Q75JQ9_DICDI#3#69 | 67 | Q75JQ9; | PD002982 171 bp

----------ESQDLPG--AI---------VNRIIK-------------------ASL--

--------------------PE----------------------------G--VLCAKES

RLAIA--------KAAKVWIHYLTAASIDFSSH--SG--RSTISPKDVFQA

>NF-YB_Q9ZQH2_ARATH#11#78 | 68 | Q9ZQH2; | PD002982 171 bp

--------------LPL--AI---------VRRVVK-------------------KKL--

--------------------SE------------------------CSP-DYDVSIHKEA

LLAFS--------ESARIFIHYLSATANDFCKD--AR--RQTMKADDVFKA

>NF-YB_Q652Q3_ORYSA#36#104 | 69 | Q652Q3; | PD002982 171 bp

--------------LPK--AI---------VRRLVK-------------------EKL--

--------------------AQ------------------------VAAGGAEVIVNKDA

MSAFA--------ESARIFIHYLSATANDMCKE--SK--RQTINADDVLKA

>NF-YB_Q381S9_9TRYP#51#110 | 60 | Q381S9; | PD002982 171 bp

------------------------------VDRIVA-------------------AAV--

--------------------PA------------------------------GMHVARDA

RIAMQ--------KAATISLLYLSCLADDEHSRESRR--RVTLSANDIKTA

>NF-YB_Q4DTC9_TRYCR#53#112 | 60 | Q4DTC9; | PD002982 171 bp

------------------------------VDRTVS-------------------AAL--

--------------------PD------------------------------GMHVSKDA

RIAFQ--------KAATLFLLYLSCLAEDERSREGRK--RVTLSAHDIKSA

>NF-YB_Q4D3L1_TRYCR#111#170 | 60 | Q4D3L1; | PD002982 171 bp

------------------------------VDRTVS-------------------AAL--

--------------------PD------------------------------GMHVSKDA

RIAFQ--------KAATLFLLYLSCLAEDERSREGKK--RVTLSAHDIKSA

>NF-YB_Q4Q591_LEIMA#77#133 | 57 | Q4Q591; | PD002982 171 bp

------------------------------VNRVVH-------------------GAL--

--------------------PE------------------------------GMSVSRDA

RVALQ--------KSATISVLYLACLGDASRKATGST--RTTLNVQDI---

>NF-YB_HMVA_METVO#3#59 | 57 | Q03576; | PD002982 171 bp

---------------PK--GT---------VKRIMK-------------------DNT--

--------------------------------------------------E--MYVSTES

VVALV--------DILQEMIVTTTKIAEENAAK--DK--RKTIKARDI---

>NF-YB_Q6LYH6_METMP#16#72 | 57 | Q6LYH6; | PD002982 171 bp

---------------PK--GT---------VKRIMK-------------------ENT--

--------------------------------------------------D--MNVSAES

VVALV--------EILQEMVVTTTKIAEENAAK--DK--RKTLKARDI---

>NF-YB_HMVA_METJA#2#59 | 58 | Q59041; | PD002982 171 bp

--------------LPK--AT---------VKRIMK-------------------QHT--

--------------------------------------------------D--FNISAEA

VDELC--------NMLEEIIKITTEVAEQNARK--EG--RKTIKARDI---

>NF-YB_H2A_ENCCU#32#87 | 56 | Q8SSG3; | PD002982 171 bp

------------------------------IKKIMK-------------------DRT--

--------------------------------------------------R--MRISKDA

LVAVS--------ACVMYLISEITDGAKNVAST--DG--KKKVMPKHINNA

>NF-YB_Q4Z6R3_PLABE#19#82 | 64 | Q4Z6R3; | PD002982 171 bp

-------------DLPN--SV---------ILKIIN-------------------NSI--

-----------------------------------------------DLKN--YKIRKEA

LNTLS--------KCLSLFILYITDGALEHCEN--EK--RFTIFVRDILN-

>NF-YB_Q7RB92_PLAYO#19#82 | 64 | Q7RB92; | PD002982 171 bp

-------------DLPN--SV---------ILKIIN-------------------NSI--

-----------------------------------------------DLKN--YKIRKEA

LNTLS--------KCLSLFILYITDGALEHCES--EK--RFTIFVRDILN-

>NF-YB_Q8IEN4_PLAF7#24#82 | 59 | Q8IEN4; | PD002982 171 bp

--------------------I---------IQKAIN-------------------NNV--

-----------------------------------------------DLRN--YRMRKEA

LETLG--------KCLSMFILYITDGAMEYCEN--EK--RSTILVRDILN-

>NF-YB_Q6CY26_KLULA#75#137 | 63 | Q6CY26; | PD002982 171 bp

-------------SLPL--AR---------IKKVMK---TDEEV-----------R----

------------------------------------------------------MISGEA

PILFA--------KACEIFITELTMRAWCVAEE--NK--RRTLQKQDIADA

>NF-YB_Q758Y6_ASHGO#64#126 | 63 | Q758Y6; | PD002982 171 bp

-------------SLPL--AR---------IKKVMK---TDEDV-----------K----

------------------------------------------------------MISAEA

PILFA--------KACEIFITELTMRAWCIAEE--NK--RRTLQKQDIAQA

>NF-YB_Q6FM95_CANGA#113#175 | 63 | Q6FM95; | PD002982 171 bp

-------------SLPF--AR---------IRRVMK---TDEEV-----------K----

------------------------------------------------------MISAEA

PIIFA--------KACEVFITELTMRAWCVAEK--HK--RRTLQKADIAEA

>NF-YB_HAP5_YEAST#159#221 | 63 | Q02516; | PD002982 171 bp

-------------SLPF--AR---------IRKVMK---TDEDV-----------K----

------------------------------------------------------MISAEA

PIIFA--------KACEIFITELTMRAWCVAER--NK--RRTLQKADIAEA

>NF-YB_NFYC4_ARATH#79#140 | 62 | Q9FMV5; | PD002982 171 bp

--------------LPL--AR---------IKKIMK---ADEDV-----------R----

------------------------------------------------------MISAEA

PILFA--------KACELFILELTIRSWLHAEE--NK--RRTLQKNDIAAA

>NF-YB_NFYC1_ARATH#66#127 | 62 | Q9SMP0; | PD002982 171 bp

--------------LPL--AR---------IKKIMK---ADEDV-----------R----

------------------------------------------------------MISAEA

PILFA--------KACELFILELTIRSWLHAEE--NK--RRTLQKNDIAAA

>NF-YB_Q76FX1_DAUCA#101#163 | 63 | Q76FX1; | PD002982 171 bp

-------------SLPL--AR---------IKKIMK---ADEDV-----------R----

------------------------------------------------------MISSEA

PVIFA--------KACEMFILELTMRSWLLTEE--NK--RRTLQKNDIAAA

>NF-YB_Q76FX2_DAUCA#82#144 | 63 | Q76FX2; | PD002982 171 bp

-------------SLPL--AR---------IKKIMK---ADEDV-----------R----

------------------------------------------------------MISSEA

PVVFA--------KACEMFIMDLTMRSWSHTEE--NK--RRTLQKNDIAAA

>NF-YB_Q38HS7_SOLTU#100#162 | 63 | Q38HS7; | PD002982 171 bp

-------------SLPL--AR---------IKKIMK---ADEDV-----------R----

------------------------------------------------------MISAEA

PVVFA--------RACEMFILELTLRAWNHTEE--NK--RRTLQKNDIAAA

>NF-YB_NFYC9_ARATH#80#142 | 63 | Q8L4B2; | PD002982 171 bp

-------------SLPL--AR---------IKKIMK---ADEDV-----------R----

------------------------------------------------------MISAEA

PVVFA--------RACEMFILELTLRSWNHTEE--NK--RRTLQKNDIAAA

>NF-YB_NFYC3_ARATH#70#132 | 63 | Q9ZVL3; | PD002982 171 bp

-------------SLPL--AR---------IKKIMK---ADEDV-----------R----

------------------------------------------------------MISAEA

PVVFA--------RACEMFILELTLRSWNHTEE--NK--RRTLQKNDIAAA

>NF-YB_Q6H707_ORYSA#105#167 | 63 | Q6H707; | PD002982 171 bp

-------------SLPL--AR---------IKKIMK---ADEDV-----------R----

------------------------------------------------------MISAEA

PVVFA--------KACEVFILELTLRSWMHTEE--NK--RRTLQKNDIAAA

>NF-YB_Q655V5_ORYSA#101#163 | 63 | Q655V5; | PD002982 171 bp

-------------SLPL--AR---------IKKIMK---ADEDV-----------R----

------------------------------------------------------MISAEA

PVIFA--------KACEIFILELTLRSWMHTEE--NK--RRTLQKNDIAAA

>NF-YB_NFYC2_ARATH#76#138 | 63 | Q8LCG7; | PD002982 171 bp

-------------TLPL--AR---------IKKIMK---ADEDV-----------R----

------------------------------------------------------MISAEA

PVIFA--------KACEMFILELTLRAWIHTEE--NK--RRTLQKNDIAAA

>NF-YB_Q9XE33_ORYSA#90#152 | 63 | Q9XE33; | PD002982 171 bp

-------------NLPL--AR---------IKKIMK---ADEDV-----------R----

------------------------------------------------------MIAAEA

PVVFA--------RACEMFILELTHRGWAHAEE--NK--RRTLQKSDIAAA

>NF-YB_Q6BS91_DEBHA#105#166 | 62 | Q6BS91; | PD002982 171 bp

--------------LPL--AR---------IKKVMK---TDEDV-----------R----

------------------------------------------------------MISAEA

PILFA--------KGCDVFITELTMRAWIHAEE--NK--RRTLQKSDIAAA

>NF-YB_Q5A1M2_CANAL#146#207 | 62 | Q5A1M2; | PD002982 171 bp

--------------LPL--AR---------IKKVMK---TDEDV-----------R----

------------------------------------------------------MISAEA

PILFA--------KGCDVFITELTMRAWIHAEE--NK--RRTLQKSDIAAA

>NF-YB_Q6CFV6_YARLI#92#153 | 62 | Q6CFV6; | PD002982 171 bp

--------------LPL--AR---------IKKVMK---ADEDV-----------K----

------------------------------------------------------MISAEA

PILFA--------KGCDIFITELSMRAWIHAEE--HK--RRTLQRSDIASA

>NF-YB_Q5AYY8_EMENI#87#148 | 62 | Q5AYY8; | PD002982 171 bp

--------------LPL--AR---------IKKVMK---ADPEV-----------K----

------------------------------------------------------MISAEA

PILFA--------KGCDVFITELTMRAWIHAED--NK--RRTLQRSDIAAA

>NF-YB_P87092_EMENI#87#148 | 62 | P87092; | PD002982 171 bp

--------------LPL--AR---------IKKVMK---ADPEV-----------K----

------------------------------------------------------MISAEA

PILFA--------KGCDVFITELTMRAWIHAED--NK--RRTLQRSDIAAA

>NF-YB_Q4WDM9_ASPFU#87#148 | 62 | Q4WDM9; | PD002982 171 bp

--------------LPL--AR---------IKKVMK---ADPEV-----------K----

------------------------------------------------------MISAEA

PILFA--------KGCDIFITELTMRAWIHAED--NK--RRTLQRSDIAAA

>NF-YB_O59849_ASPOR#87#148 | 62 | O59849; | PD002982 171 bp

--------------LPL--AR---------IKKVMK---ADPEV-----------K----

------------------------------------------------------MISAEA

PILFA--------KGCDIFITELTMRAWIHAED--NK--RRTLQRSDIAAA

>NF-YB_Q7Z8M0_ASPNG#87#148 | 62 | Q7Z8M0; | PD002982 171 bp

--------------LPL--AR---------IKKVMK---ADPEV-----------K----

------------------------------------------------------MISAEA

PILFA--------KGCDIFITELTMRAWTHAED--NK--RRTLQRSDIAAA

>NF-YB_Q96WN9_TRIRE#88#149 | 62 | Q96WN9; | PD002982 171 bp

--------------LPL--AR---------IKKVMK---ADPEV-----------K----

------------------------------------------------------MISAEA

PILFA--------KGCDIFITELTMRAWIHAEE--NK--RRTLQRSDIASA

>NF-YB_Q4IJK0_GIBZE#85#146 | 62 | Q4IJK0; | PD002982 171 bp

--------------LPL--AR---------IKKVMK---ADPEV-----------K----

------------------------------------------------------MISAEA

PILFA--------KGCDIFITELTMRAWIHAEE--NK--RRTLQRSDIASA

>NF-YB_O13381_NEUCR#88#149 | 62 | O13381; | PD002982 171 bp

--------------LPL--AR---------IKKVMK---ADPEV-----------K----

------------------------------------------------------MISAEA

PILFA--------KGCDIFITELTMRAWIHAEE--NK--RRTLQRSDIASA

>NF-YB_Q7RVQ7_NEUCR#79#140 | 62 | Q7RVQ7; | PD002982 171 bp

--------------LPL--AR---------IKKVMK---ADPEV-----------K----

------------------------------------------------------MISAEA

PILFA--------KGCDIFITELTMRAWIHAEE--NK--RRTLQRSDIASA

>NF-YB_NFYC6_ARATH#55#116 | 62 | Q9FGP7; | PD002982 171 bp

--------------LPL--AR---------IKKIMK---ADPDV-----------H----

------------------------------------------------------MVSAEA

PIIFA--------KACEMFIVDLTMRSWLKAEE--NK--RHTLQKSDISNA

>NF-YB_Q6P4L4_XENTR#38#100 | 63 | Q6P4L4; | PD002982 171 bp

----------RVQDLPL--AR---------IKKIMK---LDEDV-----------K----

------------------------------------------------------MISAEA

PVLFA--------KAAQIFITELTLRAWIHTED--NK--RRTLQRNDI---

>NF-YB_Q6DCR1_XENLA#37#99 | 63 | Q6DCR1; | PD002982 171 bp

----------RVQDLPL--AR---------IKKIMK---LDEDV-----------K----

------------------------------------------------------MISAEA

PVLFA--------KAAQIFITELTLRAWIHTED--NK--RRTLQRNDI---

>NF-YB_O73745_XENLA#37#99 | 63 | O73745; | PD002982 171 bp

----------RVQDLPL--AR---------IKKIMK---LDEDV-----------K----

------------------------------------------------------MISAEA

PVLFA--------KAAQIFITELTLRAWIHTED--NK--RRTLQRNDI---

>NF-YB_NFYC_HUMAN#43#101 | 59 | Q13952; | PD002982 171 bp

--------------LPL--AR---------IKKIMK---LDEDV-----------K----

------------------------------------------------------MISAEA

PVLFA--------KAAQIFITELTLRAWIHTED--NK--RRTLQRNDI---

>NF-YB_NFYC_MOUSE#43#101 | 59 | P70353; | PD002982 171 bp

--------------LPL--AR---------IKKIMK---LDEDV-----------K----

------------------------------------------------------MISAEA

PVLFA--------KAAQIFITELTLRAWIHTED--NK--RRTLQRNDI---

>NF-YB_Q4RNN3_TETNG#41#99 | 59 | Q4RNN3; | PD002982 171 bp

--------------LPL--AR---------IKKIMK---LDEDV-----------K----

------------------------------------------------------MISAEA

PVLFA--------KAAQIFITELTLRAWIHTED--NK--RRTLQRNDI---

>NF-YB_Q7ZVY3_BRARE#41#99 | 59 | Q7ZVY3; | PD002982 171 bp

--------------LPL--AR---------IKKIMK---LDEDV-----------K----

------------------------------------------------------MISAEA

PVLFA--------KAAQIFITELTLRAWIHTED--NK--RRTLQRNDI---

>NF-YB_Q6P0F1_BRARE#40#98 | 59 | Q6P0F1; | PD002982 171 bp

--------------LPL--AR---------IKKIMK---LDEDV-----------K----

------------------------------------------------------MISAEA

PVLFA--------KAAQIFITELTLRAWIHTED--NK--RRTLQRNDI---

>NF-YB_NFYC_BOVIN#43#101 | 59 | Q5E9X1; | PD002982 171 bp

--------------LPL--AR---------IKKIMK---LDEDV-----------K----

------------------------------------------------------MISAEA

PVLFA--------KAAQIFITELTLRAWIHTED--NK--RRTLQRNDI---

>NF-YB_NFYC_PONPY#43#101 | 59 | Q5RA23; | PD002982 171 bp

--------------LPL--AR---------IKKIMK---LDEDV-----------K----

------------------------------------------------------MISAEA

PVLFA--------KAAQIFITELTLRAWIHTED--NK--RRTLQRNDI---

>NF-YB_NFYC_RAT#43#101 | 59 | Q62725; | PD002982 171 bp

--------------LPL--AR---------IKKIMK---LDEDV-----------K----

------------------------------------------------------MISAEA

PVLFA--------KAAQIFITELTLRAWIHTED--NK--RRTLQRNDI---

>NF-YB_Q7TPS7_MOUSE#43#101 | 59 | Q7TPS7; | PD002982 171 bp

--------------LPL--AR---------IKKIMK---LDEDV-----------K----

------------------------------------------------------MISAEA

PVLFA--------KAAQIFITELTLRAWIHTED--NK--RRTLQRNDI---

>NF-YB_Q5T6K9_HUMAN#43#101 | 59 | Q5T6K9; | PD002982 171 bp

--------------LPL--AR---------IKKIMK---LDEDV-----------K----

------------------------------------------------------MISAEA

PVLFA--------KAAQIFITELTLRAWIHTED--NK--RRTLQRNDI---

>NF-YB_HAP5_SCHPO#109#172 | 64 | P79007; | PD002982 171 bp

--------------LPL--AR---------IKKVMK---TDDDV-----------KNK--

------------------------------------------------------MISAEA

PFLFA--------KGSEIFIAELTMRAWLHAKK--NQ--RRTLQRSDIANA

>NF-YB_Q7QFE5_ANOGA#99#161 | 63 | Q7QFE5; | PD002982 171 bp

-------------LLPL--AR---------IKKIMK---LDEEV-----------K----

------------------------------------------------------MISSDA

PLLFS--------KAIEIFIQELTLRAWLHTEH--NK--RRTLQRSDIAMA

>NF-YB_Q55QE0_CRYNE#460#523 | 64 | Q55QE0; | PD002982 171 bp

------------YNLPL--AR---------IKKVMK---SDEEV-----------K----

------------------------------------------------------MISAEA

PIMFS--------KACEIFISELTCRAWLVAES--HK--RRTLQKSDVAAA

>NF-YB_Q5KFQ9_CRYNE#460#523 | 64 | Q5KFQ9; | PD002982 171 bp

------------YNLPL--AR---------IKKVMK---SDEEV-----------K----

------------------------------------------------------MISAEA

PIMFS--------KACEIFISELTCRAWLVAES--HK--RRTLQKSDVAAA

>NF-YB_Q4PDA5_USTMA#90#148 | 59 | Q4PDA5; | PD002982 171 bp

--------------LPL--AR---------IKKVMK---SDDQV-----------K----

------------------------------------------------------MISAEA

PILFA--------RACEIFISDLTCRAFLIAEE--HK--RRTIQRSDV---

>NF-YB_Q6ZKK7_ORYSA#70#129 | 60 | Q6ZKK7; | PD002982 171 bp

-------------NLPL--AR---------IKKIMK---ADEDV-----------K----

------------------------------------------------------MIAGEA

PALFA--------KACEMFILDMTLRSWQHTEE--GR--RRTLQRSDV---

>NF-YB_Q9W3V9_DROME#154#215 | 62 | Q9W3V9; | PD002982 171 bp

--------------LPL--AR---------IKKIMK---LDENA-----------K----

------------------------------------------------------MIAGEA

PLLFA--------KACEYFIQELTMHAWVHTEE--SR--RRTLQRSDIAQA

>NF-YB_NFYC8_ARATH#37#98 | 62 | Q4PSE2; | PD002982 171 bp

--------------LPI--TR---------IKKIMK---YDPDV-----------T----

------------------------------------------------------MIASEA

PILLS--------KACEMFIMDLTMRSWLHAQE--SK--RVTLQKSNVDAA

>NF-YB_NFYC5_ARATH#37#95 | 59 | Q9FGP6; | PD002982 171 bp

--------------FPI--SR---------IKRIMK---FDPDV-----------S----

------------------------------------------------------MIAAEA

PNLLS--------KACEMFVMDLTMRSWLHAQE--SN--RLTIRKSDV---

>NF-YB_Q8SSZ4_DICDI#271#331 | 61 | Q8SSZ4; | PD002982 171 bp

--------------LPL--AR---------IKKIMK---SDKDV-----------N----

------------------------------------------------------KISSEA

PILFA--------KACEILILEMTHRSWVHTEM--NK--RRTLQRTDIIN-

>NF-YB_Q557I1_DICDI#271#331 | 61 | Q557I1; | PD002982 171 bp

--------------LPL--AR---------IKKIMK---SDKDV-----------N----

------------------------------------------------------KISSEA

PILFA--------KACEILILEMTHRSWVHTEM--NK--RRTLQRTDIIN-

>NF-YB_Q55GE1_DICDI#601#647 | 47 | Q55GE1; | PD002982 171 bp

-------------TLPL--AR---------IKKIMK---SDPGV-----------K----

------------------------------------------------------MISWEA

PILFA--------KACEFFILELAARSWIHT--------------------

>NF-YB_NFYC7_ARATH#64#121 | 58 | Q9FGP8; | PD002982 171 bp

--------------FPL--TR---------IKKIMK---SNPEV-----------N----

------------------------------------------------------MVTAEA

PVLIS--------KACEMLILDLTMRSWLHTVE--GG--RQTLKRSD----

>NF-YB_Q7RRA6_PLAYO#35#99 | 65 | Q7RRA6; | PD002982 171 bp

--------------LPI--SR---------IKKIMK---EDDKI-----------KNS--

-----------------------------------------------------QMISADT

PVLLA--------KACELFIMEFTKYAWKYTEE--NK--RRTLQRQDVIAA

>NF-YB_Q8IL74_PLAF7#25#99 | 75 | Q8IL74; | PD002982 171 bp

----MSPEDLKIHNLPI--SR---------IKKIMK---EDDEI-----------KSN--

-----------------------------------------------------QMVSADT

PVLLA--------KACELFIMELTSNAWKYTEE--GK--RRTLQRQDVVSA

>NF-YB_Q7XPV7_ORYSA#24#99 | 76 | Q7XPV7; | PD002982 171 bp

--------------LPL--AR---------IKKIMKRSAGDSSVVDGGGGGGGGAR----

------------------------------------------------------MISGEA

PVVFS--------KACELFIAELTRRAWAATLE--GK--RRTVHKEDVAAA

>NF-YB_NFYCA_ARATH#68#129 | 62 | Q58CM8; | PD002982 171 bp

--------------LPL--SR---------VRKILK---SDPEV-----------K----

------------------------------------------------------KISCDV

PALFS--------KACEYFILEVTLRAWMHTQS--CT--RETIRRCDIFQA

>NF-YB_Q8SRY9_ENCCU#28#100 | 73 | Q8SRY9; | PD002982 171 bp

---MEERILLKDLNLPL--AR---------IKRLMK---IEEGV-----------R----

------------------------------------------------------MVASEV

PVLFS--------MITEKFIEELTLRAWINTEE--NK--RRILQKSDLTAA

>NF-YB_Q4UHC3_THEAN#34#95 | 62 | Q4UHC3; | PD002982 171 bp

--------------LPV--AR---------VKKIMK---ETEHQ-----------G----

------------------------------------------------------MISSDA

PVILA--------KACEMLIRDLTLQSWNCTQM--TK--RCTLQRQDIKSA

>NF-YB_Q4N886_THEPA#34#95 | 62 | Q4N886; | PD002982 171 bp

--------------LPV--AR---------VKKIMK---ETEHQ-----------G----

------------------------------------------------------MISSDA

PVILA--------KACEMLIRDLTLQSWNCTQL--TK--RCTLQRQDIKTA

>NF-YB_O17072_CAEEL#101#173 | 73 | O17072; | PD002982 171 bp

-----MLNKSKNMSVPM--AR---------VKKIMR---IDDDV-----------RNF--

------------------------------------------------------MIASDA

PIFMA--------QAAEFFIEEMTAMGWQYVSE--AR--RRILQKADIASA

>NF-YB_Q61QI8_CAEBR#130#198 | 69 | Q61QI8; | PD002982 171 bp

-----MATKSKNMSVPM--AR---------VKKIMK---IDED-----------------

------------------------------------------------------NIASDA

PIFMA--------QAAEFFIEEMTAMGWQYVSE--AR--RRILQKSDVATA

>NF-YB_Q3TJ13_MOUSE#42#103 | 62 | Q3TJ13; | PD002982 171 bp

--------------LPL--AR---------VKALVK---ADPDV-----------T----

------------------------------------------------------LAGQEA

IFILA--------RAAELFVETIAKDAYCCAQQ--GK--RKTLQRRDLDNA

>NF-YB_DPOE4_MOUSE#42#103 | 62 | Q9CQ36; | PD002982 171 bp

--------------LPL--AR---------VKALVK---ADPDV-----------T----

------------------------------------------------------LAGQEA

IFILA--------RAAELFVETIAKDAYCCAQQ--GK--RKTLQRRDLDNA

>NF-YB_DPOE4_HUMAN#41#102 | 62 | Q9NR33; | PD002982 171 bp

--------------LPL--AR---------VKALVK---ADPDV-----------T----

------------------------------------------------------LAGQEA

IFILA--------RAAELFVETIAKDAYCCAQQ--GK--RKTLQRRDLDNA

>NF-YB_Q4RUG3_TETNG#59#120 | 62 | Q4RUG3; | PD002982 171 bp

--------------LPL--AR---------IKALMK---TDPDV-----------S----

------------------------------------------------------LASQES

VFIIA--------KATELFVEMIAKDALVYAQQ--GK--RKTLQRKDLDNA

>NF-YB_Q503Q5_BRARE#51#112 | 62 | Q503Q5; | PD002982 171 bp

--------------LPL--SR---------IKTLMK---ADPDV-----------T----

------------------------------------------------------LASQES

VFIIA--------KATELFVEMIAKDALVYAQQ--GK--RKTLQRKDLDNA

>NF-YB_Q9W256_DROME#78#136 | 59 | Q9W256; | PD002982 171 bp

--------------LPL--AR---------IRNIMK---LDPDL-----------H----

------------------------------------------------------MANNEA

VFIVA--------KAVELFIASLSRESYTYTAQ--SK--KKTIQKRDV---

>NF-YB_Q8MSF2_DROME#56#114 | 59 | Q8MSF2; | PD002982 171 bp

--------------LPL--AR---------IRNIMK---LDPDL-----------H----

------------------------------------------------------MANNEA

VFIVA--------KAVELFIASLSRESYTYTAQ--SK--KKTIQKRDV---

>NF-YB_Q9NAC5_CAEEL#36#97 | 62 | Q9NAC5; | PD002982 171 bp

--------------LPL--GR---------VKKVVR---MNPDV-----------E----

------------------------------------------------------MLNNEA

LQLMA--------KAAELFIKELSNAANQNAAL--EK--RKTVQTKDIDKA

>NF-YB_Q60SG9_CAEBR#26#97 | 72 | Q60SG9; | PD002982 171 bp

----EKVQDLVKTQLPL--GR---------VKKIVR---LNPDV-----------E----

------------------------------------------------------MLNAEA

LQMMT--------KSAELFIKELSNAANQNALT--EK--RKTIQPKDIDKA

>NF-YB_Q5AHY9_CANAL#61#125 | 65 | Q5AHY9; | PD002982 171 bp

-----------NLTLPI--SK---------IKKIFK---MDPEY-----------T----

------------------------------------------------------GASASA

VYTAG--------LATELFVQYFAEQASLLAKM--EK--RKKIQYKDFSNA

>NF-YB_DPB3_DEBHA#90#153 | 64 | Q6BX14; | PD002982 171 bp

------------LSLPL--SK---------IKKIFK---MDPDY-----------L----

------------------------------------------------------AASQSA

VYATG--------LATELFIQYFTEQSLVLAKM--DK--RKKLQYKDFSNA

>NF-YB_Q4WV32_ASPFU#27#90 | 64 | Q4WV32; | PD002982 171 bp

--------------LPI--TR---------IKKIIH---LDEDI-----------V----

------------------------------------------------------QCSGNA

TFVVA--------KATEMFIQYLAQQGHNVVKS--ERKPRKVIQYKDLATA

>NF-YB_Q7S9P3_NEUCR#20#83 | 64 | Q7S9P3; | PD002982 171 bp

--------------LPL--TR---------VKKIIA---VDPDI-----------T----

------------------------------------------------------VCSNNA

AFVIT--------LATEMFIQHLASEAQNMAKA--ERKPRRNVQYKDVAAA

>NF-YB_Q4IBV4_GIBZE#20#83 | 64 | Q4IBV4; | PD002982 171 bp

--------------LPL--SR---------VKKIIA---QDPEI-----------A----

------------------------------------------------------LCSNNA

AFVIT--------LAAEMFVQHLAEESHKQAKL--DRKPRRNIQYKDVASA

>NF-YB_Q5KDK2_CRYNE#106#166 | 61 | Q5KDK2; | PD002982 171 bp

--------------FPA--AR---------VKKIVK---ADRDI-----------D----

------------------------------------------------------IMSSEA

VFMVS--------VAAEYFIKHFMEEGYTKARL--EK--RKLINYRDMAN-

>NF-YB_Q55PV5_CRYNE#106#166 | 61 | Q55PV5; | PD002982 171 bp

--------------FPA--AR---------VKKIVK---ADRDI-----------D----

------------------------------------------------------IMSSEA

VFMVS--------VAAEYFIKHFMEEGYTKARL--EK--RKLINYRDMAN-

>NF-YB_Q4P042_USTMA#31#92 | 62 | Q4P042; | PD002982 171 bp

--------------FPT--AR---------VARIIK---ADRDV-----------D----

------------------------------------------------------ICSKEA

TFLIS--------VATEIFLKRLTDEAYTNAKL--DK--RKNVLYKDLSRA

>NF-YB_Q6CI01_YARLI#54#115 | 62 | Q6CI01; | PD002982 171 bp

--------------LPL--AR---------TKRIVN---QDDDV-----------S----

------------------------------------------------------LVSVAA

YAAIN--------AATQDFVRYLSEQAGLMARM--DQ--RKTLAYKDVAEA

>NF-YB_Q5VRY6_ORYSA#119#180 | 62 | Q5VRY6; | PD002982 171 bp

--------------LPL--GR---------VKRIIR---VDRDI-----------K----

------------------------------------------------------KVTNEA

ALLIA--------AATELFVGSLAAGAHRAASR--RG--RRAVRAVHVRAA

>NF-YB_Q5EAR4_BRARE#14#75 | 62 | Q5EAR4; | PD002982 171 bp

------------ISLPI--SR---------VRLIMK---SSPDV-----------S----

------------------------------------------------------CINQDA

LFLTT--------KATELFVQHLALSSYENGPS--KD--TNTLSYSDLA--

>NF-YB_CHRC1_HUMAN#18#79 | 62 | Q9NRG0; | PD002982 171 bp

-------------SLPL--SR---------IRVIMK---SSPEV-----------S----

------------------------------------------------------SINQEA

LVLTA--------KATELFVQCLATYSYRHGSG--KE--KKVLTYSDLAN-

>NF-YB_CHRC1_MOUSE#18#78 | 61 | Q9JKP8; | PD002982 171 bp

-------------SLPL--SR---------IRVIMK---SSPEV-----------S----

------------------------------------------------------SINQEA

LVLTA--------KATELFVQYLATCSYRHGSG--KA--KKALTYSDLA--

>NF-YB_Q5ZKR0_CHICK#14#59 | 46 | Q5ZKR0; | PD002982 171 bp

-------------SLPL--SR---------IRVIMK---SSPEV-----------S----

------------------------------------------------------SINQDA

LFLTA--------KATELFVQYLASYSYKH---------------------

>NF-YB_Q9V452_DROME#19#61 | 43 | Q9V452; | PD002982 171 bp

-------------FLPL--SR---------VRTIMK---SSMDT-----------G----

------------------------------------------------------LITNEV

LFLMT--------KCTELFVRHLAGAA------------------------

>NF-YB_Q9LN09_ARATH#110#168 | 59 | Q9LN09; | PD002982 171 bp

--------------FPM--NR---------IRRIMR---SDNSA-----------P----

------------------------------------------------------QIMQDA

VFLVN--------KATEMFIERFSEEAYDSSVK--DK--KKFIHYKHL---

>NF-YB_Q851X6_ORYSA#205#259 | 55 | Q851X6; | PD002982 171 bp

-------------SFPM--AR---------VRQIMR---AEDAT-----------I----

------------------------------------------------------RPSNEA

VFLIN--------KATEIFLKRFADDAYRNALK--DR--KKSI--------

>NF-YB_Q9FHS0_ARATH#12#69 | 58 | Q9FHS0; | PD002982 171 bp

--------------FPI--GR---------VKKIMK---LDKDI-----------N----

------------------------------------------------------KINSEA

LHVIT--------YSTELFLHFLAEKSAVVTAE--KK--RKTVNLDH----

>NF-YB_Q6BP66_DEBHA#53#114 | 62 | Q6BP66; | PD002982 171 bp

--------------FPA--AR---------IKKIMQ---SDEDI-----------G----

------------------------------------------------------KVAQAT

PVIVG--------RALEIFMANLVEASIIEAKK--AG--VRRIGASHIRSA

>NF-YB_Q5AF72_CANAL#63#124 | 62 | Q5AF72; | PD002982 171 bp

--------------FPA--AR---------IKKIMQ---SDEEI-----------G----

------------------------------------------------------KVAQAT

PVIVG--------RALEIFMANLVEVSLLEAKK--QG--VKRITASHVKSA

>NF-YB_DPB3_YARLI#1#73 | 73 | Q6C6M5; | PD002982 171 bp

---MSEKPREIKTRFPV--AR---------IKKLMQ---SDDDI-----------G----

------------------------------------------------------KVAQAT

PTAVA--------KALELFMISLIEETCNQARM--RN--SKRVSPSHLKQA

>NF-YB_Q5AZD7_EMENI#190#251 | 62 | Q5AZD7; | PD002982 171 bp

--------------FPV--AR---------IKRIMQ---ADEDV-----------G----

------------------------------------------------------KVAQVT

PIAVS--------KALELFMISLVTKAAKEAKD--RN--SKRVTASHLKQA

>NF-YB_Q4X095_ASPFU#134#195 | 62 | Q4X095; | PD002982 171 bp

--------------FPV--AR---------IKRIMQ---ADEDV-----------G----

------------------------------------------------------KVAQVT

PIAVS--------KALELFMISLVTKAAKEARD--RN--SKRVTATHLKQA

>NF-YB_Q7RW27_NEUCR#203#259 | 57 | Q7RW27; | PD002982 171 bp

--------------FPT--AR---------IKRIMQ---ADEEV-----------G----

------------------------------------------------------KVAQQT

PIAVG--------KALELFMVQLVTKSADIARE--RN--SKRVSAQ-----

>NF-YB_Q4IBB0_GIBZE#215#271 | 57 | Q4IBB0; | PD002982 171 bp

--------------FPT--AR---------IKRIMQ---ADEEV-----------G----

------------------------------------------------------KVAQQT

PIAVG--------KALELFMIQLVTKSADVAKD--KG--SKRVTAS-----

>NF-YB_DPB3_SCHPO#24#85 | 62 | Q10315; | PD002982 171 bp

--------------FPV--AR---------IKKIMQ---ADQDV-----------G----

------------------------------------------------------KVAQVT

PVIMS--------KALELFMQSIIQESCKQTRL--HQ--AKRVTVSHLKHA

>NF-YB_Q4PG57_USTMA#10#77 | 68 | Q4PG57; | PD002982 171 bp

--------------FPV--AR---------IKKIMQ---ADEDV-----------G----

------------------------------------------------------KVAQAT

PVLISIWNLGLTAKALELFMASIVEETVKETRS--RG--AKKMTPYHVK--

>NF-YB_Q6CTK6_KLULA#70#129 | 60 | Q6CTK6; | PD002982 171 bp

--------------FPP--AK---------IKKIMQ---TDEDI-----------G----

------------------------------------------------------KVSQAT

PVITG--------RSLEFFIALLVDKSSKVARD--QG--SKRISADIMK--

>NF-YB_Q74ZP2_ASHGO#24#83 | 60 | Q74ZP2; | PD002982 171 bp

--------------FPP--AK---------IKKIMQ---TDEDI-----------G----

------------------------------------------------------KVSQAT

PVITG--------RSLEFFIAMLVDKSSQMAKE--QG--SKRISGDIMK--

>NF-YB_NCB1_YEAST#53#112 | 60 | P40096; | PD002982 171 bp

--------------FPP--AK---------VKKIMQ---TDEDI-----------G----

------------------------------------------------------KVSQAT

PVIAG--------RSLEFFIALLVKKSGEMARG--QG--TKRITAEILK--

>NF-YB_Q6FQ70_CANGA#50#108 | 59 | Q6FQ70; | PD002982 171 bp

--------------FPP--AK---------VKKIMQ---SDEDI-----------G----

------------------------------------------------------KVSQAT

PVITG--------RSLEFFMALLVKKSGDMARQ--RG--MKRITADVL---

>NF-YB_Q4FJW2_MOUSE#12#72 | 61 | Q4FJW2; | PD002982 171 bp

--------------FPP--AR---------IKKIMQ---TDEEI-----------G----

------------------------------------------------------KVAAAV

PVIIS--------RALELFLESLLKKACQVTQS--RN--AKTMTTSHLKQ-

>NF-YB_Q2YDP3_BOVIN#12#72 | 61 | Q2YDP3; | PD002982 171 bp

--------------FPP--AR---------IKKIMQ---TDEEI-----------G----

------------------------------------------------------KVAAAV

PVIIS--------RALELFLESLLKKACQVTQS--RN--AKTMTTSHLKQ-

>NF-YB_DRAP1_MOUSE#11#71 | 61 | Q9D6N5; | PD002982 171 bp

--------------FPP--AR---------IKKIMQ---TDEEI-----------G----

------------------------------------------------------KVAAAV

PVIIS--------RALELFLESLLKKACQVTQS--RN--AKTMTTSHLKQ-

>NF-YB_DRAP1_HUMAN#11#71 | 61 | Q14919; | PD002982 171 bp

--------------FPP--AR---------IKKIMQ---TDEEI-----------G----

------------------------------------------------------KVAAAV

PVIIS--------RALELFLESLLKKACQVTQS--RN--AKTMTTSHLKQ-

>NF-YB_Q5HZC2_XENLA#12#72 | 61 | Q5HZC2; | PD002982 171 bp

--------------FPP--AR---------IKKIMQ---TDEEI-----------G----

------------------------------------------------------KVAAAV

PVIIS--------RALELFLESLLKKACHVTQS--RN--AKTMTTSHLKQ-

>NF-YB_Q7ZVI1_BRARE#12#72 | 61 | Q7ZVI1; | PD002982 171 bp

--------------FPP--AR---------IKKIMQ---TDEEI-----------G----

------------------------------------------------------KVAAAV

PVIIS--------RALELFLESLLTKACDVTQS--RN--AKTMTTSHLKQ-

>NF-YB_Q5HZ78_XENLA#12#72 | 61 | Q5HZ78; | PD002982 171 bp

--------------FPP--AR---------IKKIMQ---TDEEI-----------G----

------------------------------------------------------KVAAPV

PVIIS--------RALELFLESLLKKTCHVTQS--RS--AKTMTTSHLKQ-

>NF-YB_Q9W2E3_DROME#12#69 | 58 | Q9W2E3; | PD002982 171 bp

--------------FPA--GR---------IKKIMQ---SDEEI-----------G----

------------------------------------------------------KVAQAV

PVIIS--------RTLELFVESLLTKTLRITNA--RN--AKTLSPSH----

>NF-YB_Q9GSP1_DROME#12#69 | 58 | Q9GSP1; | PD002982 171 bp

--------------FPA--GR---------IKKIMQ---SDEEI-----------G----

------------------------------------------------------KVAQAV

PVIIS--------RTLELFVESLLTKTLRITNA--RN--AKTLSPSH----

>NF-YB_Q65X80_ORYSA#9#69 | 61 | Q65X80; | PD002982 171 bp

--------------FPA--PR---------IKKIMQ---ADEDV-----------G----

------------------------------------------------------KIALAV

PVLVS--------KALELFLQDLCNRTYDITVQ--RG--VKTLSSSHLKQ-

>NF-YB_Q8W0W7_ORYSA#9#63 | 55 | Q8W0W7; | PD002982 171 bp

--------------FPA--AR---------IKKIMQ---ADEDV-----------G----

------------------------------------------------------KIALAV

PVLVS--------RALELFLQDLIDRTYEITLQ--SG--AKTLN-------

>NF-YB_Q8W0W6_MAIZE#9#63 | 55 | Q8W0W6; | PD002982 171 bp

--------------FPA--AR---------IKKIMQ---ADEDV-----------G----

------------------------------------------------------KIALAV

PVLVS--------RSLELFLQDLIDRTYEITLQ--SG--AKTLN-------

>NF-YB_Q9LHG0_ARATH#9#63 | 55 | Q9LHG0; | PD002982 171 bp

--------------FPA--AR---------IKKIMQ---ADEDV-----------G----

------------------------------------------------------KIALAV

PVLVS--------KSLELFLQDLCDRTYEITLE--RG--AKTVS-------

>NF-YB_Q9C7A7_ARATH#16#67 | 52 | Q9C7A7; | PD002982 171 bp

-------------------AR---------IKKIMQ---ADEDV-----------G----

------------------------------------------------------KIALAV

PVLVS--------KSLELFLQDLCDRTYEITLE--RG--AKTVS-------

>NF-YB_Q54DA1_DICDI#9#67 | 59 | Q54DA1; | PD002982 171 bp

--------------FPM--AR---------IKKIMQ---KDEEV-----------G----

------------------------------------------------------KIASAT

PILIS--------QCLELFMADLVMKTCKITQA--KK--GKVISVNHL---

>NF-YB_Q518K3_ENTHI#8#66 | 59 | Q518K3; | PD002982 171 bp

-------------LLPA--AR---------VKRIMQ---EDEDV-----------G----

------------------------------------------------------KMSGNV

PMVIA--------RATELFLVDLIKKTNTVAEE--KK--SKSVNLSH----

>NF-YB_Q7KWN0_DICDI#72#136 | 65 | Q7KWN0; | PD002982 171 bp

-------RVEGDIQLPV--AR---------IKRIMR---CDKDV-----------K----

------------------------------------------------------IISSDA

VMLVA--------KSTEMFLDYLVKEAYKSSGK------KKTLQYKDLA--

>NF-YB_Q7PI02_ANOGA#136#193 | 58 | Q7PI02; | PD002982 171 bp

-----ESPEERLAQFPF--AR---------IKQMMK---LDPEV-----------G----

------------------------------------------------------IVSAEA

IFLVT--------KAAELFLQTLAKDTSFHTNR--H---------------

>NF-YB_Q8W099_ORYSA#45#105 | 61 | Q8W099; | PD002982 171 bp

---------------PM--AR---------LKKIVS---SQKGN-----------M----

------------------------------------------------------MMTFDM

PAFLS--------KMCELFVQELAVRAWASAQS--HN--RCIILDTDIAKA

>NF-YB_Q93VF0_ORYSA#45#105 | 61 | Q93VF0; | PD002982 171 bp

---------------PM--AR---------LKKIAS---SQKGN-----------M----

------------------------------------------------------MMSFDM

PAFLS--------KMCELFVQELAVRAWASAQS--HN--RCIILDTDIAEA

>NF-YB_Q5Z8C9_ORYSA#45#105 | 61 | Q5Z8C9; | PD002982 171 bp

---------------PM--AR---------LKKIVS---SQKGN-----------M----

------------------------------------------------------MMTFDM

PAFLS--------KMCELFVQELAARAWACAQS--HN--RCIILDMDIAEA

>NF-YB_Q8LMX6_ORYSA#49#114 | 66 | Q8LMX6; | PD002982 171 bp

----------EELILPI--SR---------VKNIIH---AKEGG-----------M----

------------------------------------------------------MLSADT

PAFVT--------KLCELFVQELILRAWVCANS--HN--REIILGTDIAEA

>NF-YB_Q5CQK9_CRYPV#151#212 | 62 | Q5CQK9; | PD002982 171 bp

------------RCLPH--TK---------IKKIIK---CSGAV-----------NH---

------------------------------------------------------MIGSEV

PALLA--------IACELFVRDLTSFSWNFTRR--AK--RRTVQVQDI---

>NF-YB_Q7QXC2_GIALA#13#83 | 71 | Q7QXC2; | PD002982 171 bp

---MDTHSKDAVRRLPI--SR---------VRAICR---TVPTI-----------T----

------------------------------------------------------LLSAEA

PLLIS--------RLAELFIADVTNQSYQMAIR--SN--ATTVTEDDVA--

>NF-YB_Q519M1_ENTHI#120#195 | 76 | Q519M1; | PD002982 171 bp

MSVESENYNFKEKPFPP--AR---------IRKLTK---INIDN-----------K----

------------------------------------------------------QLKTET

VEILS--------RACELFIKDLTTRAGYITSY--SK--RKVIKKDDIVKA

>NF-YB_Q5CJM8_CRYHO#10#63 | 54 | Q5CJM8; | PD002982 171 bp

--------------FPD--ST---------ISRISK-------------------SVL--

--------------------------------------------------SNNSRLSKDA

CKIIN--------KCATLFSIYLASLSCSSKD---GK--KSTV--------

Family: NF-YC

>NF-YC_CBFC_HUMAN#44#117 | 74 | Q13952; | PD003659 135 bp

-----------------PLARIKKIMKLDE--------------DV--K---MISAEAPV

LFAKAAQIFITELTLRAWIHTEDNK--RR------TLQ------RNDIAMAITK-FDQFD

FLIDI----------

>NF-YC_CBFC_RAT#44#117 | 74 | Q62725; | PD003659 135 bp

-----------------PLARIKKIMKLDE--------------DV--K---MISAEAPV

LFAKAAQIFITELTLRAWIHTEDNK--RR------TLQ------RNDIAMAITK-FDQFD

FLIDI----------

>NF-YC_O73745_XENLA#42#115 | 74 | O73745; | PD003659 135 bp

-----------------PLARIKKIMKLDE--------------DV--K---MISAEAPV

LFAKAAQIFITELTLRAWIHTEDNK--RR------TLQ------RNDIAMAITK-FDQFD

FLIDI----------

>NF-YC_Q6DCR1_XENLA#42#115 | 74 | Q6DCR1; | PD003659 135 bp

-----------------PLARIKKIMKLDE--------------DV--K---MISAEAPV

LFAKAAQIFITELTLRAWIHTEDNK--RR------TLQ------RNDIAMAITK-FDQFD

FLIDI----------

>NF-YC_Q6P0F1_BRARE#41#114 | 74 | Q6P0F1; | PD003659 135 bp

-----------------PLARIKKIMKLDE--------------DV--K---MISAEAPV

LFAKAAQIFITELTLRAWIHTEDNK--RR------TLQ------RNDIAMAITK-FDQFD

FLIDI----------

>NF-YC_Q6P4L4_XENTR#43#116 | 74 | Q6P4L4; | PD003659 135 bp

-----------------PLARIKKIMKLDE--------------DV--K---MISAEAPV

LFAKAAQIFITELTLRAWIHTEDNK--RR------TLQ------RNDIAMAITK-FDQFD

FLIDI----------

>NF-YC_Q7TPS7_MOUSE#44#117 | 74 | Q7TPS7; | PD003659 135 bp

-----------------PLARIKKIMKLDE--------------DV--K---MISAEAPV

LFAKAAQIFITELTLRAWIHTEDNK--RR------TLQ------RNDIAMAITK-FDQFD

FLIDI----------

>NF-YC_Q7ZVY3_BRARE#42#115 | 74 | Q7ZVY3; | PD003659 135 bp

-----------------PLARIKKIMKLDE--------------DV--K---MISAEAPV

LFAKAAQIFITELTLRAWIHTEDNK--RR------TLQ------RNDIAMAITK-FDQFD

FLIDI----------

>NF-YC_CBFC_MOUSE#44#117 | 74 | P70353; | PD003659 135 bp

-----------------PLARIKKIMKLDE--------------DV--K---MISAEAPV

LFAKGAQIFITELTLRAWIRTEDNK--RR------PLQ------RNDIAMAITK-FDQFD

FLIDI----------

>NF-YC_HAP5_YEAST#147#234 | 88 | Q02516; | PD003659 135 bp

PGSEHQDDFKSH---SLPFARIRKVMKTDE--------------DV--K---MISAEAPI

IFAKACEIFITELTMRAWCVAERNK--RR------TLQ------KADIAEALQK-SDMFD

FLIDV----------

>NF-YC_Q6FM95_EEEEE#101#188 | 88 | Q6FM95; | PD003659 135 bp

PGSEYQDDFKSH---SLPFARIRRVMKTDE--------------EV--K---MISAEAPI

IFAKACEVFITELTMRAWCVAEKHK--RR------TLQ------KADIAEALQM-SDMFD

FLIDI----------

>NF-YC_Q6CY26_EEEEE#77#150 | 74 | Q6CY26; | PD003659 135 bp

-----------------PLARIKKVMKTDE--------------EV--R---MISGEAPI

LFAKACEIFITELTMRAWCVAEENK--RR------TLQ------KQDIADALQK-SDMFD

FLIDI----------

>NF-YC_Q758Y6_ASHGO#66#139 | 74 | Q758Y6; | PD003659 135 bp

-----------------PLARIKKVMKTDE--------------DV--K---MISAEAPI

LFAKACEIFITELTMRAWCIAEENK--RR------TLQ------KQDIAQALQK-SDMFD

FLIDI----------

>NF-YC_O04033_ARATH#54#132 | 79 | O04033; | PD003659 135 bp

---------KNH---SLPLARIKKIMKADE--------------DV--R---MISAEAPV

VFARACEMFILELTLRSWNHTEENK--RR------TLQ------KNDIAAAVTR-TDIFD

FLVDI----------

>NF-YC_Q9ZVL3_ARATH#67#145 | 79 | Q9ZVL3; | PD003659 135 bp

---------KNH---SLPLARIKKIMKADE--------------DV--R---MISAEAPV

VFARACEMFILELTLRSWNHTEENK--RR------TLQ------KNDIAAAVTR-TDIFD

FLVDI----------

>NF-YC_Q8L4B2_ARATH#77#155 | 79 | Q8L4B2; | PD003659 135 bp

---------KNH---SLPLARIKKIMKADE--------------DV--R---MISAEAPV

VFARACEMFILELTLRSWNHTEENK--RR------TLQ------KNDIAAAVTR-TDIFD

FLVDI----------

>NF-YC_Q9SNZ0_ARATH#77#155 | 79 | Q9SNZ0; | PD003659 135 bp

---------KKH---SLPLARIKKIMKADE--------------DV--R---MISAEAPV

VFARACEMFILELTLRSWNHTEENK--RR------TLQ------KNDIAAAVTR-TDIFD

FLVDI----------

>NF-YC_Q6H707_EEEEE#102#180 | 79 | Q6H707; | PD003659 135 bp

---------KNH---SLPLARIKKIMKADE--------------DV--R---MISAEAPV

VFAKACEVFILELTLRSWMHTEENK--RR------TLQ------KNDIAAAITR-TDIYD

FLVDI----------

>NF-YC_O23636_ARATH#11#89 | 79 | O23636; | PD003659 135 bp

---------KNH---TLPLARIKKIMKADE--------------DV--R---MISAEAPV

IFAKACEMFILELTLRAWIHTEENK--RR------TLQ------KNDIAAAISR-TDVFD

FLVDI----------

>NF-YC_Q9SGU4_ARATH#11#89 | 79 | Q9SGU4; | PD003659 135 bp

---------KNH---TLPLARIKKIMKADE--------------DV--R---MISAEAPV

IFAKACEMFILELTLRAWIHTEENK--RR------TLQ------KNDIAAAISR-TDVFD

FLVDI----------

>NF-YC_Q8VY66_ARATH#73#151 | 79 | Q8VY66; | PD003659 135 bp

---------KNH---TLPLARIKKIMKADE--------------DV--R---MISAEAPV

IFAKACEMFILELTLRAWIHTEENK--RR------TLQ------KNDIAAAISR-TDVFD

FLVDI----------

>NF-YC_Q8LCG7_ARATH#73#150 | 78 | Q8LCG7; | PD003659 135 bp

---------KNH---TLPLARIKKIMKADE--------------DV--R---MISAEAPV

IFAKACEMFILELTLRAWIHTEENK--RR------TLQ------KNDIAAAISR-TDV-D

FLVDI----------

>NF-YC_Q76FX2_DAUCA#79#157 | 79 | Q76FX2; | PD003659 135 bp

---------KNH---SLPLARIKKIMKADE--------------DV--R---MISSEAPV

VFAKACEMFIMDLTMRSWSHTEENK--RR------TLQ------KNDIAAAVSR-TDVFD

FLVDI----------

>NF-YC_Q76FX1_DAUCA#98#176 | 79 | Q76FX1; | PD003659 135 bp

---------KNH---SLPLARIKKIMKADE--------------DV--R---MISSEAPV

IFAKACEMFILELTMRSWLLTEENK--RR------TLQ------KNDIAAAISR-TDIFD

FLVDI----------

>NF-YC_Q9FMV5_ARATH#75#163 | 89 | Q9FMV5; | PD003659 135 bp

---------KNH---QLPLARIKKIMKADE--------------DV--R---MISAEAPI

LFAKACELFILELTIRSWLHAEENK--RR------TLQ------KNDIAAAITR-TDIFD

FLVDIVPREEIKEEE

>NF-YC_Q9SMP0_ARATH#62#140 | 79 | Q9SMP0; | PD003659 135 bp

---------KNH---QLPLARIKKIMKADE--------------DV--R---MISAEAPI

LFAKACELFILELTIRSWLHAEENK--RR------TLQ------KNDIAAAITR-TDIFD

FLVDI----------

>NF-YC_Q9XE33_EEEEE#87#165 | 79 | Q9XE33; | PD003659 135 bp

---------KNH---NLPLARIKKIMKADE--------------DV--R---MIAAEAPV

VFARACEMFILELTHRGWAHAEENK--RR------TLQ------KSDIAAAIAR-TEVFD

FLVDI----------

>NF-YC_Q6BS91_EEEEE#101#179 | 79 | Q6BS91; | PD003659 135 bp

---------KNH---QLPLARIKKVMKTDE--------------DV--R---MISAEAPI

LFAKGCDVFITELTMRAWIHAEENK--RR------TLQ------KSDIAAALTK-SDMFD

FLIDI----------

>NF-YC_Q6CFV6_EEEEE#93#166 | 74 | Q6CFV6; | PD003659 135 bp

-----------------PLARIKKVMKADE--------------DV--K---MISAEAPI

LFAKGCDIFITELSMRAWIHAEEHK--RR------TLQ------RSDIASALQR-SDMFD

FLIDI----------

>NF-YC_O13381_NEUCR#84#167 | 84 | O13381; | PD003659 135 bp

---------KLH---QLPLARIKKVMKADP--------------EV--K---MISAEAPI

LFAKGCDIFITELTMRAWIHAEENK--RR------TLQ------RSDIASALSK-SDMFD

FLIDIVPREE-----

>NF-YC_Q7RVQ7_NEUCR#75#158 | 84 | Q7RVQ7; | PD003659 135 bp

---------KLH---QLPLARIKKVMKADP--------------EV--K---MISAEAPI

LFAKGCDIFITELTMRAWIHAEENK--RR------TLQ------RSDIASALSK-SDMFD

FLIDIVPREE-----

>NF-YC_Q96WN9_TRIRE#84#167 | 84 | Q96WN9; | PD003659 135 bp

---------KIH---QLPLARIKKVMKADP--------------EV--K---MISAEAPI

LFAKGCDIFITELTMRAWIHAEENK--RR------TLQ------RSDIASALAK-SDMFD

FLIDIVPREE-----

>NF-YC_O59849_ASPOR#83#166 | 84 | O59849; | PD003659 135 bp

---------KIH---QLPLARIKKVMKADP--------------EV--K---MISAEAPI

LFAKGCDIFITELTMRAWIHAEDNK--RR------TLQ------RSDIAAALSK-SDMFD

FLIDIVPREE-----

>NF-YC_P87092_EMENI#83#166 | 84 | P87092; | PD003659 135 bp

---------KIH---QLPLARIKKVMKADP--------------EV--K---MISAEAPI

LFAKGCDVFITELTMRAWIHAEDNK--RR------TLQ------RSDIAAALSK-SDMFD

FLIDIVPREE-----

>NF-YC_Q7Z8M0_ASPNG#83#166 | 84 | Q7Z8M0; | PD003659 135 bp

---------KIH---QLPLARIKKVMKADP--------------EV--K---MISAEAPI

LFAKGCDIFITELTMRAWTHAEDNK--RR------TLQ------RSDIAAALSK-SDMFD

FLIVIVPREE-----

>NF-YC_Q9W3V9_DROME#155#228 | 74 | Q9W3V9; | PD003659 135 bp

-----------------PLARIKKIMKLDE--------------NA--K---MIAGEAPL

LFAKACEYFIQELTMHAWVHTEESR--RR------TLQ------RSDIAQAIAN-YDQFD

FLIDI----------

>NF-YC_Q6ZKK7_EEEEE#67#145 | 79 | Q6ZKK7; | PD003659 135 bp

---------KLP---NLPLARIKKIMKADE--------------DV--K---MIAGEAPA

LFAKACEMFILDMTLRSWQHTEEGR--RR------TLQ------RSDVEAVIKK-TDIFD

FLVDI----------

>NF-YC_Q9FGP7_ARATH#51#129 | 79 | Q9FGP7; | PD003659 135 bp

---------KNR---QLPLARIKKIMKADP--------------DV--H---MVSAEAPI

IFAKACEMFIVDLTMRSWLKAEENK--RH------TLQ------KSDISNAVAS-SFTYD

FLLDV----------

>NF-YC_PHP5_SCHPO#108#185 | 78 | P79007; | PD003659 135 bp

---------------HLPLARIKKVMKTDD--------------DVKNK---MISAEAPF

LFAKGSEIFIAELTMRAWLHAKKNQ--RR------TLQ------RSDIANAVSK-SEMYD

FLIDI----------

>NF-YC_Q8SSZ4_DICDI#267#345 | 79 | Q8SSZ4; | PD003659 135 bp

---------KTH---ELPLARIKKIMKSDK--------------DV--N---KISSEAPI

LFAKACEILILEMTHRSWVHTEMNK--RR------TLQ------RTDIINSLSR-CETFD

FLIDM----------

>NF-YC_Q9FGP6_ARATH#33#110 | 78 | Q9FGP6; | PD003659 135 bp

---------KNH---EFPISRIKRIMKFDP--------------DV--S---MIAAEAPN

LLSKACEMFVMDLTMRSWLHAQESN--RL------TIR------KSDVDAVVSQ-TVIFD

FLRD-----------

>NF-YC_Q7RRA6_PLAYO#27#118 | 92 | Q7RRA6; | PD003659 135 bp

-----PEELKTH---QLPISRIKKIMKEDD--------------KI--KNSQMISADTPV

LLAKACELFIMEFTKYAWKYTEENK--RR------TLQ------RQDVIAAACR-KDIFD

FLIDLISIEDR----

>NF-YC_Q8IL74_PLAF7#26#118 | 93 | Q8IL74; | PD003659 135 bp

----SPEDLKIH---NLPISRIKKIMKEDD--------------EI--KSNQMVSADTPV

LLAKACELFIMELTSNAWKYTEEGK--RR------TLQ------RQDVVSAACK-KDTFD

FLIDLIPLEDR----

>NF-YC_Q7XPV7_EEEEE#24#112 | 89 | Q7XPV7; | PD003659 135 bp

----------------LPLARIKKIMKRSAGDSSVVDGGGGGGGGA--R---MISGEAPV

VFSKACELFIAELTRRAWAATLEGK--RR------TVH------KEDVAAAVQN-TDLFD

FLVDV----------

>NF-YC_Q8SRY9_EEEEE#39#115 | 77 | Q8SRY9; | PD003659 135 bp

----------------LPLARIKRLMKIEE--------------GV--R---MVASEVPV

LFSMITEKFIEELTLRAWINTEENK--RR------ILQ------KSDLTAAVKT-SEMFD

FLVYIVP--------

>NF-YC_O17072_CAEEL#111#185 | 75 | O17072; | PD003659 135 bp

-----------------PMARVKKIMRIDD--------------DV--R-NFMIASDAPI

FMAQAAEFFIEEMTAMGWQYVSEAR--RR------ILQ------KADIASAVQK-SDQFD

FLID-----------

>NF-YC_Q9FGP8_ARATH#64#144 | 81 | Q9FGP8; | PD003659 135 bp

----------------FPLTRIKKIMKSNP--------------EV--N---MVTAEAPV

LISKACEMLILDLTMRSWLHTVEGG--RQ------TLKRSDTLTRSDISAATTR-SFKFT

FLGDV----------

>NF-YC_Q9FF42_ARATH#66#145 | 80 | Q9FF42; | PD003659 135 bp

--------------THLPLSRVRKILKSDP--------------EV--K---KISCDVPA

LFSKACEYFILEVTLRAWMHTQSCT--RE------TIR------RCDIFQAVKN-SGTYD

FLIDRVPF-------

>NF-YC_Q8LMX6_EEEEE#53#124 | 72 | Q8LMX6; | PD003659 135 bp

----------------LPISRVKNIIHAKE--------------GG--M---MLSADTPA

FVTKLCELFVQELILRAWVCANSHN--RE------IIL------GTDIAEAITT-TESYH

FL-------------

>NF-YC_Q8W099_EEEEE#45#118 | 74 | Q8W099; | PD003659 135 bp

-----------------PMARLKKIVSSQK--------------GN--M---MMTFDMPA

FLSKMCELFVQELAVRAWASAQSHN--RC------IIL------DTDIAKAIAS-TESYD

FLVDI----------

>NF-YC_Q93VF0_EEEEE#45#118 | 74 | Q93VF0; | PD003659 135 bp

-----------------PMARLKKIASSQK--------------GN--M---MMSFDMPA

FLSKMCELFVQELAVRAWASAQSHN--RC------IIL------DTDIAEAIAS-TESYD

FLVDI----------

>NF-YC_DPE4_HUMAN#37#112 | 76 | Q9NR33; | PD003659 135 bp

------------RLSRLPLARVKALVKADP--------------DV--T---LAGQEAIF

ILARAAELFVETIAKDAYCCAQQGK--RK------TLQ------RRDLDNAIEA-VDEFA

FL-------------

>NF-YC_DPE4_MOUSE#38#113 | 76 | Q9CQ36; | PD003659 135 bp

------------RLSRLPLARVKALVKADP--------------DV--T---LAGQEAIF

ILARAAELFVETIAKDAYCCAQQGK--RK------TLQ------RRDLDNAIEA-VDEFA

FL-------------

>NF-YC_Q8MSF2_DROME#52#127 | 76 | Q8MSF2; | PD003659 135 bp

------------KMTQLPLARIRNIMKLDP--------------DL--H---MANNEAVF

IVAKAVELFIASLSRESYTYTAQSK--KK------TIQ------KRDVDMAISA-VDSLL

FL-------------

>NF-YC_Q9W256_DROME#74#149 | 76 | Q9W256; | PD003659 135 bp

------------KMTQLPLARIRNIMKLDP--------------DL--H---MANNEAVF

IVAKAVELFIASLSRESYTYTAQSK--KK------TIQ------KRDVDMAISA-VDSLL

FL-------------

>NF-YC_Q7PI02_EEEEE#135#219 | 85 | Q7PI02; | PD003659 135 bp

------KESPEERLAQFPFARIKQMMKLDP--------------EV--G---IVSAEAIF

LVTKAAELFLQTLAKDTSFHTVASK--KK------TMS------KRDVETAIDN-VDSLV

FLEGM----------

>NF-YC_Q9NAC5_CAEEL#26#109 | 84 | Q9NAC5; | PD003659 135 bp

------EHVEELVRSQLPLGRVKKVVRMNP--------------DV--E---MLNNEALQ

LMAKAAELFIKELSNAANQNAALEK--RK------TVQ------TKDIDKAIKK-TWAFA

FLED-----------

>NF-YC_Q7KWN0_DICDI#63#151 | 89 | Q7KWN0; | PD003659 135 bp

EKKKSKRRTRVEGDIQLPVARIKRIMRCDK--------------DV--K---IISSDAVM

LVAKSTEMFLDYLVKEAYKSS--GK--KK------TLQ------YKDLASTIKG-VDNLD

FLSEI----------

>NF-YC_Q9LN09_ARATH#104#183 | 80 | Q9LN09; | PD003659 135 bp

----------EDAKIKFPMNRIRRIMRSDN--------------SA--P---QIMQDAVF

LVNKATEMFIERFSEEAYDSSVKDK--KK------FIH------YKHLSSVVSN-DQRYE

FLAD-----------

>NF-YC_Q851X6_EEEEE#206#257 | 52 | Q851X6; | PD003659 135 bp

----------------FPMARVRQIMRAED--------------AT--I---RPSNEAVF

LINKATEIFLKRFADDAYRNALKDR--KK-------------------------------

---------------

>NF-YC_DPB3_YEAST#12#95 | 84 | P27344; | PD003659 135 bp

----------------FPISKVKKIAKCDP--------------EY--V---ITSNVAIS

ATAFAAELFVQNLVEESLVLAQLNS--KG--KTSLRLS------LNSIEECVEK-RDNFR

FLEDAIKQLK-----

>NF-YC_Q6FXJ8_EEEEE#12#102 | 91 | Q6FXJ8; | PD003659 135 bp

------QQKIRDRTPKLPISKVKRIGKVDP--------------ES--I---LTSNMAYV

ATAFATELFVQSFVEQALFGAQLRR-GKK--KAGLRLT------NDALVECVRN-RDDYI

FLEDVV---------

>NF-YC_Q6CLM5_EEEEE#5#90 | 86 | Q6CLM5; | PD003659 135 bp

----------KSKLPRIPISKCKKIARTDP--------------EY--I---LTSQAAFA

ATAFTTELFIQMLAEETCSLAQIHK--QT---KTLRLN------YEDLSTAIRN-LDKFQ

FLSDVVP--------

>NF-YC_Q758B1_ASHGO#12#95 | 84 | Q758B1; | PD003659 135 bp

---------------RLPISKCKRIAKTDP--------------DY--I---MTTQAAYI

ATAFATELFVQAISEDAMAQAQLDG--RRAGGRAARLT------YNDLARSVAR-EERYA

FLADVIP--------

>NF-YC_Q6BX14_EEEEE#92#165 | 74 | Q6BX14; | PD003659 135 bp

----------------LPLSKIKKIFKMDP--------------DY--L---AASQSAVY

ATGLATELFIQYFTEQSLVLAKMDK--RK------KLQ------YKDFSNAVAS-QDSLN

FLSD-----------

>NF-YC_Q6CI01_EEEEE#54#130 | 77 | Q6CI01; | PD003659 135 bp

----------------LPLARTKRIVNQDD--------------DV--S---LVSVAAYA

AINAATQDFVRYLSEQAGLMARMDQ--RK------TLA------YKDVAEAIAK-NPKLE

FLQDIIP--------

>NF-YC_Q7S9P3_NEUCR#18#98 | 81 | Q7S9P3; | PD003659 135 bp

--------------TQLPLTRVKKIIAVDP--------------DI--T---VCSNNAAF

VITLATEMFIQHLASEAQNMAKAERKPRR------NVQ------YKDVAAAVSH-HDNLE

FLEDVVP--------

>NF-YC_Q9FHS0_ARATH#12#86 | 75 | Q9FHS0; | PD003659 135 bp

----------------FPIGRVKKIMKLDK--------------DI--N---KINSEALH

VITYSTELFLHFLAEKSAVVTAEKK--RK------TVN------LDHLRIAVKRHQPTSD

FLLD-----------

>NF-YC_Q9LGS9_EEEEE#167#241 | 75 | Q9LGS9; | PD003659 135 bp

----------------LPLGRVKRIIRVDR--------------DI--K---KVTNEAAL

LIAAATELFVGSLAAGAHRAASRRG--RR------AVR------AVHVRAAAREHRPTAD

FLLD-----------

>NF-YC_CHR1_HUMAN#13#97 | 85 | Q9NRG0; | PD003659 135 bp

----------EQRLISLPLSRIRVIMKSSP--------------EV--S---SINQEALV

LTAKATELFVQCLATYSYRHGSGKE--KK------VLT------YSDLANTAQQ-SETFQ

FLADILPKK------

>NF-YC_CHR1_MOUSE#7#97 | 91 | Q9JKP8; | PD003659 135 bp

----GKEKCGDQRLVSLPLSRIRVIMKSSP--------------EV--S---SINQEALV

LTAKATELFVQYLATCSYRHGSGKA--KK------ALT------YSDLASTAED-SETLQ

FLADILPKK------

>NF-YC_Q9V452_DROME#20#99 | 80 | Q9V452; | PD003659 135 bp

----------------LPLSRVRTIMKSSM--------------DT--G---LITNEVLF

LMTKCTELFVRHLAGAAYTEEFGQR-PGE------ALK------YEHLSQVVNK-NKNLE

FLLQIVPQK------

>NF-YC_NCB1_YEAST#51#125 | 75 | P40096; | PD003659 135 bp

--------------THFPPAKVKKIMQTDE--------------DI--G---KVSQATPV

IAGRSLEFFIALLVKKSGEMARGQG--TK------RIT------AEILKKTILN-DEKFD

FLR------------

>NF-YC_Q6CTK6_EEEEE#68#144 | 77 | Q6CTK6; | PD003659 135 bp

--------------THFPPAKIKKIMQTDE--------------DI--G---KVSQATPV

ITGRSLEFFIALLVDKSSKVARDQG--SK------RIS------ADIMKKTILT-DEKFD

FLREV----------

>NF-YC_Q74ZP2_ASHGO#22#98 | 77 | Q74ZP2; | PD003659 135 bp

--------------THFPPAKIKKIMQTDE--------------DI--G---KVSQATPV

ITGRSLEFFIAMLVDKSSQMAKEQG--SK------RIS------GDIMKKTIMA-DEKFD

FLREI----------

>NF-YC_Q6FQ70_EEEEE#44#122 | 79 | Q6FQ70; | PD003659 135 bp

----------EKIKTHFPPAKVKKIMQSDE--------------DI--G---KVSQATPV

ITGRSLEFFMALLVKKSGDMARQRG--MK------RIT------ADVLRDTILE-DERFD

FLR------------

>NF-YC_Q7RW27_NEUCR#201#277 | 77 | Q7RW27; | PD003659 135 bp

--------------TKFPTARIKRIMQADE--------------EV--G---KVAQQTPI

AVGKALELFMVQLVTKSADIARERN--SK------RVS------AQMLKQVVES-DDQWD

FLREI----------

>NF-YC_Q6C6M5_EEEEE#3#86 | 84 | Q6C6M5; | PD003659 135 bp

-------EKPREIKTRFPVARIKKLMQSDD--------------DI--G---KVAQATPT

AVAKALELFMISLIEETCNQARMRN--SK------RVS------PSHLKQAVLE-TEQFD

FLQDI----------

>NF-YC_YD63_SCHPO#22#98 | 77 | Q10315; | PD003659 135 bp

--------------SRFPVARIKKIMQADQ--------------DV--G---KVAQVTPV

IMSKALELFMQSIIQESCKQTRLHQ--AK------RVT------VSHLKHAVQS-VEQFD

FLQDI----------

>NF-YC_Q6BP66_EEEEE#44#124 | 81 | Q6BP66; | PD003659 135 bp

-------ESFEKIKTHFPAARIKKIMQSDE--------------DI--G---KVAQATPV

IVGRALEIFMANLVEASIIEAKKAG--VR------RIG------ASHIRSAVEN-TEQFD

FL-------------

>NF-YC_Q13448_HUMAN#4#74 | 71 | Q13448; | PD003659 135 bp

--------------------RIKKIMQTDE--------------EI--G---KVAAAVPV

IISRALELFLESLLKKACQVTQSRN--AK------TMT------TSHLKQCIEL-EQQFD

FLKDL----------

>NF-YC_Q14919_HUMAN#3#86 | 84 | Q14919; | PD003659 135 bp

-------SKKKKYNARFPPARIKKIMQTDE--------------EI--G---KVAAAVPV

IISRALELFLESLLKKACQVTQSRN--AK------TMT------TSHLKQCIEL-EQQFD

FLKDL----------

>NF-YC_Q9D6N5_MOUSE#3#86 | 84 | Q9D6N5; | PD003659 135 bp

-------SKKKKYNARFPPARIKKIMQTDE--------------EI--G---KVAAAVPV

IISRALELFLESLLKKACQVTQSRN--AK------TMT------TSHLKQCIEL-EQQFD

FLKDL----------

>NF-YC_Q7ZVI1_BRARE#3#86 | 84 | Q7ZVI1; | PD003659 135 bp

-------SKKKKYNARFPPARIKKIMQTDE--------------EI--G---KVAAAVPV

IISRALELFLESLLTKACDVTQSRN--AK------TMT------TSHLKQCIEL-EQQFD

FLKDL----------

>NF-YC_Q9GSP1_DROME#3#86 | 84 | Q9GSP1; | PD003659 135 bp

-------SKKKKYNARFPAGRIKKIMQSDE--------------EI--G---KVAQAVPV

IISRTLELFVESLLTKTLRITNARN--AK------TLS------PSHMRQCIVS-EKRFD

FLKEL----------

>NF-YC_Q9W2E3_DROME#3#86 | 84 | Q9W2E3; | PD003659 135 bp

-------SKKKKYNARFPAGRIKKIMQSDE--------------EI--G---KVAQAVPV

IISRTLELFVESLLTKTLRITNARN--AK------TLS------PSHMRQCIVS-EKRFD

FLKEL----------

>NF-YC_Q8MLV5_DROME#1#66 | 66 | Q8MLV5; | PD003659 135 bp

-------------------------MQSDE--------------EI--G---KVAQAVPV

IISRTLELFVESLLTKTLRITNARN--AK------TLS------PSHMRQCIVS-EKRFD

FLKEL----------

>NF-YC_Q8W0W6_MAIZE#2#80 | 79 | Q8W0W6; | PD003659 135 bp

---------RKKLGTRFPAARIKKIMQADE--------------DV--G---KIALAVPV

LVSRSLELFLQDLIDRTYEITLQSG--AK------TLN------SFHLKQCVKR-YSSFD

FL-------------

>NF-YC_Q8W0W7_ORYSA#2#80 | 79 | Q8W0W7; | PD003659 135 bp

---------RKKLGTRFPAARIKKIMQADE--------------DV--G---KIALAVPV

LVSRALELFLQDLIDRTYEITLQSG--AK------TLN------SFHLKQCVRR-YSSFD

FL-------------

>NF-YC_Q9LHG0_ARATH#2#83 | 82 | Q9LHG0; | PD003659 135 bp

---------RKKLDTRFPAARIKKIMQADE--------------DV--G---KIALAVPV

LVSKSLELFLQDLCDRTYEITLERG--AK------TVS------SLHLKHCVER-YNVFD

FLREV----------

>NF-YC_Q9C7A7_ARATH#16#87 | 72 | Q9C7A7; | PD003659 135 bp

-------------------ARIKKIMQADE--------------DV--G---KIALAVPV

LVSKSLELFLQDLCDRTYEITLERG--AK------TVS------SLHLKHCVER-YNVFD

FLREV----------

>NF-YC_Q20237_CAEEL#68#137 | 70 | Q20237; | PD003659 135 bp

------------------PTRIKKVMQSDE--------------DI--G---RMVQSVPV

SIGRAMEHFAEKFLQAAAEATQFTS--SK------TLN------PQHMKQAVLN-TPHFS

FL-------------

>NF-YC_Q86G95_CAEEL#42#111 | 70 | Q86G95; | PD003659 135 bp

------------------PTRIKKVMQSDE--------------DI--G---RMVQSVPV

SIGRAMEHFAEKFLQAAAEATQFTS--SK------TLN------PQHMKQAVLN-TPHFS

FL-------------

>NF-YC_Q8SQT6_EEEEE#10#93 | 84 | Q8SQT6; | PD003659 135 bp

------------KLTRFPISRLKRIMQLNE--------------DI--G---KIGASVPV

VASKAIEMFLTEIVGLTLKEARKKS--SS------RMS------SEFIIRATES-DPKFA

FLKNMEQFKN-----

>NF-YC_Q7QXC2_EEEEE#23#95 | 73 | Q7QXC2; | PD003659 135 bp

---------------RLPISRVRAICRTVP--------------TI--T---LLSAEAPL

LISRLAELFIADVTNQSYQMAIRSN--AT------TVT------EDDVAHVFNV-TPEYD

FL-------------

Family: NOZZLE

>NOZZLE_gi|5007009|gb|AAD37775_1|AF146794_1 gi|5007009|gb|AAD37775.1|AF146794_1 A.thaliana 399 bp

MATSLFFMSTDQNSVGNPNDLLRNTRLVVNSSGEIRTE-TLKSRGRKPGSKTGQQKQKKP

TLRGMGVAKLERQRIEEEKKQL-AAATVGDTSSVASISNN-ATRLPVPVDPGVVLQGFPS

--------SLGS---------------------------NRIYCGG-----VGSGQVMID

PVISPWGFVETSSTTHELSSISNPQMFNASSNNRCDTCFKKKRLDGDQNNVVRSNG----

--GGFSKYTMI--PPPMNGYDQYLLQSDHHQRSQGFLYDHRIARAASVSASSTTINPYFN

EATNHTG-PMEEFGSYMEGNPRNG--SGGVKEYEFFPGKYGERVSV--------------

------------VATTSSLVGDCSPN---TIDLSLKL--

>NOZZLE_gi|86211341|gb|ABC87311_1| gi|86211341|gb|ABC87311.1| sporocyteless [Brassica juncea] 399 bp

MATSLFFMSTDQNSVRNPNELLRNTYLV---NGEIRTEPPKKSRGRKPGSKTAQQNQKEP

TLRGMGVAKLERFIAEEEKKKMVVVAGEGDTS--AASPNT-ATRFP---DRGVVLQGFPS

YGGGPTNTSLGGYTR------------------------SRFLCGG-----AGSGQIMID

PVCSPWGFVETSA--HELSSIPNPQMYNASSNH-CDTCFKKKRLDGDQ-NVVRSNG----

--GGFSKYTMMIPPPPMNGYDE-LLPPD--QRSQGFFYDQRIARSA--PAASASFNPYFN

EATNLTGYSREEFGSV---NPRNG--TRGVKEYEFFPGKYDDFHGK--------------

------------SFPVATSVGDCSPNTSSTIDLSLKL--

>NOZZLE_gi|147769696|emb|CAN74467_1| gi|147769696|emb|CAN74467.1| hypothetical protein VITISV_004771 [Vitis vinifera] 399 bp

M-----------------------------------PNRSSTSRARKAAS-----AQKKQ

PQRGMGVAQLERLRVEEW-KKMTGITHLQPMSLPGHFRYQIPYQFA---EPPPAPASGQV

QPG----LGSPGWVP-------------------------RFGGGGFHGFSGGGGFRVVD

PYRNDGAVGTLTEASRELSSMPKMD----CVFDRCDVCFKKKRMNADNLGXX--------

--XGREKYAET--PPINSXBFLGLDMANDENRD--------LSRRR--SPMTATYACY-N

HSE-----DAEVVAVHRKGNSVNG---RIFMEYEFFPGKGEASTSVVGGGGGGGEASLAI

PTAEAATTTSSRNAS-------------NVVDLSLKLSY

>NOZZLE_gi|87240380|gb|ABD32238_1| gi|87240380|gb|ABD32238.1| hypothetical protein MtrDRAFT_AC148815g15v2 [Medicago truncatula] 399 bp

MASA--------------------------------SENQAKNKGRKNGK-----GPKKP

-LRGVGVEQLEKQRIAALMKKHFDLPHQQQVLKYGAFSPTGPFQFP---QQQMMMNENTT

NNNNNTVLVGSGYVPFVGSNVGAGWIVPNNQNQNQNNIKNRVVVGG---GNYGSGSLL--

--CSSRNPFEPS---KELSSMPNLH----HS-QPFDLCLKKTRLNEEESNVMRGAGINTR

NLEAWSNHHYV--HDFLGGYMP--------------------------QPQPSAYPTHHN

LDE-----YVEVVAVHRRGSSSSGGKDKVFMEYDFFHEKDGRDTSF---------KNLKF

PTIGEASSSSSLSLTVSAYGGDNATTDLSSIDLSLKL--

Family: SAP

>SAP_gi|9758652|dbj|BAB09276_1| gi|9758652|dbj|BAB09276.1| sterile apetala [Arabidopsis thaliana] 446 bp

MSTSSSSSDNGAGGSGGVFEAPSPSRPRRGANDVWPEPFLESLAVQVAVNASTSAGLLAA

APALANVFRVCTTWHAVSRSDHLWQLLSRQVWARTHLMHDTWRDEFIYRHRTARNFRTRT

HTYFTLQFDPSDVDEPDSLSCRCLTLSDLYLAAGFADGTVRLFLLNNRLHVRTLRPPLRD

RFGRFSRAVSGIVISDSRLTFATMDGDIHVAEIDGVGHTRTAYAGDIVNDGALVDFTGCG

RWWVGLFAGVPGRAFHIWDCNSEETTFVGGTLTDPEAVMGWHTLTELTTSLGRLRISGNE

TAVACTRWRIMVIDLRNQGVIIGEDEEQRRGLIVTGFDANDEAYVRLDSRGNASVRRVNT

QQTVCEFRVSGAAQRRVMGCVNRLHALMCAGGIMRVWEVERGEYLYSIRERVGEVDAIVA

DDRHVAVASASSTAQSIIHLWDFGAL

>SAP_gi|4033856|emb|CAA11128_1| gi|4033856|emb|CAA11128.1| sterile apetala [Arabidopsis thaliana] 446 bp

MSTSSSSSDNGAGGSGGVFEAPSPSRPRRGANDVWPEPFLESLAVQVAVNASTSAGLLAA

APALANVFRVCTTWLAVSRSDHLWQLLSRQVWARTHLMHDTWRDEFIYRHRTARNFRTRT

HTYFTLQFDPSDVDEPDSLSCRCLTLSDLYLAGGFADGTVRLFLLNNRLHVRTLRPPLRD

RFGRFSRAVSGIVISDSRLTFATMDGDIHVAEIDGVGHTRTAYAGDIVNDGALVDFTGCG

RWWVGLFAGVPGRAFHIWDCNSEETTFVGGTLTDPEAVMGWHTLTELTTSLGRLRISGNE

TAVACTRWRIMVIDLRNQGVIIGEDEEPRRGLIVTGFDANDEAYVRLDSRGNASVRRVNT

QQTVCEFRVSGAAQRRVMGCVNRLHALMCAGGIMRVWEVERGEYLYSIRERVGEVDAIVA

DDRHVAVASASSTAQSIIHLWDFGAL

Family: SW13

>SW13_At1g21700 1178 bp

MPASEDRRGKWKRKKRGGLSAARKPKQEEEDMEEEDEENNNNNNEEMDDVENADELQQNG

GATPDPGLGIGEVVEDSGSRISDFPAVVKRVVIRPHASVMAVVAAERAGLIGETRGQGSL

PALENISFGQLQALSTVPADSLDLERSDGSSSAYVISPPPIMDGEGVVKRFGDLVHVLPM

HSDWFAPNTVDRLERQVVPQFFSGKSPNHTPESYMEFRNAIVSKYVENPEKTLTISDCQG

LVDGVDIEDFARVFRFLDHWGIINYCATAQSHPGPLR-DVSDVREDTNGEVNVPSAALTS

IDSLIKF----DKPNCRHKGGEVYSSLPS---LDGDSPDL-DIRIREHLCDSHCNHCSRP

LP-TVYFQSQKKGDILLCCDCFHHG-RFVVGHSCLDFVRVDPMKFYGDQDGDNWTDQETL

LLLEAVELYN-ENWVQIADHVGSKSKAQCILHFLRLPVEDGLLDN----VEV--------

-------------------------SGVTNTEN----PTNGYDHKGTD---SNGDLPGYS

EQ-------------------------------------------GSDTEIKLPFVKSPN

PVMALVAFLA---SAVGPRVAASCAHESLS-VLSE-------------------------

----------------------------------DDRMKSEGMQGKEAS-----------

--------------------------------------------------------LLDG

---ENQQQDGAHKTSSQNGAEAQTPLPQ--------------------------------

------------------------------------------------------------

--------------------------------DKVMAAFRAGLSAAATKAKLFADHEERE

IQR--------------LSANIVNHQL-KRMELKLKQFAEIETLLMKECEQVEKTRQRFS

AERARMLSARFGSPGGISPQTNNLQGMSLSTGGNNINSLMHQQHQQQQASATSQPSIIPG

FSNNPQVQAQMHFMARQQQQQQQQQQQQQQAFSFGPRLPLNAIQTNAGSTASPNVMFGNN

QLNNPAAAGAASINQPSFSHPMVRSSTGSGSGSGLGLN

>SW13_At2g33610 1178 bp

M-----------------------------------------------------------

------------------------------------------------------------

--AMKAPDPGGSGEILPSTPSLSETTSGGAAAASKSAQLPSSSSD------IDNIHV-PS

YSSWFSWTDINDCEVRSLPEFFDSRSSSKNPKFYLYLRNSIIKQYRDDHPRKISFTDVRR

TLVS-DVVSIRRVFDFLDSWGLINYNSSA-------------------------------

-----------SAKPLKWEEKEAGKSAGD---AASEPATT-----VKETAKRNCNGCKAI

CS-IACFAC-DKYDLTLCARCYVRS-NYRVGINSSEFKRVE----ISEESKPEWSDKEIL

LLLEAVMHYG-DDWKKVASHVIGRTEKDCVSQFVKLPFGEQFVKESDSEDGL--------

-------------------------EMFDQIKDSDIPESEGIDKDGSS------------

---------------------------------------------PNKRIKLTPLADASN

PIMAQAAFLS---ALAGTNVAEAAARAAVR-ALSD-------------------------

------------------------------------------------------------

---------------------------------------------------------VDY

EADKNASRDPNRQDANAASSGETTRNES--------------------------------

------------------------------------------------------------

--------------------------------ER-----------AWADAKSLIEKEEHE

VEG--------------AIKETVEVEVSNNLQLSIDFLLYGYTQ----------------

------------------------------------------------------------

------------------------------------------------------------

--------------------------------------

>SW13_At2g47620 1178 bp

M-----------------------------------------------------------

------------------------------------------------------------

-------------------------------------EATDPSAE------IELYTI-PA

QSSWFLWDDIHEIERREFAEFFTESSITRTPKVYKEYRDFIINKFREDTCRRLTFTSVRK

FLVG-DVNLLQKVFLFLEKWGLINFSSSLKKNDHLLSVDNAKIEQGTPAGIRVTATP---

-NSLRPI----TAPPLVEERVETGIKVPP---LTSYSDVFSDLKKPDHVL--VCAHCGER

CD-SPFYQ-HNKGIVNICEKCFKNG-NYGENNTADDFKL------IGNSAAAVWTEEEIL

LLLESVLKHG-DDWELISQSVSTKSRLDCISKLIELPFGEFLMGSASGRLNP--------

-------------------------SILTEDENTEQVQTDGQEHEETETREEKEDRVNED

EP-------------------------------------------PAKRKRVALISEGDS

SLMKQVAAMA---SKVGPSVATAAAKAALA-ALCD-------------------------

----------------------------------EASCPKEIFDTDDYSNF---------

--------------------------------------------------------TVDR

---ANGEKDTDMEEQQEEKDGPQGLPVA--------------------------------

------------------------------------------------------------

--------------------------------LRIRASVATALGAAAAQAKILADQEERE

MEQ--------------LAATVIEQQL-KKLQSKLKFLDDLESIMDEEEKVIEGVKETII

QERVSVLQCAFRSG----------------------------------------------

------------------------------------------------------------

-------------ITKRWDHTYVK--------------

>SW13_At4g34430 1178 bp

M-----------------------------------------------------------

------------------------------------------------------------

------------------------------------------------------------

------------------------------------------GKFHSNPNIQIELKDLTE

LEVG-DSEAKQEVMEFLDYWGLINFHPFPPTDTGSTASDHDDLGDKE-----------SL

LNSLYRFQVDEACPPLVHKPRFTAQATPSGLFPDPMAADE-LLKQEGPAVEYHCNSCSAD

CS-RKRYHCPKQADFDLCTECFNSG-KFSSDMSSSDFILMEPAEAPGVGSG-KWTDQETL

LLLEALEIFK-ENWNEIAEHVATKTKAQCMLHFLQMPIEDAFLDQIDYKDPISKDTTDLA

VSKDDNSVLKDAPEEAENKKRVDEDETMKEVPE----PEDGNEEKVSQ---ESSKPGDAS

EETNEMEAEQKTPKLETAIEERCKDEADENIALKALTEAFEDVGHSSTPEASFSFADLGN

PVMGLAAFLV---RLAGSDVATASARASIK-SLHSNSGMLLATRHCYILEDPPDNKKDPT

KSKSADAEGNDDNSHKDDQPEEKSKKAEEVSLNSDDREMPDTDTGKETQDSVSEEKQPGS

RTENSTTKLDAVQEKRSSKPVTTDNS--------EKPVDIICPSQDKCSGKELQEPLKDG

NKLSSENKDASQSTVSQSAADASQPEASRDVEMKDTLQSEKDPEDVVKTVGEKVQLAKEE

GANDVLSTPDKSVS------QQPIGSASAPENGTAGSLFLSLSDTLITLPTLHDSFISYR

FVIEMLFAGGNPNIEGKKEKDICEGTKDKYNIEKLKRAAISAISAAAVKAKNLAKQEEDQ

IRQ--------------LSGSLIEKQQLHKLEAKLSIFNEAESLTMRVREQLERSRQRLY

HERAQIIAARLGVPPSMSSKA----------------SLPTNRIAANFANVAQRPPMGMA

FPRPPM------------------------------------------------------

----PRPPGFPVPGSFVAATTMTGSSDPSPGSDNVSSV

>SW13_At3g07740 1178 bp

M-----------------------------------------------------------

------------------------------------------------------------

------------------------------------------------------------

------------------------------------GRSKLASRPAEE------------

------------------------------------------------------------

-----------DLNPGKSKRKKISLGPEN---AAASISTGIEAGNERKPGLYCCNYCDKD

LSGLVRFKCAVCMDFDLCVECFSVGVELNRHKNSHPYRVMDNLSFSLVTS--DWNADEEI

LLLEAIATYGFGNWKEVADHVGSKTTTECIKHFNSAYMQSPCFPLPDLSHTIGKSKDELL

AMSKDSAVKTEIP------------AFVRLSPKEELPVSAEIKHEASGKVNEIDPPLSA-

---------------------------------------------LAGVKKKGNVPQAKD

IIKLEAAKQQSDRSVGEKKLRLPGEKVPLVTELYG----------------------YNL

KREEFEIEHDNDAEQLL-----------------ADMEFKDSDTDAEREQKLQVLRIYSK

RLDERKRRKEFVLERNLLYPDQYEMSLSAEERKIYKSCKVFARFQSKEEHKELIKKVIEE

HQILRRIEDLQE---ARTAGCRTTSDANRFIEEKRKKEAE-----------ESMLLRLNH

GAPGSIAGKTLKSPRGLPRNLHPFGSDSLPKVTP--------------------------

--------------------------------PRIYSGLDTWDVDGLLGADLLSETEKKM

CNETRILPVHYLKMLDILTREIKKGQIKKK--------SDAYSFFKVEPSKVDRVYDMLV

HKGIG-------------------------------------------------------

------------------------------------------------------------

-----------------------------------DST

>SW13_At4g16420 1178 bp

M-----------------------------------------------------------

------------------------------------------------------------

------------------------------------------------------------

------------------------------------GRSRGNFQNFED------------

------------------------------------------------------------

-------------PTQRTRKKKNAANVEN--FESTSLVPGAEGG-----GKYNCDYCQKD

ITGKIRIKCAVCPDFDLCIECMSVGAEITPHKCDHPYRVMGNLTFPLICP--DWSADDEM

LLLEGLEIYGLGNWAEVAEHVGTKSKEQCLEHYRNIYLNSPFFPLPDMSHVAGKNRKELQ

AMAKGRIDDKK----------------AEQNMKEEYPFS---------------PPKVK-

---------------------------------------------VEDTQKESFV-----

-----------DRSFGGKKPVSTSVNNSLV-ELSN----------------------YNQ

KREEFDPEYDNDAEQLL-----------------AEMEFKENDTPEEHELKLRVLRIYSK

RLDERKRRKEFIIERNLLYPNPFEKDLSQEEKVQCRRLDVFMRFHSKEEHDELLRNVVSE

YRMVKRLKDLKE---AQVAGCRSTAEAERYLGRKRKRENE-----------EGMNRGKES

GQFGQIAGEMGSRP--------PVQASS--------------------------------

------------------------------------SYVNDLDLIGFTESQLLSESEKRL

CSEVKLVPPVYLQMQQVMSHEIFKGNVTKK--------SDAYSLFKIDPTKVDRVYDMLV

KKGIAQL-----------------------------------------------------

------------------------------------------------------------

--------------------------------------

Family: Topless

>Topless_ACN26165 unknown [Zea mays] 1166 bp

MTSLSRELVFLILQFLDEEKFKETVHKLEQESGFYFNIKYFEDEVLNGNWDEVERYLGGF

TKVDDNRYSMKIFFEIRKQKYLEALDKHDRSKAVEILVKDLKVFASFNEELFKEITQLLT

LENFRENEQLSKYGDTKSARQIMLAELKKLIEANPLFRDKLQFPSLKTSRLRTLINQSLN

WQHQLCKNPRPNPDIKTLFVDHSCGQPNGARAPSPANSPLLGSMPKPGGFPPLGAHGPFQ

PAPTPVP-PLAGWMSNPPAITHPAVSGGAIGFGTPTNPAVLL----KHPRTPTT------

--------GNPGMDYPSGDSDHISKRTRPVGMSEEMNLPVNMLPVTYP-QSHNYQQ----

-DDFHKAVARTLSQGSAPMSMDFHPLQQTLLLVGTNVGDIGLWDVGTKERLVVRNFKVWD

LGKCTMTLQAALVKDPAVSVNRIIWSPDGTLFGVAYSRHIVQIYSYNGGDDIRQHLEIDA

HVGGVNDIAFAHPNKQLCIITCGDDKTIKVWEATSGAKQFTFEGHEAPVYSVCPHYKENI

QFIFSTALDGKIKAWLYDNLGSRVDYDAPGHWCTTMAYSADGSRLFSCGTSKDGESHLVE

WNESEGAVKRTYQGFRKRSMGVVQFDTTRNRFLAAGDEFMIKIWDMDSTGLLTTIDADG-

--GLTASPRIRFNKEGTLLAVSTADNGIKILANADGLRLLRTLENRSFDASRNASEAVTK

PLINPLTAAANAAAA-----SSSGAGAPSAITAMNGDSRSLVDVKPRIADESLDKSKVWK

LMEITESSQCRSIKLADNMRTSKISRLIYTNSGLAILALTSSAVHLLWKWPRSDRN-SGK

ATASVSPTLWQPPS-GILMTNDTTDNNPEEAVHCFALSKNDSYVMSASGGKISLFNMMTF

KTMTTFMPAPPAATFLAFHPQDNNIIAIGMDDSTIQIYNVRIDEVKSKLRGHSKRITGLA

FSNVLNVLVSSGADAQLCVWNTDGWEKQKNRFLQIPSGRQSN-IL-DTRVQFHQDQMHFL

VVHETQIAIYETTKLEPVKQWPVRENSPPITHATFSCDSQLIYASFMDATVGIFNGSSLR

LQCRILPASYLPPNIS-PSVHPVVVAAHPSEASQFALGLTDGGVFVLEPLESERKWGNPP

PVENGSASNLSTPPPNGASSSDQPER

>Topless_NP_001049587 Os03g0254700 [Oryza sativa Japonica Group] 1166 bp

MSSLSRELVFLILQFLDEEKFKETVHKLEQESGFYFNMKYFEDEVINGNWDEVERYLGGF

TKVDDNRYSMKIFFEIRKQKYLEALDKHDRSKAVEILVKDLKVFASFNEELFKEITQLLT

LENFRENEQLSKYGDTKSARAIMLVELKKLIEANPLFRDKLQFPNLKSSRLRTLINQSLN

WQHQLCKNPRPNPDIKTLFVDHSCGQPNGARAPSPANNPLLGSIPKPGGFPPLGAHAPFQ

PAPTPVP-PLAGWMSNPPAVTHPAVSGGAIGFGTPTNPAAIL----KHPRTPTT------

--------ANPSMDYPSGDSDHVSKRTRPVGMSEEVNLPVNMLPVTYP-QSHSYPQ----

-DDFHKNVARTLSQGSTPMSMDFHPVQQTLLLVGTNVGDIGLWDVGTKERLVLRNFKVWD

LTKCSMALQASLVKDPTVSVNRIIWSPDGTLFGVAYSRHIVQIYSYHGGDDIRQHLEIDA

HVGGVNDIAFAHPNKQLCIITCGDDKTIKVWEATSGAKQFTFEGHEAPVYSVCPHYKENI

QFIFSTALDGKIKAWLYDNLGSRVDYDAPGHWCTTMAYSADGSRLFSCGTSKDGESHLVE

WNESEGAVKRTYQGFRKRSMGVVQFDTTRNRFLAAGDEFLIKIWDMDNTSLLTTIDADG-

--GLPASPRVRFNKEGTLLAVSTHENGIKILANADGVRLLRTLENRSFDASRSASETVTK

PLMNPLTAAAAAAASAAAAGTSSGNAAPPAITALNGDSRSLVDVKPRIADEPLDKSKVWK

LMEITESSQCRSLKLTDNMRTSKISRLIYTNSGVAILALASNAVHLLWKWPRNDRNSSGK

ATASVSPQLWQPPS-GILMTNDITD-NPEEAVHCFALSKNDSYVMSASGGKISLFNMMTF

KTMTTFMPPPPAATFLAFHPQDNNIIAIGMDDSTIQIYNVRIDEVKSKLRGHSKKITGLA

FSNVLNVLVSSGADAQICVWSTDGWDKLKSRMLQIPSSRPSSIIL-DTRVQFHQDQLHFL

VVHETQIAIYETTKLEPVKQWPVRENSSPITHAMFSCDSQLIYASFLDATVCIFNASSLR

LQCRILPASYLPQNIS-SNVYPVVVAAHPSEANQFALGLTDGGVYVLEPLESERKWGNPP

PAENGSTSALST-PPNGASSSDQPER

>Topless_NP_563981 TPL (TOPLESS); protein binding / protein homodimerization/ transcription repressor [Arabidopsis thaliana] 1166 bp

MSSLSRELVFLILQFLDEEKFKETVHKLEQESGFFFNMKYFEDEVHNGNWDEVEKYLSGF

TKVDDNRYSMKIFFEIRKQKYLEALDKHDRPKAVDILVKDLKVFSTFNEELFKEITQLLT

LENFRENEQLSKYGDTKSARAIMLVELKKLIEANPLFRDKLQFPTLRNSRLRTLINQSLN

WQHQLCKNPRPNPDIKTLFVDHSCGPPNGARAPSPVNNPLLGGIPKAGGFPPLGAHGPFQ

PTASPVPTPLAGWMSSPSSVPHPAVSAGAIALGGPSIPAA-L----KHPRTPPT------

---------NASLDYPSADSEHVSKRTRPMGISDEVNLGVNMLPMSFSGQAHGHSPAFKA

PDDLPKTVARTLSQGSSPMSMDFHPIKQTLLLVGTNVGDIGLWEVGSRERLVQKTFKVWD

LSKCSMPLQAALVKEPVVSVNRVIWSPDGSLFGVAYSRHIVQLYSYHGGEDMRQHLEIDA

HVGGVNDISFSTPNKQLCVITCGDDKTIKVWDAATGVKRHTFEGHEAPVYSVCPHYKENI

QFIFSTALDGKIKAWLYDNMGSRVDYDAPGRWCTTMAYSADGTRLFSCGTSKDGESFIVE

WNESEGAVKRTYQGFHKRSLGVVQFDTTKNRYLAAGDDFSIKFWDMDAVQLLTAIDGDG-

--GLQASPRIRFNKEGSLLAVSGNENVIKIMANSDGLRLLHTFENISSESS--------K

PAINSIAAAAAAAAT--SAGHADRSANVVSIQGMNGDSRNMVDVKPVITEESNDKSKIWK

LTEVSEPSQCRSLRLPENLRVAKISRLIFTNSGNAILALASNAIHLLWKWQRNERNATGK

ATASLPPQQWQPAS-GILMTNDVAETNPEEAVPCFALSKNDSYVMSASGGKISLFNMMTF

KTMATFMPPPPAATFLAFHPQDNNIIAIGMDDSTIQIYNVRVDEVKSKLKGHSKRITGLA

FSNVLNVLVSSGADAQLCVWNTDGWEKQRSKVLPLPQGRPNSAPS-DTRVQFHQDQAHFL

VVHETQLAIYETTKLECMKQWAVRESLAPITHATFSCDSQLVYASFMDATVCVFSSANLR

LRCRVNPSAYLPASLSNSNVHPLVIAAHPQEPNMFAVGLSDGGVHIFEPLESEGKWGVAP

PAENGSASGAPTAPSVGASASDQPQR

>Topless_NP_849913 TPR1 (TOPLESS-RELATED 1) [Arabidopsis thaliana] 1166 bp

MSSLSRELVFLILQFLDEEKFKETVHKLEQESGFFFNMKYFEDEVHNGNWDEVEKYLSGF

TKVDDNRYSMKIFFEIRKQKYLEALDRHDRPKAVDILVKDLKVFSTFNEELFKEITQLLT

LENFRENEQLSKYGDTKSARAIMLVELKKLIEANPLFRDKLQFPTLRTSRLRTLINQSLN

WQHQLCKNPRPNPDIKTLFVDHSCRLPNDARAPSPVNNPLLGSLPKAEGFPPLGAHGPFQ

PTPSPVPTPLAGWMSSPSSVPHPAVSGGPIALGAPSIQAA-L----KHPRTPPS------

---------NSAVDYPSGDSDHVSKRTRPMGISDEVSLGVNMLPMTFPGQAHGHNQTFKA

PDDLPKTVARTLSQGSSPMSMDFHPIKQTLLLVGTNVGDIGLWEVGSRERLVQKTFKVWD

LSKCSMPLQAALVKEPVVSVNRVIWSPDGSLFGVAYSRHIVQLYSYHGGEDMRQHLEIDA

HVGGVNDIAFSTPNKQLCVTTCGDDKTIKVWDAATGVKRYTFEGHEAPVYSICPHYKENI

QFIFSTALDGKIKAWLYDNMGSRVDYEAPGRWCTTMAYSADGTRLFSCGTSKDGESFIVE

WNESEGAVKRTYQGFHKRSLGVVQFDTTKNRYLAAGDDFSIKFWDMDTIQLLTAIDADG-

--GLQASPRIRFNKEGSLLAVSANDNMIKVMANSDGLRLLHTVENLSSESS--------K

PAINSIPM-------------VERPASVVSIPGMNGDSRNMVDVKPVITEESNDKSKVWK

LTEVGEPSQCRSLRLPENMRVTKISRLIFTNSGNAILALASNAIHLLWKWQRNDRNATGK

ATASLPPQQWQPAS-GILMTNDVAETNPEEAVPCFALSKNDSYVMSASGGKISLFNMMTF

KTMATFMPPPPAATFLAFHPQDNNIIAIGMDDSTIQIYNVRVDEVKSKLKGHSKRITGLA

FSNVLNVLVSSGADAQLCVWNTDGWEKQKSKVLQIPQGRSTSSLS-DTRVQFHQDQVHFL

VVHETQLAIYETTKLECMKQWPVRESAAPITHATFSCDSQLIYTSFMDATICVFSSANLR

LRCRVNPSAYLPASLSNSNVHPLVIAAHPQESNMFAVGLSDGGVHIFEPLESEGKWGVAP

PPENGSASAVTATPSVGASASDQPQR

>Topless_XP_002268265 PREDICTED: hypothetical protein [Vitis vinifera] 1166 bp

MSSLSRELVFLILQFLDEEKFKETVHKLEQESGFFFNMKYFEDEVHSGNWDEVEKYLSGF

TKVDDNRYSMKIFFEIRKQKYLEALDKHDRSKAVEILVKDLKVFATFNEELFKEITQLLT

LENFRENEQLSKYGDTKSARAIMLVELKKLIEANPLFRDKLQFPHLKNSRLRTLINQSLN

WQHQLCKNPRPNPDIKTLFVDHTCGQPNGARAPSPANNPLLGSLPKAGGFPPLGAHGPFQ

PTPAPVPTPL--WMSNPSTVTHPAVSGGPIGLGAPSIPAA-L----KHPRTPPT------

---------NPSVDYPSGDSEHVAKRGRPMGISDEVNLPVNVLPVTFPG--HGHSQAFNA

PDDLPKTLVRNLTQGSSPMSMDFHPVQQTLLLVGTNVGDIGLWEVGSKQKLVSRNFKVWD

IGACSVPLQAALAKDPGVSVNRIIWSPDGSLFGVAYSRHIVQIYSYHGGDDVRQHLEIDA

HAGGVNDLAFSHPNKQLCVITCGDDKTIKVWDATNGTKQYTFEGHEDAVYSVCPHYKENI

QFIFSTALDGKIKAWLYDNLGSRVDYDAPGRWCTTMAYSADGTRLFSCGTSKDGDSYIVE

WNESEGAVKRTYQGFRKRSLGVVQFDTTKNRFLAAGDDFSIKFWDMDNIQLLTILDAEGG

LPGLPASPRIRFNKDGTLLAVSANENSIKILANSDGLRLLRTFDNLSYDASR-ASESVTK

PAINSISAAAAAAAA-TSAGLADRGASVVAIAGMNGDARNMGDVKPRLAEETNDKSKIWK

LTEINETSQCRSLRLQENLRITKISRLIYTNSGNAILALASNAIHFLWKWQRNDRNSSGK

ATATVSPQLWQPTS-GILMTNDVADTNPEEAVPCFALSKNDSYVMSASGGKISLFNMMTF

KTMTTFMPPPPAATFLAFHPQDNNIIAIGMDDSTIQIYNVRVDEVKSKLKGHSKRITGLA

FSHVLNVLVSSGADAQLCVWSSDGWEKQKSRFLQVPAGRTSTGQS-DTRVQFHQDQTHFL

VVHETQLAIYEATKLDCVKQWVQREAAAPISHATFSCDSLLVYASFLDATVCVFSAANLR

LRCRINPTAYLPASVSNSNVHPLVIAAHPQEPNQFALGLSDGGVCVFEPLESEGKWGVPP

PVENGSASSVPATPSVGPSGSDQPQR

>Topless_XP_002324314 predicted protein [Populus trichocarpa] 1166 bp

MAALCKDLVLLISQFLDEEGFKETARMLERESGYYFSMKFFEDMIRSGNWDEAERYLSCF

TKLDDNRYSTKIYFEIRKQKFLEVLDNDERSKALDILMKDLKAFAPDNEELLKEMTLLLT

LNNIRDHESLSMYSDAESARKVMMVELKKVIEANPLLRDKLEFPNIANHRLRRLINQSLN

WQHMHCAYPQPNPDIRTLFVDHIC-------VPIPSDDHLFSAASDSNPLPS-------Q

TTSMLVSTSSA-SNSTSSSEAHSSISSEALSLGVPTNIGSFIVVIEKKLLLSLTIYSMFV

AAMIEVLEDNTTVNDSGIPKNRIVNLKRP---SNEASLIFHFFLLKHC--------SVNI

SDDLPKNVFRILNEGSSPTSMDFHPEKQTVLLVGTTVGDIGLWEVSSGESLLSRNFKVWD

IAACSMMFKATLLKDPSVSVNRVAWSPEGGLFGVAYSKHLVQIYSYNEAKDARQQLEIDA

HVGGVNDLTFSAPEKQLLVITCGDDKIVKAWDATDGVKMYTFEGHDAPVYSLCPYSKGNV

HFVFATSVNGNIKVWLYDNLGARVDYDAPGLGCTSMAYSGD-RRLFSCGTSGSGESFLVE

WDDSEGAIKRTYLGLQKNSSSVVQFDIMKNQVLAAGDEHVIKIWDMNKIELFTTIDAEG-

--GLPANPCVRFNKEGTLLAVSANDNKIKILAKDGSLQSLHTTENCLDDDFRLVSEAISK

----------GACAQ-----DADEAVAKQCFNLL--QNGNLKAVKSKITGK-DTKSKSGR

LIELNSPSQCQILRLPSHMKANKISRLIYNNAGNSILALTSNATHLYWKWPQNDFDLSDT

AAAKVSPQLWQPRSYSGLMTNDLTGSNPEETVPCFALSRNDSYLMSSSGGRISLYNLLKF

KTMLSIMQPPPAATCIAFHPQDNNILAIGRDNSTILIYNVRSAKVITILEGHSKRVSGLA

FSNDLNLLVSSGADAQIFVWNVEGWYKQRSTFLQIPDGRIPFSLSTDTHIQFHQNQTEFL

SVHETHLAIYEARKLECVKQWIPGDFATPISHATFSCDGQMVYASFLDGLVSIFDASDFQ

LYCQINPTAYLFPT-SSLGVYPIAIAAHPQEPNQFAVGLTDGGVIVFEPPISAGKWSMLT

ADENGSASKLPA-ESEG-------NY

Family: Trihelix

>Trihelix_AtGT-2N-AAM64675 81 bp

----WPRPETLALLRIRSEMDKAFRDSTLKAPLWEEISRKMMELGYK-------------

RSSKKCKEKFENVYKYHKRTK

>Trihelix_AtGTL1C-AAM98226 81 bp

----WPREETLALLRIRSDMDSTFRDATLKAPLWEHVSRKLLELGYK-------------

RSSKKCKEKFENVQKYYKRTK

>Trihelix_OsGT-2N-BAD25570 81 bp

----WPREETLALIRIRSEMDAAFRNATLKAPVWEELSRRLAELGYQ-------------

RSGKKCKEKFENVDKYYKRTK

>Trihelix_AtGT-2C-AAM64675 81 bp

----WPKTEVEALIRIRKNLEANYQENGTKGPLWEEISAGMRRLGYN-------------

RSAKRCKEKWENINKYFKKVK

>Trihelix_OsGT-2C-BAD25570 81 bp

----WPKEEVQALIDLRMEKEEQYNDMGPKGPLWEEIAAGMQRIGYN-------------

RSAKRCKEKWENINKYFKKVK

>Trihelix_AtGTL1N-AAM98226 81 bp

----WPKAEILALINLRSGMEPRYQDNVPKGLLWEEISTSMKRMGYN-------------

RNAKRCKEKWENINKYYKKVK

>Trihelix_N37430-NP_568506 81 bp

----WCSDEVLALLRFRSTVENWFPEFT-----WEHTSRKLAEVGFK-------------

RSPQECKEKFEEERRYFNSNN

>Trihelix_AB023041-NP_189228 81 bp

----WAQDETRTLISLRREMDNLFNTSKSNKHLWEQISKKMREKGFD-------------

RSPSMCTDKWRNILKEFKKAK

>Trihelix_NtGT-1a-_PQ0446 81 bp

----WVQEETRALISLRRELDSLFNTSKSNKHLWDQISLKMREKGFD-------------

RSPTMCTDKWRNLLKEFKKAK

>Trihelix_AtGT-1-AAA66473 81 bp

----WVQDETRSLIMFRRGMDGLFNTSKSNKHLWEQISSKMREKGFD-------------

RSPTMCTDKWRNLLKEFKKAK

>Trihelix_ac003028 81 bp

----WSVEETKELIGIRGELDQTFMETKRNKLLWEVISNKMRDKSFP-------------

RSPEQCKCKWKNLVTRFKGCE

>Trihelix_AB007649-AAO42228 81 bp

----WKPEEIKKVIRMRGELHSRFQVVKGRMALWEEISSNLSAEGIN-------------

RSPGQCKSLWASLIQKYEESK

>Trihelix_11B9_6/1-447 81 bp

REDWWSEDATATLIEAWGDRYVNLNRGNLRQNDWKEVADAVNSSHGN-GRP---------

KTDVQCKNRIDTLKKKYKTEK

>Trihelix_F9F8_9/1-447 81 bp

REDWWSEDATATLIEAWGDRYVNLNRGNLRQNDWKEVADAVNSSHGN-GRP---------

KTDVQCKNRIDTLKKKYKTEK

>Trihelix_MOP10_9/1-447 81 bp

REDWWSEEATATLVEAWGNRYVKLNHGNLRQNDWKDVADAVNSRHGDNSRK---------

KTDLQCKNRVDTLKKKYKTEK

>Trihelix_F14P22_220/1-447 81 bp

REDCWSEEATFTLIQAWGNRYVDLSRGNLRQKHWQEVANAVNDRHYNTGRNVSAAKSQPY

RTDVQCKNRIDTLKKKYKVEK

>Trihelix_NtSIP1/1-447 81 bp

REDCWSEAATHTLVEAWGSHYLELKRGNLRQKYWQEVANAVNALHGHTKKQ--------Y

RTDIQCKNRIDTLKKKYKIEK

Family: ULT

>ULT_gi|18417145|ref|NP_567799_1| gi|18417145|ref|NP_567799.1| ULT1 (ULTRAPETALA1); DNA binding [Arabidopsis thaliana] 239 bp

MANNEGEMQCGSM-LFKQEELQEMSGVNVGGDYVEVMCGCTSHRYGDAVARLRVFPTGDL

EITCECTPGCDEDKLTPAAFEKHSGRETARKWKNNVWVIIGGEKVPLSKTVLLKYYNESS

KKCSRSNRSQGAKVCHRDEFVGCNDCGKERRFRLRSRDECRLHHNAMGDPNWKCSDFPYD

KITCEEEEERGSRKVYRGCTRSPSCKGCTSCVCFGCELCRFSECTCQTCVDFTSNVKA-

>ULT_gi|15226407|ref|NP_179677_1| gi|15226407|ref|NP_179677.1| ULT2 (ULTRAPETALA 2); DNA binding [Arabidopsis thaliana] 239 bp

M-----ERECGSKELFSKEELQEISGVHVGDDYVEVMCGCTSHRYGDAVARLKIFSDGEL

QITCQCTPACLEDKLTPAAFEKHSERETSRNWRNNVWVFIEGDKVPLSKTVLLRYYNKAL

KN------SNVSKVIHRDEFVGCSTCGKERRFRLRSRGECRMHHDAIAEPNWKCCDYPYD

KITCEEEEERGSRKVFRGCTRSPSCKGCTSCVCFGCKLCRFSDCNCQTCLDFTTNAKPI

Family: VIP3

>VIP3_ABK21104 unknown [Picea sitchensis] 662 bp

MQQ---------------------------------------------------------

------------------------------------------------------------

------------------------------------------------------------

------------------------------------------------------------

------------------------------------------------------------

------------------------------------------------------------

------------------------------------------------------------

-------------------GGAVDTEVTWEDQQNINKFGRLNNRFHELEDEIKGRK----

------------------------------------------------------------

------------------------------------EMTENLEDASNELILADEEIVRFQ

LGEVFSHMPKEEVENRLESMKEETTKELEKLEEEKESILAQMADLKKILYGKFNDSINLE

ED

>VIP3_ACU13902 unknown [Glycine max] 662 bp

MQQ---------------------------------------------------------

------------------------------------------------------------

------------------------------------------------------------

------------------------------------------------------------

------------------------------------------------------------

------------------------------------------------------------

------------------------------------------------------------

-------------------GGGSETEVTWEDQQNINKFGRLNNRFHELEDEIKIAK----

------------------------------------------------------------

------------------------------------ETNDNLEDASNELILTDEEVIRFQ

IGEVFAHVPKDEVENRIEQIKEVTSQKLEKLEEEKESVLAQMAELKKILYAKFNDSINLE

ED

>VIP3_ACF80576 unknown [Zea mays] 662 bp

------------------------------------------------------------

------------------------------------------------------------

------------------------------------------------------------

------------------------------------------------------------

------------------------------------------------------------

------------------------------------------------------------

------------------------------------------------------------

-----------------------------------------------------MFQ----

------------------------------------------------------------

------------------------------------ETNENLDDAGNELILSDEDVVRFQ

IGEVFAHMPRDDVETRLEQMKEDAAKKLERLEEEKESVLAQMAELKKILYGKFKDAINLE

ED

>VIP3_CAC35069 VIP3 protein [Zea mays] 662 bp

MQQ---------------------------------------------------------

------------------------------------------------------------

------------------------------------------------------------

------------------------------------------------------------

------------------------------------------------------------

------------------------------------------------------------

------------------------------------------------------------

-------------------GDGTEAQVTWEDQQNINRFGRLNNRLHELQEEIKLAK----

------------------------------------------------------------

------------------------------------ETNENLDDAGNELILSDEDVVRFQ

IGEVFAHMPRDDVETRLEQMKEDAAKKLERLEEEKESILAQMAELKKILYGKFKDAINLE

ED

>VIP3_NP_001105143 VIP3 protein [Zea mays] 662 bp

MQQ---------------------------------------------------------

------------------------------------------------------------

------------------------------------------------------------

------------------------------------------------------------

------------------------------------------------------------

------------------------------------------------------------

------------------------------------------------------------

-------------------GDGTEAQVTWEDQQNINRFGRLNNRLHELQDEIKLAK----

------------------------------------------------------------

------------------------------------ETNENLDDAGNELILSDEDVVRFQ

IGEVFAHMPRDDVETRLEQMKEDAAKKLERLEEEKESILAQMAELKKILYGKFKDAINLE

ED

>VIP3_CAN82106 hypothetical protein [Vitis vinifera] 662 bp

MTSQVVKVRRETIAACMTCPLCNKLLKEATTISLCLHTFCRKCIYEKLSDEEVDCCPVCN

IDLGCVPVDKLRPDHNLQDIRAKIFPFKRRKISVPQVMPPIPLPVKRKERSLSSLVVSTP

RVSMQTGLTGRRTKAVARKSTALRGSSFSIVEPIKKEEDSVEDCPESSSSPESRNKVAQT

KKQNSSISEASKDQKPNKDTENDADPWDGKVDLWTPLNCLVEAANRTKSSKFNSQGTSLA

KSEPFNAPDSEVYMPKTKAKAEPLNAADGEVCIPKTKIKEHGHKIKVQDDKNGAALLPGP

VKRRRMRAVGRKRISASGEMCTPAQVVLSAAGAKRGRRNCPIWFSLVASENQGGNGPLSK

IPAYYLRVKDGNLPVSFIQKYLVKKLDLTNEAEVEIRCQGEAVVPTLQLQKLVELWLRTA

STSKRVATSVGTSAKEFVMSGGSETEVTWEDQQNINKFGRLNNRFHELEDEIKMAKICTR

GVTGDHSYPLLRAYSKHEEAGLANEIEILALLDNMLQAKALCLSNLIVEGDFNTVISWMS

NRERGPWKFVKWMHKIIDIASELGCSFSLAPCSANEETNENLEDASNELILTDEEMVRFQ

IGEVFAHVPKEEVEERIEQMKEVTSKKLEKLEEEKDSVVAQMAELKKILYGKFKESINLE

ED

Family: VOZ

>VOZ_gi|145324058|ref|NP_001077618_1| gi|145324058|ref|NP_001077618.1| VOZ1 (VASCULAR PLANT ONE ZINC FINGER PROTEIN); transcription activator [Arabidopsis thaliana] 671 bp

MTGKRSK-------------TNCRSASHKLFKDKAKNRVDDLQGMLLDLQFARKESRPTD

VTLLEEQVNQMLREWKSELNEPSPASSLQQGGTLGSFSSDICRLLQLC---DEEDDATSK

LAAP--------------------------------------------------------

---KPEPADQNL---------------------------EAGKAAVFQRGYNLVQGKSE-

HGLPLVDNCKDLSLA-----AGNNFDG---TAPLEYHQQYD-----LQQEF--EPNFNGG

FNNCPSYGVVEGPI--HISNFIPTICPPPSAFLGPKCALWDCPRPAQGFDWFQDYCSSFH

AALAFN-EGPPGMNPVVRPGGIGLKDGLLFAALSAKAGGKDVGIPECEGAATAKSPWNAP

ELFDLTVLESETLREWLFFDKPRRAFESGNRKQRSLPDYNGRGWHESRKQIMVEFGGLKR

SYYMDPQPLHHFEWHLYEYEINKCDACALYRLELKLVDGKKTSKGKVSNDSVADLQKQMG

--RLTAEFPPENNTTNTTNNNKRCIKGRPKVSTKVATGN---VQNTVEQ-----------

--------------------------ANDY---GVGEEFNYLVGNL--------------

SDYYIP-----

>VOZ_gi|57899607|dbj|BAD87186_1| gi|57899607|dbj|BAD87186.1| putative vascular plant one zinc finger protein [Oryza sativa Japonica Group] 671 bp

M----------GRGPAAGR-SSAGAARHQQFRARAKTRVDDLQEMFSGLQSARKEARSTD

AAVLEAQLHQMLREWRAELS---QQGNNRE---LSDPPSETLRLLQLAAAEEEEDDATSK

LVEQQQQHP------------------------------------PSANQAHGHPQAQGG

QDMKPEPPEEAVASPADLTVPQQPQSPGQGVLASGGGMLAPAAAAVFHDQMYYVNQELT-

VEDFLYDDNYKMYLPGYNSDVLNNLES---TGQLEY-PQFN-----LPQEL--PPNAYLD

TSNC---GQNAGDVFLHMSDLLNTMSPVPAAFLRPKCALWDCPRPAQGSERWQDYCSMYH

ADLAVKEEGPPGTMPVIRPRGIDLKDGPLFAALSAKIQGKHVGIPVCEGAATAKSPWNAP

ELFDLYIFEGESIREWLFFDKPRRAFESGNRKQRSLPDYNGRGWHESRKQVMKDFGGLKR

SYYMDPQPSNSYEWHLYEYEINDCDAFALYRLEFKSSDAKKTAKSKLACNPLNEIQQQMV

--RLSADSPVE---------NKRSARSRTKANPNDINSNIYLVQNTTVQGSI--------

--------------------------PNAYQAVSQPDQMTYLNGNVVYGPHLPYGYSTER

SDFYWSSNDGA

>VOZ_gi|51451360|gb|AAU03115_1| gi|51451360|gb|AAU03115.1| unknown protein [Oryza sativa Japonica Group] 671 bp

MAGDPAAGGDGGRGSSGGKGSSPSSSRHQQFRNLAKTRVDDLQEMFSGLQSARKESRSAD

AALLEEQVHHMLREWRAELNNSQSQGNNRE---ASDPPSETLRLLQLAGA-EEEDDATSK

LVMPRSPMPMQSSHEGHNLSPVLQGGTMAGGAAELMVPRSPLQQMPSSHQSHGHGQ-DGG

QNLQ---GEAVMGSTAATAAPHL----GQGMQGDCGGM-AGVTNAMFHDQLYYIDHELN-

IDDFLQDDDYKINLPGSNPDGPNTMQG---IGQLEH-QQYN-----LPLDL--PPNSFVD

ANNS---AQSSGDVFFHMSDLLTTMCPSPSQYLGPKCALWDCGRPVRGSDECQHYCNPYH

AGLALNDDGLLGTRPVMRPRGIDLKDGPLFAALSAKVQGKNVGIPVCEGAATTKSPWNAP

ELFDLSLLEGESLREWLFFDTPRRAFDSGNRKQRSLPDYNGRGWHESRKQVMKDFGGLKR

SYYMDPQPSSNYEWHLFEYETNDSDALALYRLEYKSSDTKRSVKSKLASSPLSEIQQQMV

--RLSADSPVE---------SKRTARSRAKANQKDNNSNAYPALNTPVQVSASNAHQTMS

VNTPDQVNVSNAYQTMPLNTPNQPGPSNAYHAASQMDQMTFLDGSVVYGPHLPYGYSTER

SDFYWNPSDGT

>VOZ_gi|18405962|ref|NP_565972_1| gi|18405962|ref|NP_565972.1| VOZ2 (VASCULAR PLANT ONE ZINC FINGER PROTEIN 2); transcription activator [Arabidopsis thaliana] 671 bp

MSNHPKI-----------------TSAHQNVEEKLR----ELQERFCHLQAARKEGRHGD

LALLEAQISQNIREWQAELTAPSPESSL-LGEGISQFLEEFAPLLKL----DEEDDATST

LKEHAGAKP---------------------------------------------------

---DPEGFSQSL-CPPEWTSENFS---------------QSPFNGNFSCGFEDALNSTET

HGQQLHYG-YEGFDPSIN--SAPDFHDQKLSSNLDITSQYDYIFSEVRQELDNSPSTKLD

SSE-------EIDNFAEFSTPSSVRVP-PSAFLGPKCALWDCTRPAQGSEWYLDYCSNYH

GTLALNEDS-PGTAPVLRPGGISLKDNLLIDALRAKTQGKNVGIPVCEGAVNTKCPWNAA

ELFHLELVEGETIREWLFFDKPRRAYDSGNRKQRSLPDYSGRGWHESRKQLMKEQEGQKR

SYYMDPQPPGPFEWHLFEYQINESDACALYRLELKVGNGKKSPKGKISKDPLADLQKKMG

QFKVASDKPSP---------PTKGRKE---------------------------------

------------------------------------------------------------

-----------
