# Supplementary material for: Identification of Transcription Factor Genes and Their Correlation with the High Diversity of Stramenopiles
Source: PLoS One. 2014 Nov 6;9(11):e111841. doi: 10.1371/journal.pone.0111841 (PMC4222949; doi:10.1371/journal.pone.0111841)
Supplement: Figure S1 — Bipartite graph of rules for identification and classification of TFs and TRs. Blue squares represent TF families and stripped squares represent TR families. Domains are represented as circles in which yellow circles represent PFAM domains and orange circles represent in house domains. Moreover, continuous lines indicate required domains and dotted lines indicate forbidden domains. Updated from Riaño-Pachón et al., 2007. (PDF) [file pone.0111841.s004.pdf]

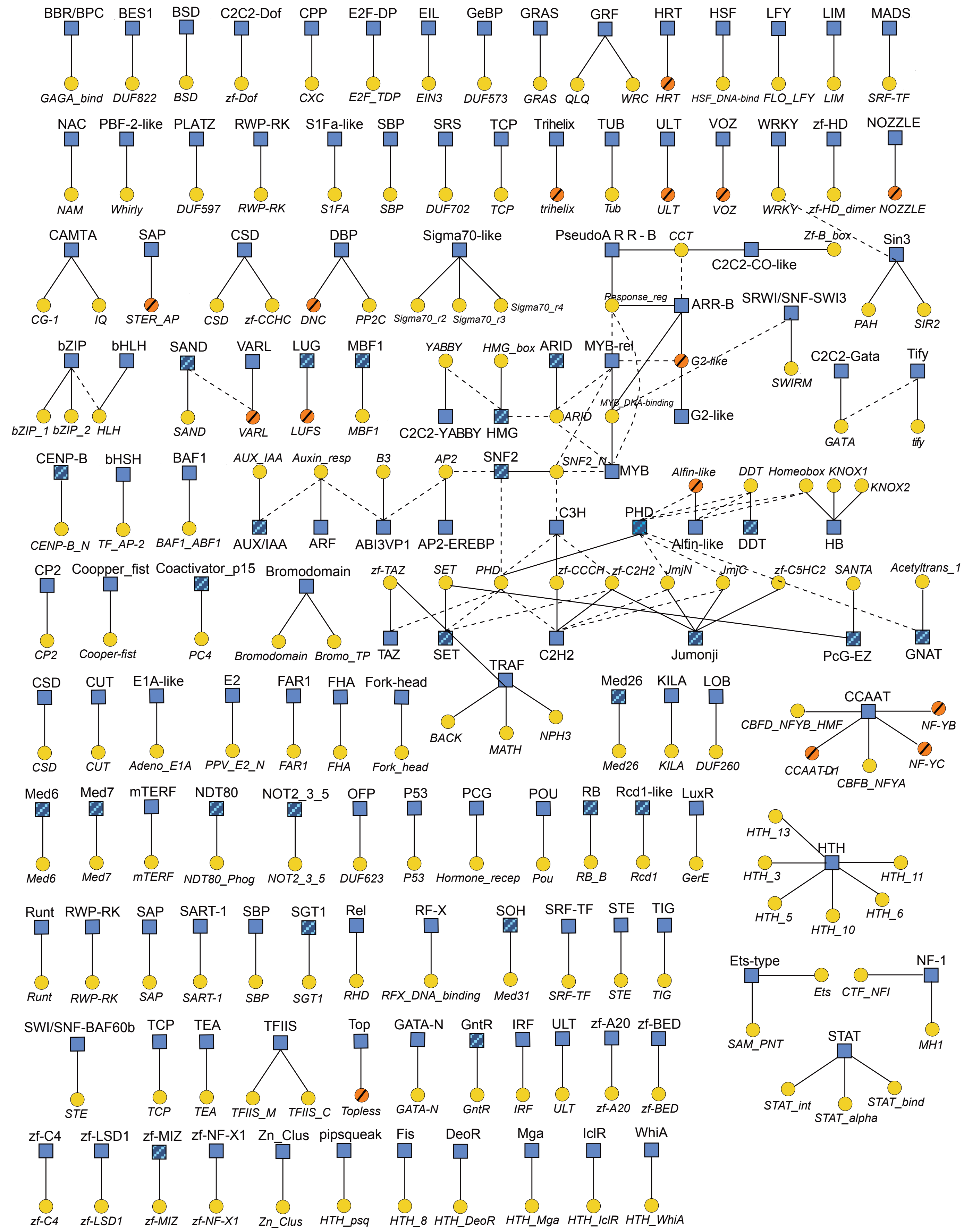

Supplementary figure 1

A bipartite graph that describes the rules for identification and classification of TFs and TRs

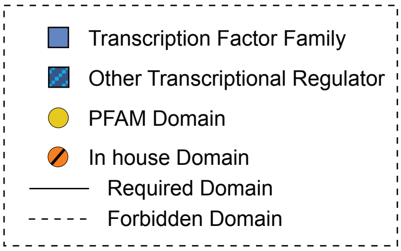

Modified from Riaño-Pachón et al. 2007. BMC Bioinformatics 8:42
