# Supplementary figures and images for: Identification of Transcription Factor Genes and Their Correlation with the High Diversity of Stramenopiles
Source: PLoS One. 2014 Nov 6;9(11):e111841. doi: 10.1371/journal.pone.0111841 (PMC4222949; doi:10.1371/journal.pone.0111841)

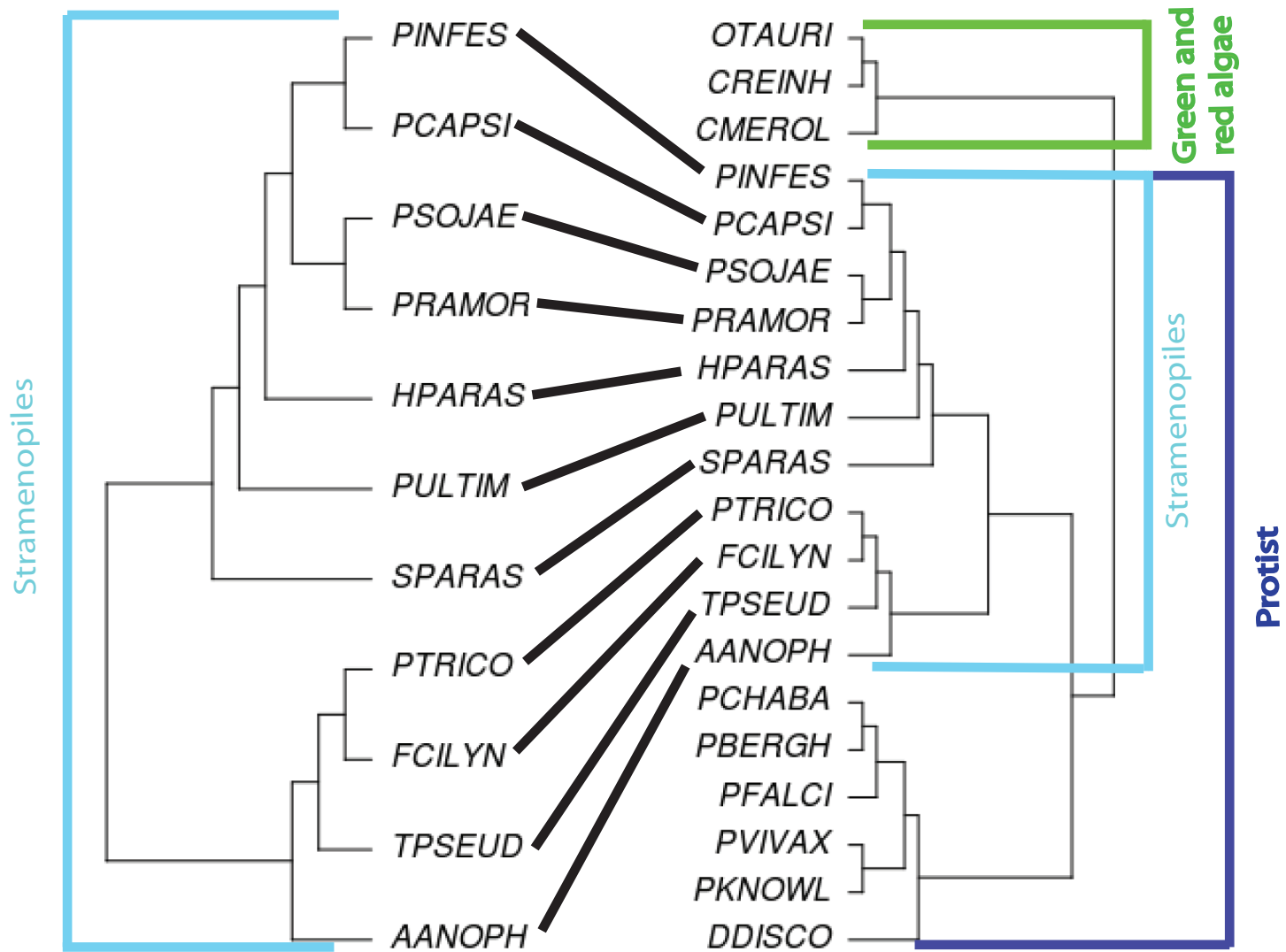

Supplement: Figure S2 — Tanglegram to assess the effect of inclusion of distantly related species. Right: Phylogenomic reconstruction of Stramenopiles. Left: Phylogenomic reconstruction of Stramenopiles plus another protists and algae. The inclusion of another protists such as Plasmodium spp. and red and green algae in the phylogenomic reconstruction, does not affect the evolutionary relationships between Stramenopiles. Acronyms used: Phytophthora infestans – PINFES, Phytophthora capsici – PCAPSI, Phytophthora sojae – PSOJAE, Phytophthora ramorum – PRAMOR, Hyaloperonospora parasítica – HPARAS, Pythium ultimum – PULTIM, Saprolegnia parasítica – SPARAS, Phaeodactylum tricornutum – PTRICO, Fragilariopsis cylindrus – FCYLIN, Thalassiosira pseudonana – TPSEUD, Aureococcus anophagefferens – AANOPH, Plasmodium bergheu – PBERGH, Plasmodium chabaudi – PCHABA, Plasmoduim falciparum – PFALCI, Plasmodium knowlesi – PKNOW, Plasmodium vivax – PVIVAX, Dictyostelium discoideum – DDISCO, Cyanidioschyzon merolae – CMEROL, Ostreocuccus tauri – OTAURI, Clamydomonas reinhardtii – CREINH. (PDF) [file pone.0111841.s005.pdf]

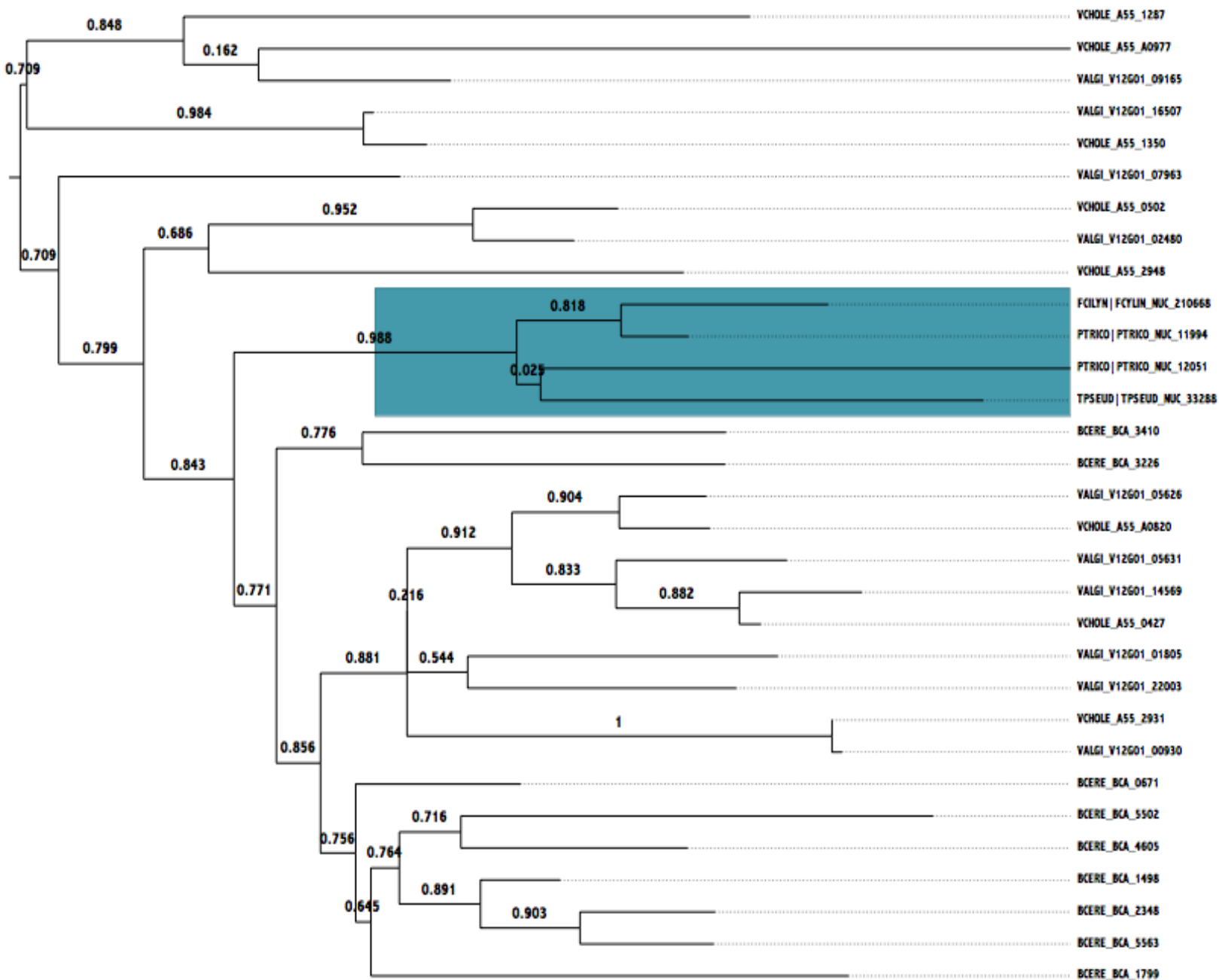

0.2

Supplement: Figure S3 — LuxR phylogeny. A maximum likelihood analysis via FastTree with 1000 bootstrap replicates was performed with proteins classified into the LuxR family from Stramenopiles (Phaeodactylum tricornutum, Thalassiosira pseudonana and Fragilariopsis cylindrus) and 3 selected bacteria (Vibrio cholera, Vibrio alginolyticus and Bacillus cereus). Highlighted in blue all the proteins identified in Stramenopiles that belong to the LuxR family. (PDF) [file pone.0111841.s006.pdf]
